# Integrative species delimitation and five new species of lynx spiders (Araneae, Oxyopidae) in Taiwan

**DOI:** 10.1371/journal.pone.0301776

**Published:** 2024-05-09

**Authors:** Ying-Yuan Lo, Ren-Chung Cheng, Chung-Ping Lin

**Affiliations:** 1 Department of Life Science, National Taiwan Normal University, Taipei, Taiwan; 2 Wild Animals Division, Biodiversity Research Institute, Nantou, Taiwan; 3 Department of Life Sciences, National Chung Hsing University, Taichung, Taiwan; 4 Research Center for Global Change Biology, National Chung Hsing University, Taichung, Taiwan; Laboratoire de Biologie du Développement de Villefranche-sur-Mer, FRANCE

## Abstract

An accurate assessment of species diversity is a cornerstone of biology and conservation. The lynx spiders (Araneae: Oxyopidae) represent one of the most diverse and widespread cursorial spider groups, however their species richness in Asia is highly underestimated. In this study, we revised species diversity with extensive taxon sampling in Taiwan and explored species boundaries based on morphological traits and genetic data using a two-step approach of molecular species delimitation. Firstly, we employed a single *COI* dataset and applied two genetic distance-based methods: ABGD and ASAP, and two topology-based methods: GMYC and bPTP. Secondly, we further analyzed the lineages that were not consistently delimited, and incorporated *H3* to the dataset for a coalescent-based analysis using BPP. A total of eight morphological species were recognized, including five new species, *Hamataliwa cordivulva* **sp. nov.**, *Hamat*. *leporauris* **sp. nov.**, *Tapponia auriola* **sp. nov.**, *T*. *parva* **sp. nov.** and *T*. *rarobulbus* **sp. nov.**, and three newly recorded species, *Hamadruas hieroglyphica* (Thorell, 1887), *Hamat*. *foveata* Tang & Li, 2012 and *Peucetia latikae* Tikader, 1970. All eight morphological species exhibited reciprocally monophyletic lineages. The results of molecular-based delimitation analyses suggested a variety of species hypotheses that did not fully correspond to the eight morphological species. We found that *Hamat*. *cordivulva* **sp. nov.** and *Hamat*. *foveata* showed shallow genetic differentiation in the *COI*, but they were unequivocally distinguishable according to their genitalia. In contrast, *T*. *parva* **sp. nov.** represented a deep divergent lineage, while differences of genitalia were not detected. This study highlights the need to comprehensively employ multiple evidence and methods to delineate species boundaries and the values of diagnostic morphological characters for taxonomic studies in lynx spiders.

## Introduction

**Fig 1:**
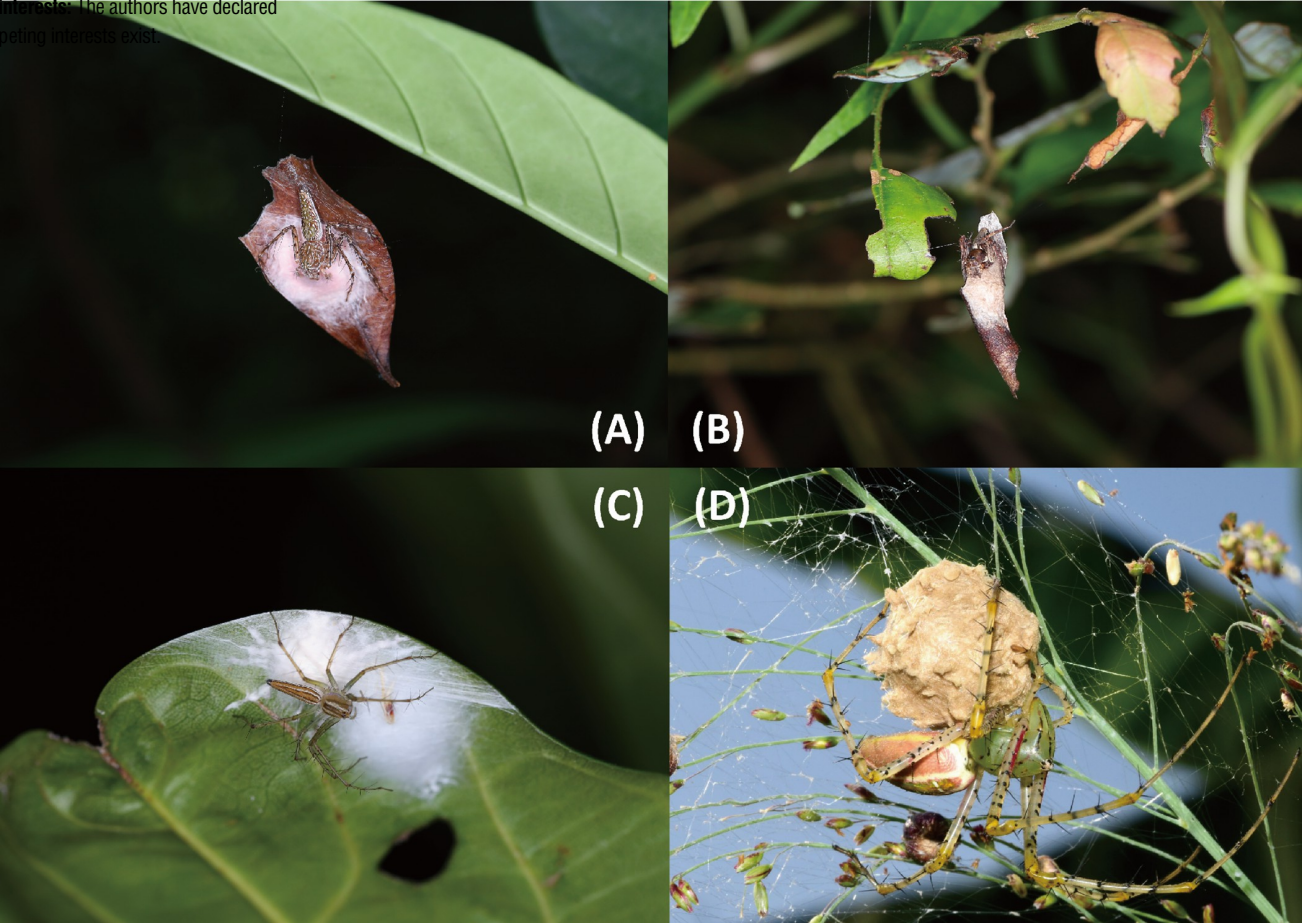
Maternal egg-guarding behavior of oxyopids. A, *Hamadruas hieroglyphica*, 2014 Oct. 28, Fuyang National Park, Taipei; B, *Hamataliwa cordivulva* **sp. nov.**, 2014 Aug. 18, Taichung; C, *Oxyopes macilentus*, 2014 Oct. 20, Qipan village, Yunlin; D, *Peucetia latikae*, 2018 Oct. 09, Dakeng, Taichung (photo by Sung-Yan Wei).

The family Oxyopidae Thorell, 1879, usually called lynx spiders, comprises nine genera and 446 species [1] that are found almost everywhere. Oxyopids possess several unique diagnostic characters, including a high clypeus, four rows of eyes with the anterior eye row (AER) recurved and the posterior eye row (PER) procurved, legs adorned with numerous conspicuous and erect spines, and a body covered with appressed setae, that make them easily be distinguishable from other families [2]. These spiders come in a variety of sizes, from small to large. These cursorial predators inhabit open grasslands, shrubs or canopy, and hunt various invertebrates including bees, butterflies, grasshoppers, and flies using ambush or stalking tactics [3,4]. Oxyopids display a variety of maternal egg-guarding behavior. For example, *Hamadruas* and *Hamataliwa* produce an egg sac attached to a dried leaf hanged on by a few lines, whereas *Oxyopes* directly attaches an egg sac to the vegetation (Fig 1, personal observation), and *Peucetia* suspends the egg sac with tangled silks extending to nearby leaves or stems and holds the sac under it [5,6].

Within Eastern and Southern Asia, five genera of oxyopids are recognized: *Hamadruas* Deeleman-Reinhold, 2009, *Hamataliwa* Keyserling, 1887, *Oxyopes* Latreille, 1804, *Peucetia* Thorell, 1869 and *Tapponia* Simon, 1885. Despite this, there is a noticeable deficiency in regional taxonomic reviews, a shortcoming that extends to Taiwan as well [7,8]. The earliest known record of an oxyopid species in Taiwan is *Peucetia formosensis* Kishida, 1930 [9]. Subsequently, *Oxyopes sertatus* L. Koch, 1878, *O*. *macilentus* L. Koch, 1878 and *O*. *sushilae* Tikader, 1965 were documented between 1936 and 2016 [10–12]. Recently, a comprehensive review of the genus *Oxyopes* in Taiwan was conducted by Lo et al. [13], which described two new species, *O*. *hasta* and *O*. *taiwensis*, and newly recorded two species, *O*. *fujianicus* and *O*. *striagatus*. As a result, the official record so far includes two genera and eight species of lynx spiders for the island. However, based on our field survey, three genera, *Hamadruas*, *Hamataliwa*, and *Tapponia* were newly discovered, which indicates that the species diversity of Taiwanese oxyopids may be underestimated.

Oxyopidae genera can be diagnosed based on their body size, eye arrangement, leg formation, and most significantly, their genitalia [7,14]. For example, the three newly discovered genera can be easily distinguished from *Oxyopes* and *Peucetia* by their male palp organ with a basal apophysis on cymbium and with a tegular lobe. Nevertheless, species within a genus are primarily identified based on genital features due to the absence of external diagnostic features. The shape of retrolateral tibia apophysis (RTA) and median apophysis of male palp organ, and the shape or location of spermatheca and copulatory ducts in the vulva are the most important diagnostic characters.

An accurate assessment of species diversity is a cornerstone of biology and conservation [15–18]. In the face of increasing exploitation pressures and continued loss of biodiversity, DNA data have proven useful in accelerating species biodiversity surveys, especially for hyper-diverse taxa whose morphological identification is challenging [19–22]. Recently, the delineation of species boundaries has been predominantly achieved using an integrative approach that incorporates morphometric, genetic, and ecological information from various sources [23,24]. However, different species delimitation methods based on differing theoretical principles or species concepts usually produced different results [25–28]. Herein, we applied morphological and genetic data to evaluate the species boundaries of lynx spiders under morphological, phylogenetic and genetic species concepts. In addition, to produce a robust and time efficient assessment, we also used a two-step approach [29,30], which involves rapid identification of lineages based on a single molecular marker using various species delimitation methods suited to single loci analysis, followed by a secondary analysis utilizing additional markers for lineages that were not consistently delineated in the initial analysis.

The objective of this study is to conduct a review of Taiwanese lynx spiders using an integrative approach that combines morphological examination and multiple molecular species delimitation methods through a two-step approach to discover and describe new species (excluding the genus *Oxyopes*). Ultimately, the consistency of these methods was evaluated, leading to the description of five new species and three newly recorded species.

## Materials and methods

### Taxon sampling and morphological measurements

**Fig 2:**
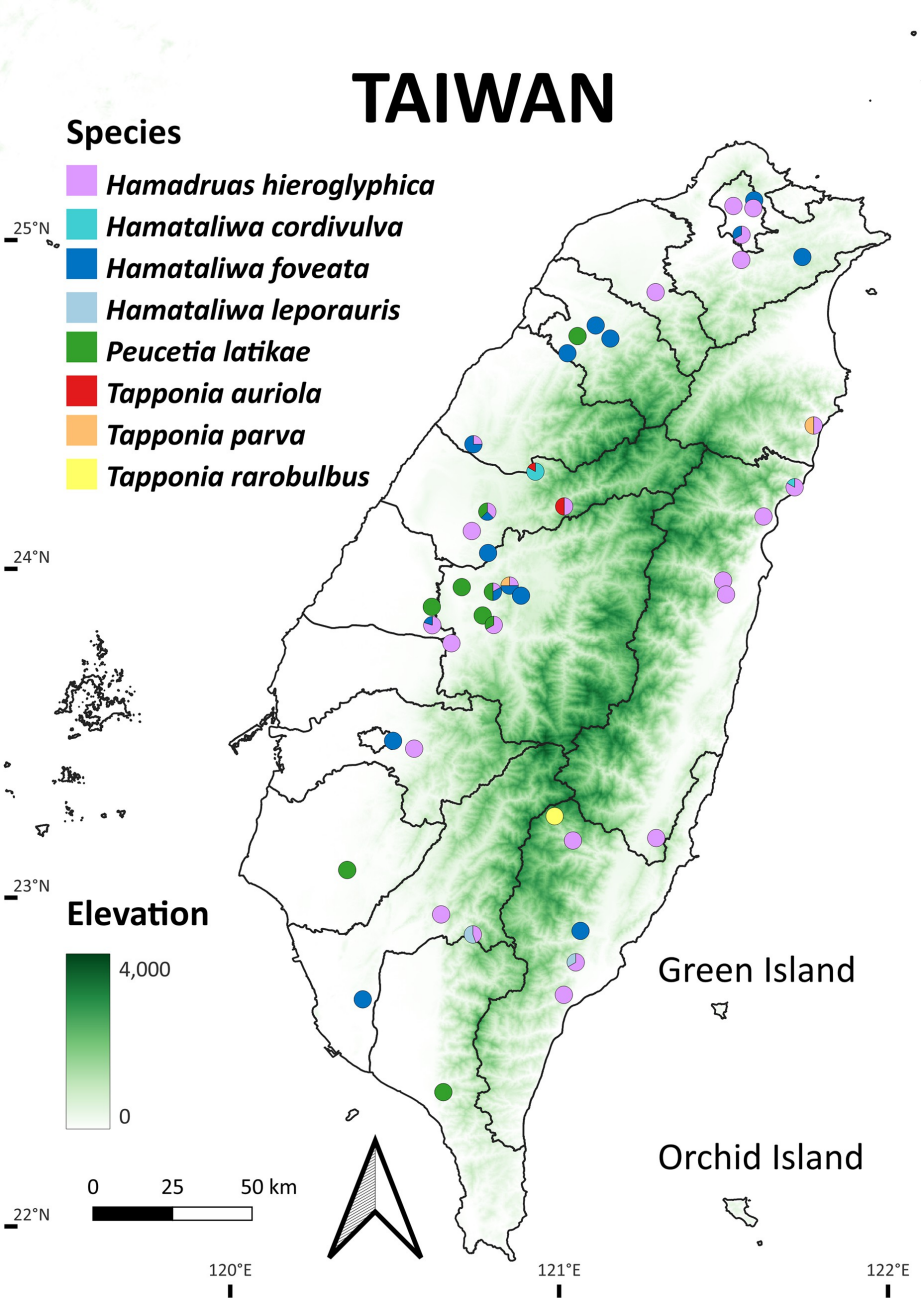
Sampling sites of oxyopids from Taiwan in this study. The elevation layer was obtained from https://data.gov.tw/dataset/35430 under a CC BY license, with permission from Ministry of Digital Affairs, original copyright 2016.

Fresh spider specimens from various localities in Taiwan were collected by hand or using sweeping nets (Fig 2). All specimen collections in protected areas (Yangmingshan National Park) were conducted following legal applications and were approved by Yangmingshan National Park Headquarters. After collection, specimens were preserved in 75% ethanol. Selected specimens were subsequently dissected for genital examination. The female epigyne and inner genital structures were cleaned using a heated 10% KOH solution. The majority of the setae on the male palp’s cymbium were removed to facilitate the observation of fine structures, resulting in their absence in the illustrations. Based on examination of morphological characters, we identified eight distinct species from Taiwanese specimens. Morphological measurements were obtained using a micrometer mounted on the eyepiece of the stereomicroscope. All measurements are given in millimeters. The measurements of the pedipalps and legs are given as the total length (pedipalp: femur, patella, tibia, and tarsus lengths; leg: femur, patella, tibia, metatarsus, and tarsus lengths). The holotype and paratypes are deposited in National Museum of Natural Science (**NMNS**), and other voucher specimens are deposited in Taiwan Biodiversity Research Institute (**TBRI**).

The abbreviations followed Lo et al. [13]: AER, anterior eye row; ALE, anterior lateral eye; AME, anterior median eye; AME-I, interdistance between anterior median eyes; AML-I, interdistance between anterior median and lateral eyes; CD: copulatory duct; C: conductor; CO: copulatory opening; E: embolus; FD: fertilization duct; MA, median apophysis; MOA, median ocular area; MOA-AW, anterior width of median ocular area; MOA-L, length of median ocular area; MOA-PW, posterior width of median ocular area; PER, posterior eye row; PLE, posterior lateral eye; PME, posterior median eye; PME-I, interdistance between posterior median eyes; PML-I, interdistance between posterior median and lateral eyes; dRTA: dorsal retrolateral tibial apophysis; vRTA, ventral retrolateral tibial apophysis; S, spermatheca; SE, sclerotized edge.

### DNA extraction and sequencing

For DNA extraction,1–2 legs from each specimen were preserved in 95% ethanol and stored at -20°C. The genomic DNA was extracted using FavorPrep^TM^ Tissue Genomic DNA Extraction Mini Kit (Favorgen Biotech, Ping-Tung, Taiwan). The partial mitochondrial cytochrome oxidase subunit 1 (*COI*) and nuclear histone *H3* sequences were amplified using primers, LCO-1490 and CR-2 [31,32] and H3F1 and H3R1 [33]. The PCR protocol for *COI* followed Lo et al. [13]. For *H3*, PCR thermos-profile was set with an initial denaturing at 95°C for 3 min, followed by 25 cycles of denaturing at 95°C for 30 sec, annealing at 56°C for 45 sec, elongation at 72°C for 45 sec, and 10 cycles with annealing at 50°C, and a final extension at 72°C for 7 min. The PCR products were sequenced by Genomics BioSci & Tech. Co., Ltd. (New Taipei City, Taiwan) using an ABI 3730xl DNA Analyzer.

Sequence contigs of each sample were trimmed and edited using Sequencher version 5.4.5. We downloaded DNA sequences of related species (*Hamataliwa* spp.) from GenBank [34], and the accessions are listed in S1 Table. Four wolf spider species (Lycosidae), *Hippasa holmerae* Thorell, 1895, *Hogna arborea* Lo, Wei & Cheng, 2023, *Lycosa coelestis* L. Koch, 1878, and *Pardosa laura* Karsch, 1879, were selected as outgroups. The multiple sequence alignment was performed using MAFFT [35,36] plugin in Geneious Prime® 2023.0.1 with the “Auto” option selected to find the best alignment strategy and the default option for other settings.

### Phylogenetic analysis

For phylogeny analysis, the best-fitting substitution model was estimated using ModelFinder [37] in IQ-TREE 2 [38] with -m MFP option and three partitions was used as input: first, second, and third codon position for *COI*. The HKY+F+G4 was selected as best model for partition 1–2, and the TIM3+F+I was selected as best model for partition 3. Maximum Likelihood (ML) analysis was performed in IQ-TREE 2 [38], with node supports estimated using bootstrap and SH-like approximate likelihood ratio test (SH-aLRT) with 1000 replicates. Bayesian inference (BI) analysis was conducted in MrBayes 3.2.7 [39] using 2,000,000 generations of Markov Chain Monte Carlo (MCMC) iterations, with tree sampling of every 500 generations and the initial 25% of generations discarded as burn-in. We checked the average standard deviation of split frequencies to be lower than 0.01 after 960,000 generations. Convergence of the MCMC runs was confirmed with effective sample sizes (ESS) of the model parameters greater than 200 in Tracer v.1.7.2 [40].

### Molecular species delimitation

To explore the average intra- and interspecific genetic distances, we treated the eight morphological species as candidate species for molecular data verification. The distances were calculated with Kimura two-parameter (K2P) model using the ‘*ape*’ [41], pegas [42], and spider [43] packages in R v4.2.2 [44]. Due to the clear morphological distinction and distant relatedness between *Peucetia* and the genera *Hamadruas*, *Hamataliwa* and *Tapponia*, we excluded *Peucetia* from subsequent molecular species delimitation analyses.

To ensure accuracy and consistency of delimitation results, we integrated a two-step approach of species delimitation with morphological identifications. Initially, we utilized a single locus *COI* dataset, which encompassed 79 terminals with 661 base pairs. This dataset was subjected to the genetic distance-based methods Automatic Barcode Gap Discovery (ABGD) [45] and Assemble Species by Automatic Partition (ASAP) [46]. Additionally, we employed topology-based methods such as generalized mixed Yule-coalescent (GMYC) [47] and Bayesian implementation of the PTP (bPTP) [48]. In the second step, for those lineages not consistently defined, we incorporated *H3* sequences to the dataset, facilitating a deeper, coalescent-based evaluation using Bayesian Phylogenetics and Phylogeography (BPP) [22].

The genetic distance-based analyses do not require prior species assignment. The ABGD analysis was carried out online (https://bioinfo.mnhn.fr/abi/public/abgd/) under JC69 and K2P model, respectively, with a minimum intraspecific variability Pmin = 0.001, maximum intraspecific variability Pmax = 0.2, step = 20, and other default settings. ASAP propose species partitions using a hierarchical clustering algorithm based on pairwise genetic distances. The analysis was carried out online (https://bioinfo.mnhn.fr/abi/public/asap/asapweb.html) under the same models as in the ABGD method with default settings.

For GMYC analysis, an ultrametric tree was required. To generate this, we implemented a Bayesian analysis in BEAST v2.6.3 [49], employing a strict clock model with either Yule or a coalescent constant population model. The posterior probability was estimated by MCMC algorithm, with tree sampling every 1000 generations over a total of 30 million generations. MCMC convergence and sufficient sampling were assessed in Tracer v.1.7.2 [50]. The maximum clade credibility tree was constructed in TreeAnnotator v2.4.5 by discarding 10% of the sampled trees as burn-in. This guide tree was then inputted for GMYC analysis using a single-threshold model via the ‘*split*’ package [51] in R v4.2.2 [44].

The bPTP, a derivative of PTP [48], introduces Bayesian support (BS) values to delimit species with an input tree. We used a maximum-likelihood phylogenetic tree as the input tree. We implemented bPTP on an online server (https://species.h-its.org/ptp/) with 500,000 MCMC generations, sampling every 100 trees and discarded the first 10% of samples as burn-in.

The multispecies coalescent species delimitation was conducted using BPP v4.6.2 [52]. This program explores and estimates different species models by changing the topology of the species tree through reversible-jump Markov chain Monte Carlo (rjMCMC) algorithm. We tested six different species models ranging of 2–5 species (Table 1), by splitting or lumping *Hamat*. *cordivulva* **sp. nov.** and *Hamat*. *foveata* as well as *T*. *parva* **sp. nov.**, as these three lineages were not consistently delimited in previous species delimitation analyses. The A11 analysis (joint species delimitation and species tree estimation) was performed using four different combinations of prior sets. An inverse-gamma distribution with *α* = 3 was used as a diffuse prior for both population size parameter (*θ*) and age of the root of species tree (*τ*), and adjusted *β* according to mean (*m*) estimation of nucleotide diversity (for *θ*) and node height (for *τ*). This was calculated using the equation *m* = *β*/(*α*-1), for *α* > 2 [26,52]. The different prior means was set to vary by one order of magnitude to present population size larger or smaller, and divergence time deeper or shallow. The rjMCMC algorithm 1 (*a* = 2, *m* = 1) was used for analyses, running for 220,000 MCMC generations, sampling every two generations, with the first 20,000 samples discarded as burn-in. Each prior set was run twice to check for convergence. Finally, we compared posterior probability (PP) among different models and prior sets. A PP value ≥ 0.95 was considered to indicate high support, while a 0.95 > PP ≥ 0.90 was considered to indicate moderately support and a PP < 0.90 was considered week support [26].

**Table 1:** Results of Bayesian species delimitation (BPP) for six species hypothesis model under different prior settings.

Model	Prior (θ)	Prior (τ)	Species	Posterior Probability	Delimitation
Model 1	IG(3, 0.004)	IG(3, 0.004)	3	1.00 | 1.00	C, F, P
(3 sp.)	IG(3, 0.04)	IG(3, 0.04)	3	1.00 | 1.00	C, F, P
	IG(3, 0.004)	IG(3, 0.04)	3	1.00 | 1.00	C, F, P
	IG(3, 0.04)	IG(3, 0.004)	3	1.00 | 1.00	C, F, P
Model 2	IG(3, 0.004)	IG(3, 0.004)	2	1.00 | 1.00	C+F, P
(2 sp.)	IG(3, 0.04)	IG(3, 0.04)	2	1.00 | 1.00	C+F, P
	IG(3, 0.004)	IG(3, 0.04)	2	1.00 | 1.00	C+F, P
	IG(3, 0.04)	IG(3, 0.004)	2	1.00 | 1.00	C+F, P
Model 3	IG(3, 0.004)	IG(3, 0.004)	4	1.00 | 1.00	C, F1, F2, P
(4 sp.)	IG(3, 0.04)	IG(3, 0.04)	4	0.83 | 0.82	C, F1, F2, P
	IG(3, 0.004)	IG(3, 0.04)	4	1.00 | 0.99	C, F1, F2, P
	IG(3, 0.04)	IG(3, 0.004)	4	0.92 | 0.92	C, F1, F2, P
Model 4	IG(3, 0.004)	IG(3, 0.004)	4	0.57 | 0.57	C, F, P1, P2
(4 sp.)	IG(3, 0.04)	IG(3, 0.04)	3	0.89 | 0.88	C, F, P
	IG(3, 0.004)	IG(3, 0.04)	3	0.83 | 0.81	C, F, P
	IG(3, 0.04)	IG(3, 0.004)	3	0.69 | 0.7	C, F, P
Model 5	IG(3, 0.004)	IG(3, 0.004)	3	0.53 | 0.51	C+F, P1, P2
(3 sp.)	IG(3, 0.04)	IG(3, 0.04)	2	0.89 | 0.88	C+F, P
	IG(3, 0.004)	IG(3, 0.04)	2	0.81 | 0.81	C+F, P
	IG(3, 0.04)	IG(3, 0.004)	2	0.75 | 0.85	C+F, P
Model 6	IG(3, 0.004)	IG(3, 0.004)	5	0.57 | 0.58	C, F1, F2, P1, P2
(5 sp.)	IG(3, 0.04)	IG(3, 0.04)	4	0.72 | 0.77	C, F1, F2, P
	IG(3, 0.004)	IG(3, 0.04)	4	0.81 | 0.77	C, F1, F2, P
	IG(3, 0.04)	IG(3, 0.004)	4	0.64 | 0.65	C, F1, F2, P

Posterior probabilities are provided for two runs. Abbreviations: C–*Hamataliwa cordivulva*; F–*Hamataliwa foveata* (F1 and F2 represent two clades); P–*Tapponia parva* (P1 and P2 represent two clades). The topologies of different models as follow: Model 1: ((C, F), P); Model 2: (C+F, P); Model 3: ((C, (F1, F2)), P); Model 4: ((C, F), (P1, P2)); Model 5: (C+F, (P1, P2)); Model 6: ((C, (F1, F2)), (P1, P2)).

## Results

### Morphological species

In total, we identified eight morphological species in the genera *Hamadruas*, *Hamataliwa*, *Peucetia* and *Tapponia* in Taiwan, including five new species (*Hamat*. *cordivulva* **sp. nov.**, *Hamat*. *leporauris* **sp. nov.**, *T*. *auriola* **sp. nov.**, *T*. *parva* **sp. nov.** and *T*. *rarobulbus* **sp. nov.**) and three newly recorded species (*Hamad*. *hieroglyphica* (Thorell, 1887), *Hamat*. *foveata* Tang & Li, 2012, and *P*. *latikae* Tikader, 1970). The detailed diagnosis, descriptions, and illustrations are provided in the Taxonomy section. Among these morphological species, the mean intraspecific distances ranged from 0.05% to 4.77% and interspecific distances ranged from 1.88% to 18.33% (Table 2). Excluding one pair, *Hamat*. *cordivulva* **sp. nov.** and *Hamat*. *foveata* (1.88%), all mean interspecific divergences surpassed 5.77%. Conversely, all mean intraspecific divergences were below 1.17%, with the exception of *T*. *parva* **sp. nov.** (4.77%).

**Table 2:** The mean intraspecific (diagonal) and interspecific (lower triangular) genetic distances (%) of eight species of Oxyopidae from Taiwan.

	*hieroglyphica*	*cordivulva*	*foveata*	*leporauris*	*latikae*	*auriola*	*parva*	*rarobulbus*
*Hamadruas hieroglyphica*	0.09							
*Hamataliwa cordivulva*	8.53	0.10						
*Hamataliwa foveata*	9.55	1.88	0.95					
*Hamataliwa leporauris*	8.30	5.77	6.65	0.10				
*Peucetia latikae*	16.04	16.30	17.56	15.65	0.05			
*Tapponia auriola*	11.40	9.31	9.53	10.10	18.33	1.17		
*Tapponia parva*	11.34	9.20	9.70	9.33	15.87	10.78	4.77	
*Tapponia rarobulbus*	11.33	8.49	9.68	8.78	16.43	11.87	10.56	—

The genus names in first rows are omitted.

### Molecular species delimitation

**Fig 3:**
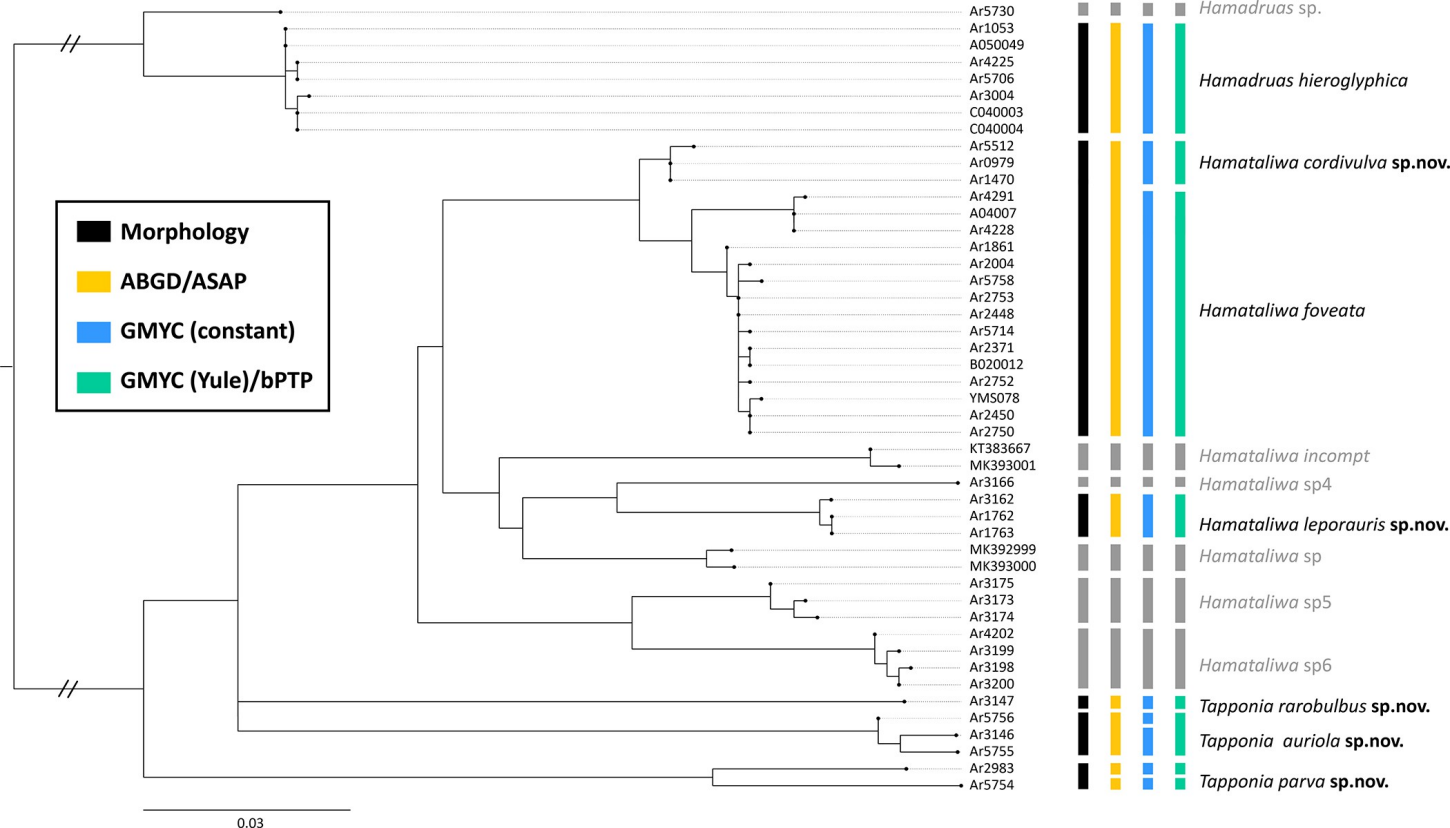
Maximum likelihood phylogenetic tree and the species delimitation of *Hamadruas*, *Hamataliwa*, and *Tapponia* based on morphology and *COI* dataset. The bars represent results obtained from different methods. Species that are not distributed in Taiwan are labeled in grey.

Different molecular species delimitation methods based on *COI* data have yielded between seven and ten species. The main conflicts arise from the defining the species boundaries of *Hamat*. *cordivulva* **sp. nov.**, *Hamat*. *foveata*, *T*. *auriola* **sp. nov.** and *T*. *parva* **sp. nov.** (Fig 3).

Both ABGD and ASAP with JC69 and K2P substitution models detected seven species, a result not fully congruent with our morphological species hypothesis. These methods lumped *Hamat*. *cordivulva* **sp. nov.** and *Hamat*. *foveata* as a single species, whereas *T*. *parva* **sp. nov.** was split into two. GMYC and bPTP yielded slightly different results (9–10 species). Contrary to the results of distance-based methods, GMYC and bPTP not only discriminated *Hamat*. *cordivulva* **sp. nov.** and *Hamat*. *foveata*, but further divided *Hamat*. *foveata* into two lineages. However, the bPTP support for *H*. *cordivulva* **sp. nov.** and two lineages of *H*. *foveata* were relatively low (< 0.7). Notably, GMYC with a coalescent constant population model further split *T*. *auriola* **sp. nov.** into two lineages. Similarly, topology-based methods also classified *T*. *parva* **sp. nov.** as a distinct species (Fig 3).

For BPP analyses of *COI* and *H3* data set, the model 4–6 (considering *T*. *parva* **sp. nov.** as containing two species) had weak support values (PP < 0.9) in all prior settings (Table 1). The model 1–3 in all prior settings mostly have strong support values (PP > 0.95). However, when *Hamat*. *foveata* was split (model 3) into two species, the posterior probability of prior settings with larger population size parameter were relatively weak (Table 1). To sum up, BPP results suggested that *T*. *parva* **sp. nov.** is a distinct species corresponding to the morphological species, and *H*. *cordivulva* **sp. nov.** and *H*. *foveata* are two independent species, although lumping them together as one species also have high support.

### Phylogeny

The *COI* dataset contains 63 unique sequences from 24 taxa (20 ingroups and four outgroups), of which 57 sequences were newly generated in this study. The total length of the dataset was 659 base pairs, including 234 variable sites and 205 parsimony-informative sites.

**Fig 4:**
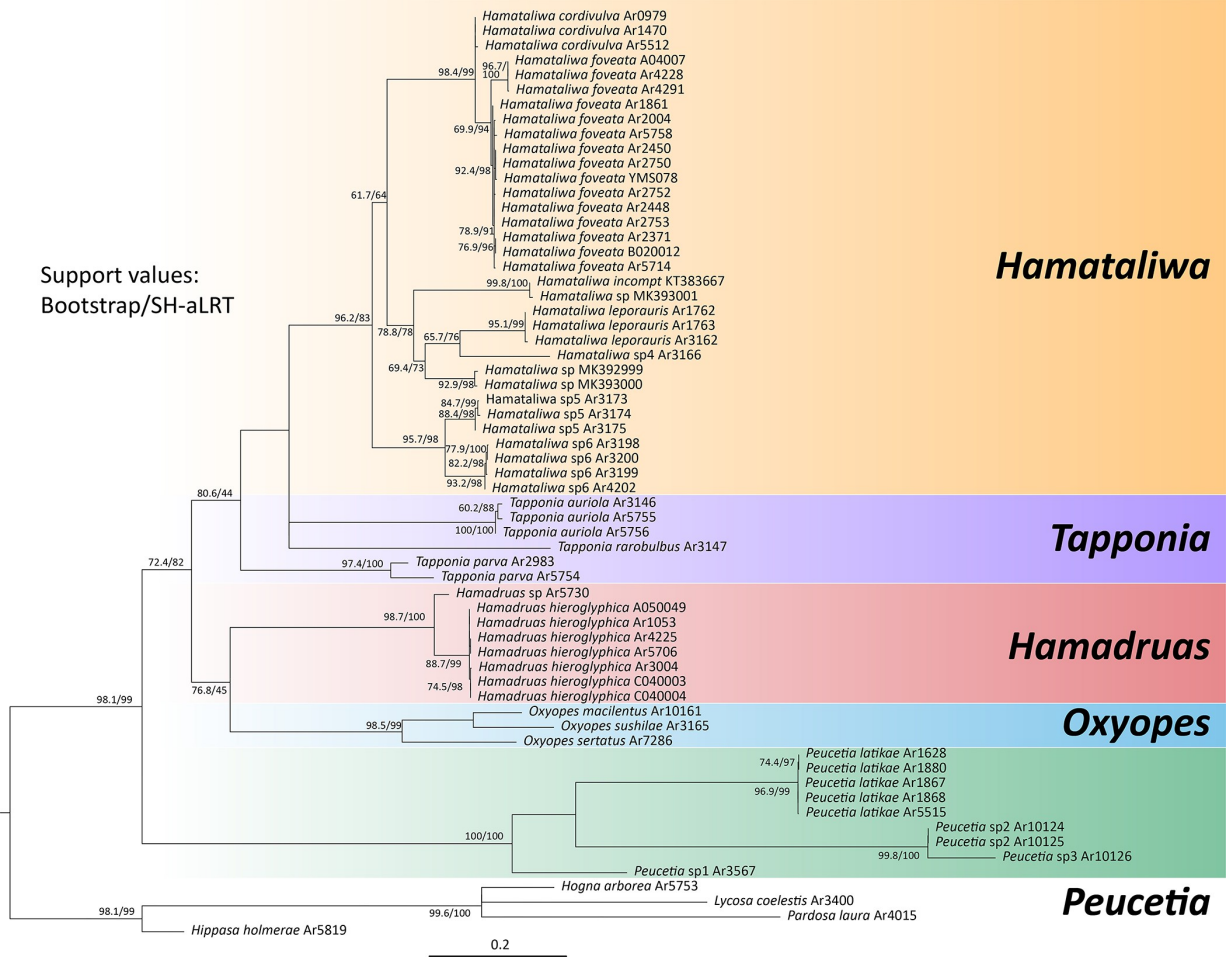
Maximum likelihood phylogenetic tree of Oxyopidae based on *COI* dataset. Bootstrap values are represented by dots, and support levels are presented in different colors.

Both topologies of maximum likelihood (Fig 4) and Bayesian inference (S1 Fig) phylogenetic trees were similar, except for the relationship of *Tapponia*. *Hamadruas* and *Peucetia*, were two well-supported clades (BS = 100; PP = 1), and *Hamataliwa* was also monophyletic, but the support value was relatively low in ML tree (BS = 49; PP = 0.99). *Tapponia* in ML tree formed a monophyly with low support (BS = 13), and was a sister clade to *Hamataliwa*, while *T*. *auriola* **sp. nov.** and *T*. *rarobulbus* **sp. nov.** were sister to *Hamataliwa*, and *T*. *parva* **sp. nov.** was sister to the above clade in BI tree (S1 Fig). The supports for a monophyletic *Tapponia* were low in the two trees. Surprisingly, *Hamadruas*, which was similar to *Hamataliwa* in genital organs, habitat preference and egg guarding behavior, was most closely related to *Oxyopes*. The two genera grouped into a clade with low branch support (BS = 38; PP = 0.54). Moreover, the monophyly of all eight candidate morphological species in the present study from Taiwan were well-supported (BS > 90; PP > 0.9) excepted for *H*. *cordivulva* **sp. nov.** in the BI tree (PP = 0.6) and *T*. *rarobulbus* **sp. nov.** which had only one sample.

## Discussion

### Species delimitation for Taiwanese *Hamadruas*, *Hamataliwa* and *Tapponia*

The present study identified eight morphological oxyopid species from Taiwan, including five new species and three newly recorded species, of which *Hamadruas*, *Hamataliwa* and *Tapponia* are newly recorded genera. Nevertheless, a two-step approach molecular species delimitation of these three genera based on *COI* and *H3* data revealed between seven and ten species by different methods, and none of them fully confirmed the morphological species. The main inconsistencies occurred in two clades: (1) *Hamat*. *cordivulva* **sp. nov.** + *Hamat*. *foveata*, and (2) *Tapponia parva* **sp. nov.**

ABGD and ASAP are genetic distance-based methods that automatically assigns individuals and aim to find the sequence divergence threshold (barcoding gap) at which interspecific divergence exceeds intraspecific variation. However, the genetic gap between interspecific and intraspecific divergence varies across taxonomic lineages, and the barcoding overlap increases with closely related taxa or recent speciation groups [53–55]. Therefore, the results of species delimitation are largely affected by the evolutionary history of the target groups. For datasets with a mixture of shallow and deep divergence lineages, species may be underestimated (false-negative) due to relatively large barcoding gap [56,57]. In contrast to distance-based methods, GMYC and bPTP are topology-based methods that require an estimated gene tree as input data. Factors influencing the performance of GMYC involves the effective population size and divergence times between species [58,59], phylogenetic methods, taxon level and sampling coverage [53,60,61], and migration rates among populations [62]. Some earlier studies have shown that the performance of these methods is primarily affected by the ratio of population sizes to species divergence times [63], and the two methods often over-split (false-positive) species [48,56,64,65].

Our distance-based and topology-based delimitation methods do indeed produce different results. ABGD and ASAP lumped *Hamat*. *cordivulva* **sp. nov.** and *Hamat*. *foveata* as a single species, as was expected due to their relatively low interspecific distance (1.88%, Table 2). However, intraspecific distances for the two species (0.1% and 0.95%) are smaller than interspecific distance, suggesting that a barcoding gap still exists to some degree. In contrast, GMYC and bPTP distinguished *Hamat*. *cordivulva* **sp. nov.** and *Hamat*. *foveata* and divided the latter into two separate species. We found that *Hamat*. *foveata* from Taiwan contains two distinct clades (Fig 3). Although these two clades have an overlapping distribution, the genetic variation within each clade (0.03% and 0.2%) are lower than the genetic divergence between them (1.83%). The epigyne of *H*. *foveata* shows morphological variation, such as in the width of rim, but the variation does not correspond to a pattern of geographic distribution. To sum up, the results suggest that the number of the lynx species in Taiwan was underestimated by genetic-based methods and overestimated by topology-based methods.

The species limits for *T*. *parva* **sp. nov.** is another conflict between molecular and morphological data. We did not detect the distinguishable morphological difference of two examined samples, whereas both genetic-based and topology-based methods split *T*. *parva* **sp. nov.** into two separate species (Fig 3). The genetic distance within the species is relatively large (4.77%), which is slight greater than the generally accepted threshold for the interspecific genetic distance in spiders, birds, fishes, reptiles, and insects (2–4%) [31,66–71]. Nevertheless, this threshold value is challenged by some cases in which a distinct barcoding gap is not present [72,73], or the threshold is notably higher [74]. In addition, even though the large genetic variance of *T*. *parva* **sp. nov.**, it still less than interspecific divergence with other species (9.20–15.87%, Table 2), which implied a barcoding gap exist [69,75,76]. In addition, we have only examined two male specimens from different localities (Fig 2), and the female is still unknown. Species rarity is one of the challenges for species delimitation, and insufficient specimen sampling could lead to different outcomes [56]. Therefore, given the limitation of our sample size and *COI* sequences data, it is challenging to distinguish deep population structure or different species [77], and an advanced evaluation through multispecies coalescent approach is necessary.

Bayesian coalescent-based methods are statistical frameworks that accounts for the uncertainty (as posterior probability) in species delimitation [22]. Unlike ABGD, GMYC and bPTP, which are designed for analyzing single locus data, BPP generally requires multi-locus data, though the method is often used in single-locus studies [78]. Previous studies showed that BPP yields high probability for correct species delimitation, and is less influenced by unequal population size and unbalanced sampling [79–81]. However, it is still a challenge for BPP to recognize recently diverged species [63,82]. In the present study, *COI* and *H3* were used in BPP analysis, and the results showed all models that split *T*. *parva* **sp. nov.** (model 4–6) have a lower posterior probability (PP = 0.57–0.89). This result was different from that of other molecular species delimitation, but was consistent with morphological evidence. The best models in the BPP outcomes are model 1 (three species) and 2 (two species), both have high support (PP > 0.99) regardless of prior combinations. The further split of *Hamat*. *foveata*, like as in GMYC and bPTP, was not fully supported by BPP. Earlier simulation studies argued that the multi-species coalescent approaches tend to overestimate species number because they actually delimit population structure of species rather than speciation events [79,83]. However, the BPP results from this study present a more conservative outcome when compared with other methods, failing to further split *Hamat*. *foveata* and *T*. *parva* **sp. nov.** into different species.

Molecular species delimitation methods and morphological evidence often yield different results [13,28,84–86]. The definition of a species is a continuing debate due to different species concepts and the continuous nature of speciation [27]. During the speciation process, various species properties, including morphological traits, monophyly in phylogeny, differentiated genetic components or other mechanisms for reproductive isolation are accumulated between divergent lineages until they have completely separated, resulting in the formation of independent species. The relative rates of character change in lineage divergence process are heterogeneous, so we may not be able to detect all the properties [24,27]. There are scenarios, for example, that result in morphological divergence faster than genetic differentiation [87–90], including selection pressure, recently diverged time or ongoing gene flow. Hence, morphological species can be recognized, but the species cannot be defined genetically or phylogenetically. Although it is generally accepted that no standard guideline exists for determining how many criteria are sufficient for species designation, and that it should be context-dependent, to decide the criteria remains a challenging task. In some instances, species are defined based on specific evidence (whether morphological, genetic, or ecological data), even though not all available evidence supports the classification [24,91,92]. For example, Domènech et al. [92] described a new species, *Theridion promisuum* Domènech & Crespo, 2020, based on the diagnostic morphological traits and *ITS2* genetic data, which was in contrast to *COI* data that suggested genetic mixture across members of the *T*. *melanurum* group. Similarly, Huber & Dimitrov [91] regards the two closely related spider, *Paiwana chengpoi* (Huber & Dimitrov, 2014) and *P*. *pingtung* (Huber & Dimitrov, 2014), as two independent species according to microhabitat differentiation and phenotype difference (such as body coloration and body proportions); however, their genitalia and *COI* sequences were almost indistinguishable. In contrast, some cases suggested highly genetic differentiated subpopulations as a single species due to the absence of congruent evidence, even though subpopulations were recognized as separate species by molecular species delimitation [24,93,94]. For example, Salgado-Roa et al. [93] detected two genetic clusters of *Ancylometes bogotensis* (Keyserling, 1877) separated by Eastern Cordillera. Both mPTP and BPP analysis suggested that these two lineages could constitute distinct species. However, they considered the *A*. *bogotensis* as genetically differentiated subpopulations due to the lack of behavioral, morphological, and reproductive isolation evidence. Similarly, Salgado-Roa [94] also regarded five geographical clusters of *Gasteracantha cancriformis* (Linnaeus, 1758) from Andes mountains, Caribbean, and Galapagos Islands as a single species because they did not found morphological differences in male genitalia among populations, although a barcoding gap was detected and most molecular species delimitation supported five distinct species.

This study documented that the species boundaries in lynx spiders are not always identical when evaluated under different lines of evidence. All methods have their strengths and limitations, and there is no an universal criterion applicable to taxa with variable evolutionary histories [95–97]. If we use the most stringent criteria, it will inevitably lead to doubtful conclusions, such as lumping *Hamataliwa cordivulva* **sp. nov.** and *H*. *foveata*, whereas their genitalia are conspicuously divergent in morphology. In this study, we considered *H*. *cordivulva* **sp. nov.**, *H*. *foveata*, and *Tapponia parva* **sp. nov.** as three separate species based on three species concepts: morphological (distinct morphological traits compared to other groups), genetic (lower intraspecific divergence compared to interspecific divergence), and phylogenetic (a monophyletic group with common ancestry). Furthermore, integrative taxonomy should be incorporated into biodiversity conservation and monitoring. In this context, diagnostic characters are critical, as they aid in operational purpose and taxonomic stability (and avoid taxonomic inflation) [98–100]. Finally, we supposed that our species hypothesis is reasonable, and should be further validated with advanced data such as genome-wide and ecological data [16,101].

This study supports the integrative approaches rather than “minimalist taxonomy” [100,102,103], which rapidly estimate the species number of hyper-diverse taxa by using only DNA barcoding information without detailed specimen examination, morphological diagnosis, and multiple species delimitation methods [104]. While there is an urgent need to quickly identify and document biodiversity using molecular approach, morphologically diagnosable characters remain important and practical for species identification. The present study agrees with this argument as species with shallow genetic divergent can exhibit distinct morphological difference, and the same dataset could result in inconsistent taxonomic assessment through different analysis methods (Fig 3). In conclusion, the application of single species delimitation methods, especially relying on only one locus, could inaccurately determine the species number, potentially leading to either over- or under-estimation. This can result in taxonomic instability and the development of incorrectly informed conservation strategies [105].

### Phylogeny of Oxyopidae

Although the monophyly of the Oxyopidae has rarely been disputed in spider phylogenies [106–108], the generic and species relationships within Oxyopidae remain equivocal. An earlier study examined the shape and distribution of cuticular scales from 82 species of Oxyopids, and indicated that the presence of three distinct groups: (1) *Tapinillus*, *Peucetia* and *Schaenicoscelis*; (2) *Hamataliwa* and *Tapponia*; (3) *Oxyopes* and *Hostus* [109]. However, the molecular validation for phylogenetic relationships of Oxyopids is still lacking.

We investigated the phylogenetic relationship of Oxyopidae using *COI* gene as part of this study. In both ML and BI topologies, the genera *Hamadruas*, *Hamataliwa*, *Oxyopes* and *Peucetia* formed monophyletic groups with high support values (BS > 90 in ML and > 0.9 in BI), which supported our genus assignment of new species. Nevertheless, the status of *Tapponia* is uncertain (Fig 4). We found that *Tapponia* was not a monophyletic group, with *T*. *auriola* **sp. nov.** and *T*. *rarobulbus* **sp. nov.** as sister group to *Hamataliwa*, and *T*. *parva* **sp. nov.** as sister to aforementioned clade in both ML and BI phylogenetic trees with low node supported values. *Tapponia* is currently a monotypic genus, as Deeleman-Reinhold (2009) transferred all species hitherto included in the genus to *Hamadruas* and *Hamataliwa*, except for the type-species *T*. *micans* Simon, 1885 [110]. Nevertheless, among the species of *Tapponia* in this study, only *T*. *auriola* **sp. nov.** undoubtedly conform to the characters of the genus according to morphological characters, including male palp with a short patellar apophysis, and carapace and abdomen with iridescent scales [7]. *T*. *parva* **sp. nov.** and *T*. *rarobulbus* **sp. nov.**, however, lack these characters. Instead, their wider ocular region, longer MOA-L, and a relatively short clypeus are consistent with the description of *Tapponia* by Simon [110]. Obviously, the relationship and definition of *Hamataliwa* and *Tapponia* need to be reclarified. For example, we found that *Hamat*. *wangi* Lin & Li, 2022 [111] possesses a patellar apophysis on male palp but lacks iridescent scales ([111]: figs 30–31). Because of deficiency of comprehensive taxonomic review and limited information available of this mysterious group, we temporality treated them as *Tapponia*.

*Hamadruas*, which had been separated from *Hamataliwa* [7], was found herein most closely related to *Oxyopes* with relatively low supported values (BS = 73 and 47 in ML and BI tree, respectively). Consequently, the phylogeny pattern was inconsistent with that proposed by Townsend and Felgenhauer [109]. According to the male palp morphology (with tegular lobe and cymbium basal apophysis), habitat preference (canopy or understory layer) and egg sac guarding behavior (egg sac is attached on a leaf hanged on silk), we expected that *Hamadruas*, *Hamataliwa* and *Tapponia* would be closely related. However, due to the limited use of the *COI* gene in the phylogenetic analysis, multi-locus data should be employed in further analysis of Oxyopidae phylogeny.

## Taxonomy

### Genus *Hamadruas* Deeleman-Reinhold, 2009

#### Diagnosis

**Fig 5:**
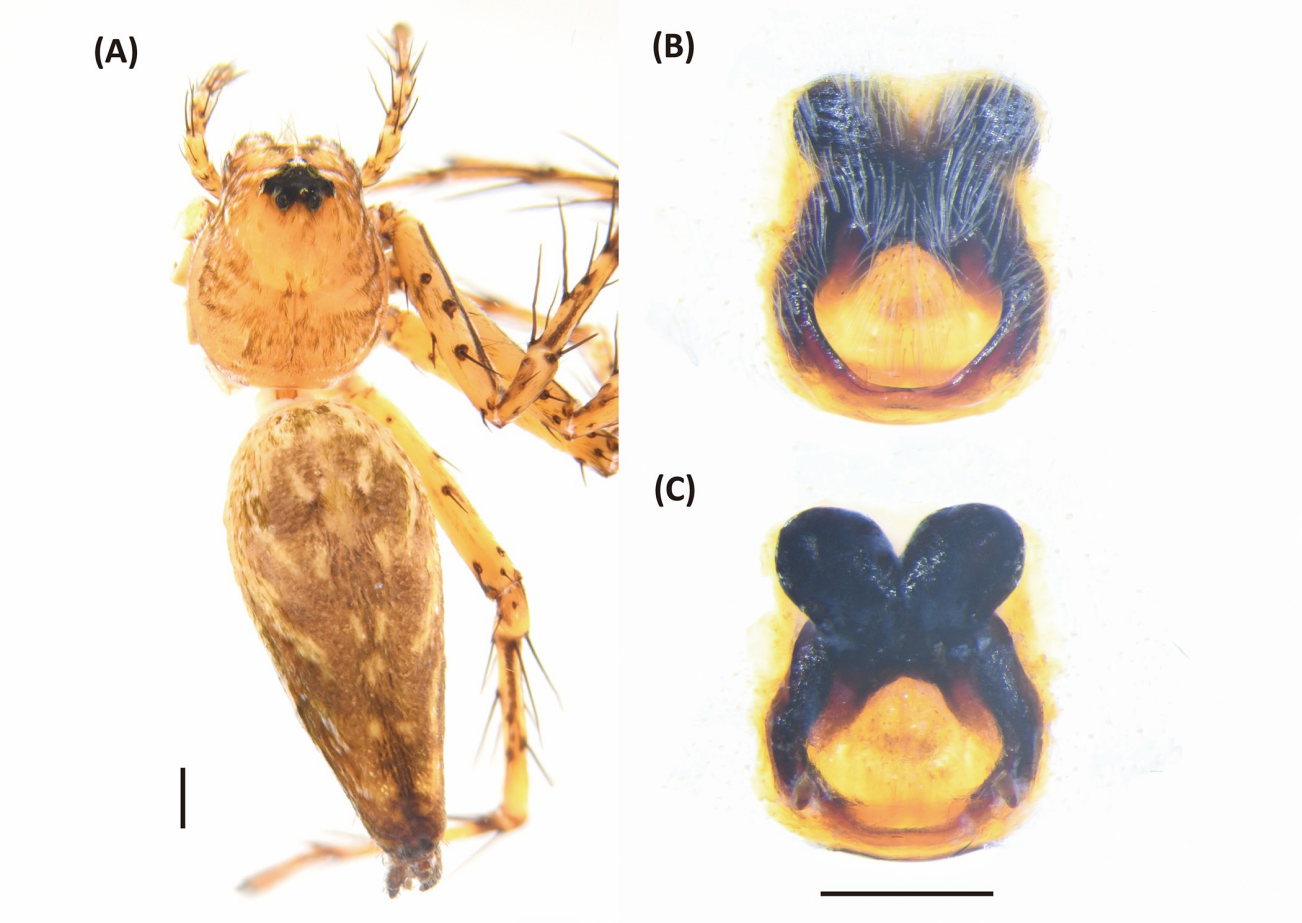
*Hamadruas hieroglyphica*, female. A, Habitus, dorsal view; B, Epigyne; C, Vulva. Scale bar: A = 1 mm; B–C = 0.5 mm.

**Fig 6:**
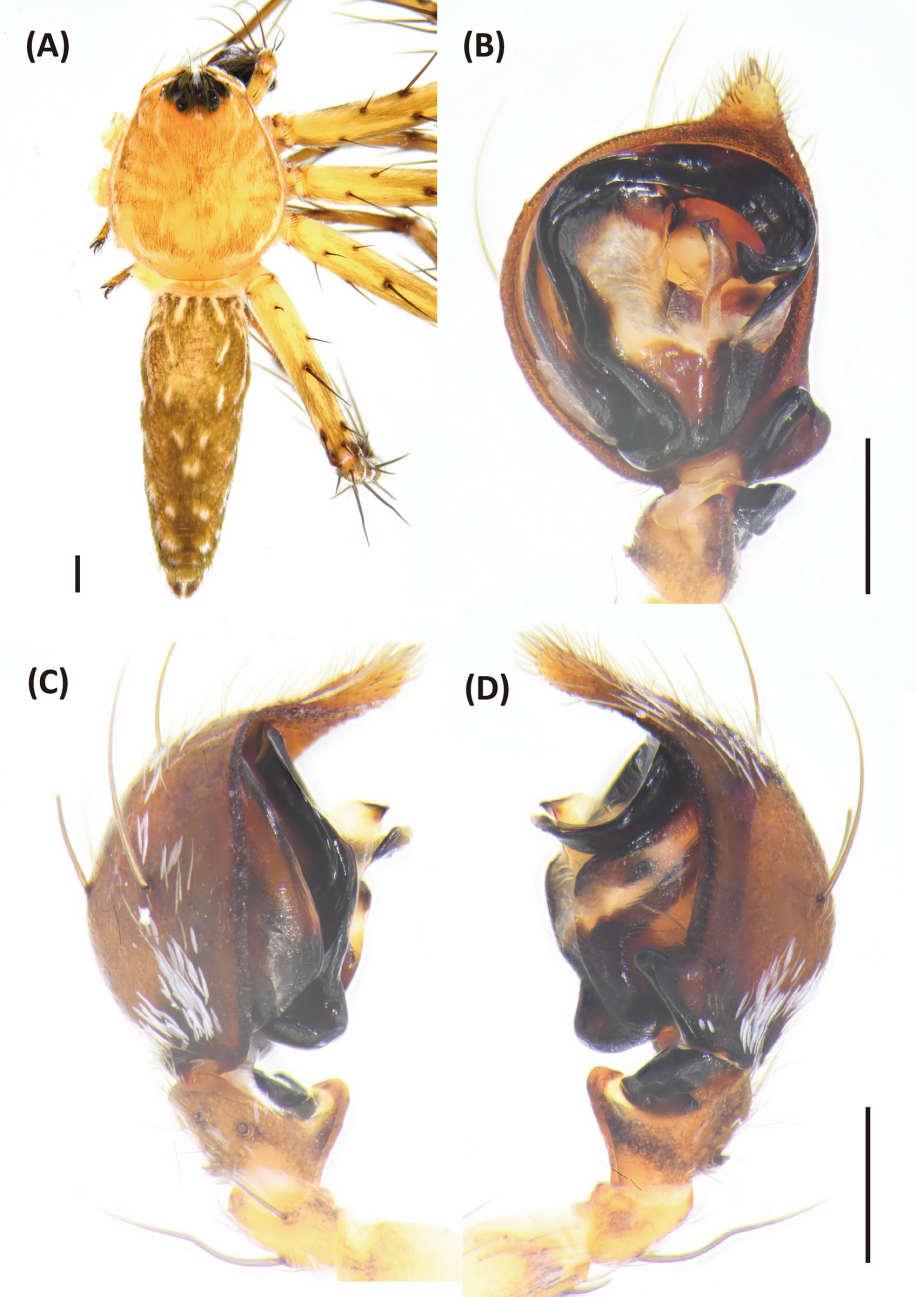
*Hamadruas hieroglyphica*, male. A, Habitus, dorsal view; B–D, Palp (B, ventral view; C, prolateral view; D, retrolateral view). Scale bars: A = 1 mm; B–D = 0.5 mm.

*Hamadruas* is similar to *Hamataliwa* and *Tapponia* in male palp with a basal apophysis on cymbium and with a tegular lobe, but can be distinguished from latter by following characters: (1) carapace height relatively lower, and usually saddle-shaped dorsally in lateral view; (2) front and rear face sloping (vertical in *Hamataliwa* and *Tapponia*); (3) abdomen 1.5–2.0 times longer than carapace (Figs 5A and 6A; abdomen does not exceed 1.2 times the length of carapace in *Hamataliwa* and *Tapponia*); (4) tegular lobe looping with a pit (Fig 6B; the pit not present in *Hamataliwa* and *Tapponia*) [7].

### *Hamadruas hieroglyphica* (Thorell, 1887)

*Oxyopes hieroglyphicus* Thorell 1887: 332 [112].

*Tapponia hieroglyphica* Thorell 1895: 254 [113].

*Hamadruas hieroglyphica* Deeleman-Reinhold 2009: figs 57–63 [7]; Tang & Li 2012: fig 1A–1C, 2A–2B [8]; Sen & Sudhin 2023: fig 2A–2E [114].

Figs 5 and 6, S2A and S2B Fig

### Material examined. TAIWAN

***Taipei City*:** Tianliao (25.0971 N, 121.5909 E; 170m elevation), one female (TESRI-Ar2746), 15 Oct. 2016, Han-Po Chang leg.; Zhishanyan (25.1039 N, 121.5302 E; 45m elevation), one male (TESRI-Ar1401), 02 Jun. 2015, Chi-Cheng Li leg.; Fuyang National Park (25.0160 N, 121.5548 E; 80m elevation), two females (TESRI-Ar1053–1054), 28 Oct. 2014, Ying-Yuan Lo leg., two females and two males (TESRI-Ar1454/1461/1480/1493), 15 Aug. 2015, Kuang-Ping Yu leg. ***New Taipei City*:** Changxing Road (24.9398 N, 121.5542 E; 100m elevation), two females (TESRI-A050003/A050049), 29 Aug. 2015, Da-Qing Chang leg. ***Taoyuan City*:** Cihu Memorial Sculpture Park (24.8415 N, 121.2936 E; 225m elevation), one male (TESRI-Ar5724), 14 Sep. 2019, Kuang-Ping Yu leg. ***Miaoli County*:** Bogongkeng (24.379000 N, 120.739600 E; 440m elevation), one female (TESRI-Ar5530), 19 Oct. 2019, Kuang-Ping Yu leg. ***Taichung City*:** Dakeng (24.1739 N, 120.7819 E; 430m elevation), one female (TESRI-Ar3164), 23 Jul. 2019, Ren-Chung Cheng leg.; Dakeng Earthquake Park (24.1783 N, 120.7340 E; 170m elevation), one female (TESRI-Ar5533), 21 Apr. 2020, Chi-Chun Liao leg.; Xing-Long Community (24.1151 N, 120.7348 E; 105m elevation), one female (TESRI-Ar5583), 22 Jun. 2020, Chi-Chun Liao leg.; Bo-Ai Elementary School (24.2018 N, 121.0056 E; 765m elevation), one male (TESRI-Ar5508), 12 Jun. 2020, Chi-Chun Liao leg. ***Changhua County*:** Kengneikeng Forest Trail (23.8286 N, 120.6145 E; 140m elevation), one male and four females (TESRI-Ar5595–5598), 21 Sep. 2019, Chang-Lin Chung leg. ***Nantou County*:** Jiufenershan (23.9443 N, 120.8493 E; 640m elevation), one female (TESRI-Ar2161), 21 Sep. 2016, Chong-Sheng Huang leg.; Xiaping Tropical Botanical Garden (23.7737 N, 120.6727 E; 140m elevation), one female (TESRI-Ar2747), 27 Oct. 2017, Kuang-Ping Yu leg.; Zhongliao (23.8997 N, 120.7621 E; 170m elevation), one male (TESRI-CX086), 26 Jul. 2018, Ying-Yuan Lo leg.; Endemic Species Research Institute (23.8293 N, 120.8014 E; 250m elevation), one male (TESRI-Ar2528), 11 Sep. 2017, Chong-Sheng Huang leg.; Zhenguo Temple (23.8478 N, 120.8022 E; 350m elevation), five females (TESRI-Ar2796–2800), 04 Oct. 2018, Ying-Yuan Lo leg. ***Chiayi County*:** Chukou Nature Center (23.4531 N, 120.5595 E; 165m elevation), one male (TESRI-B030008), 21 Aug. 2015, and one female (TESRI-B031703011), 12 Aug. 2017, Chen-Yao Lin leg. ***Kaohsiung City*:** Shiba Luohan Mountain (22.9488 N, 120.6415 E; 205m elevation), one female (TESRI-Ar3004), 05 Oct. 2018, Ying-Yuan Lo leg.; Duona Forest Trail (22.8880 N, 120.7379 E; 1050m elevation), three males and one female (TESRI-C040003–C040005/C040032), 06 Sep. 2015, Yu-Da Lai leg. ***Yilan County*:** Nanao (24.4347 N, 121.7783 E; 75m elevation), one female (TESRI-Ar5941), 01 Sep. 2020, Chi Wei leg. ***Hualian County*:** Heren (24.2481 N, 121.7163 E, 35m elevation), 12 males (TESRI-Ar5989–5990), 31 Aug. 2020, and three females (TESRI-Ar4419–4421), 5 Nov. 2020, Chi Wei leg.; Dekalun Trail (24.1593 N, 121.6217 E; 300m elevation), one female (TESRI-Ar2741), 26 Oct. 2017, Chong-Sheng Huang leg., one female (TESRI-Ar5592), 25 Oct. 2019, Chang-Lin Chuang leg.; Tongmen (23.9646 N, 121.4991 E; 160m elevation), one female (TESRI-Ar1902), 31 Aug. 2016, Ying-Yuan Lo leg.; Liyu (Carp) Lake (23.9221 N, 121.5078 E; 160m elevation), one female (TESRI-Ar2742), 23 Oct. 2017, Chong-Sheng Huang leg., two females (TESRI-Ar1878–1879), 31 Aug. 2016, Ying-Yuan Lo leg.; Luoshan Waterfall (23.1812 N, 121.2955 E; 510m elevation), one male (TESRI-D01-0015), 27 Aug. 2015, Wen-Chun Huang leg. ***Taitung County*:** Tianlong Ancient Trail (23.173662 N, 121.042300 E; 700m elevation), one female (TESRI-Ar5534), 04 Jan. 2019, Wen-Chun Huang leg.; Lijia Forest Trail (22.8031 N, 121.0508 E; 360m elevation), two males (TESRI-Ar1436–1437), 06 Aug. 2015, Ren-Chung Cheng leg.; Jhihben Forest Trail (22.7043 N, 121.0145 E; 255m elevatiom), one male (TESRI-Ar2735), 20 Sep. 2017, Ying-Yuan Lo leg; Jhihben National Forest Recreation Area (22.6882 N, 120.9911 E; 170m elevation), one male (TESRI-Ar3541), 10 Aug. 2019, Ying-Yuan Lo leg. **CHINA.** Aberdeem, one female (TESRI-Ar4225), 27 Aug. 2018, one male and one female (TESRI-Ar5706–5707), 03 Sep. 2019, Ho Yin Yip leg.

#### Diagnosis

This species is similar to *Hamadruas severa* (Thorell, 1895) in female epigyne, but can be distinguished from latter by the following characters: (1)

larger body size (total length13–15 mm in female and 7–8 mm in male, whereas *H*. *severa* 9–12 mm in female and 7–8 mm in male; [113]); (2) MA with square and straight distal rim (Fig 6B; tip with slightly concave edge in *H*. *severa*); (3) different color pattern [7].

#### Description

Female (TESRI-Ar2797). Total length 13.1; carapace length 4.6, width 3.7; abdomen length 8.5, width 4.0. Carapace yellowish brown, with black ocular region and dense radial stripes. Radial furrows indistinct and fovea longitudinal. In dorsal view, eyes arranged in four rows with AER strongly recurved and PER strongly procurved. Eye diameters and inter-distances: AME 0.12, ALE 0.30, PME 0.22, PLE 0.22, eye sizes ALE > PME = PLE > AME; MOA-AW 0.40, MOA-PW 0.80, MOA-L 1.10; AME-I 0.16, PME-I 0.36, AML-I 0.06, PML-I 0.28; clypeus height 0.84. Chelicerae yellowish, downward with two promarginal teeth (one large and one small) and one retromarginal tooth. Endite and labium longer than wide. Sternum yellowish, with few setae. Legs yellowish, clothed with many conspicuous long spines, femur I–II with two black stripes, femur III with one stripe and one discontinuous stripe, and femur IV with one stripe in ventral side. Three claws. Pedipalps bear apical claw. Measurements of pedipalps and legs: pedipalp 4.3 (1.3, 0.6, 0.9, 1.5), leg I 17.6 (4.7, 1.5, 5.0, 4.8, 1.6), leg II 16.9 (4.9, 1.5, 4.5, 4.5, 1.5), leg III 15.1 (4.6, 1.5, 3.8, 4.0, 1.2), leg IV 15.6 (4.8, 1.5, 3.8, 4.4, 1.1). Leg formula: I > II > IV > III. Abdomen long oval, yellowish brown, cardiac mark indistinct, scattered with dark spots and lines, and five to six pair of conspicuous pale spots (Fig 5A). Lateral region yellowish brown with several pale stripes. In ventral view, a pair of black longitudinal bands forms V-shaped from epigastric furrow extend to spinnerets. Spinnerets black.

Epigyne with a large central depression, and the sclerotized circle-shaped posterior edge (Fig 5B). Spermathecae round, visible by transparency. Copulatory ducts curved, fertilization ducts long and slender (Fig 5C).

Male (TESRI-Ar2528). Body shape and color pattern similar to that of female, but the cymbium and tibia of pedipalps black, the white spots on dorsal carapace and abdomen brighter, and dorsal abdomen dark green (Fig 6A). Total length 7.6; carapace length 3.3, width 2.7; abdomen length 4.3, width 1.7. Eye diameters and inter-distances: AME 0.10, ALE 0.24, PME 0.18, PLE 0.18, eye sizes ALE > PME = PLE > AME; MOA-L 0.94, MOA-AW 0.30, MOA-PW 0.66; AME-I 0.10, PME-I 0.30, AML-I 0.06, PML-I 0.24; clypeus height 0.64. Measurements of pedipalps and legs: pedipalp 3.2 (1.0, 0.4, 0.4, 1.4), leg I 14.5 (3.7, 1.1, 4.0, 4.1, 1.6), leg II 13.5 (3.6, 1.1, 3.6, 3.8, 1.4), leg III 11.9 (3.3, 1.1, 3.0, 3.4, 1.1), leg IV 12.4 (3.5, 1.0, 3.0, 3.7, 1.2). Leg formula: I > II > IV > III.

Palp with a column-shaped ventral retrolateral tibia apophysis in ventral view, and a larger and chunk-shaped dorsal retrolateral tibial apophysis. Basal outgrowth of cymbium with a furrow, the tip triangular in lateral view. Tegular lobe strongly curved. Median apophysis with the membranous base and the black, blunt tip with a distal triangular process in lateral view. Embolus twist and relatively wide. Conductor thick, distal part sickle-like (Fig 6B–6D.

#### Distribution

China, Myanmar, and Taiwan (newly recorded)

#### Remarks

Thorell [112] first described the female of *Oxyopes hieroglyphica* from Burma (Myanmar), while the male was unknown. Deeleman-Reinhold [7] examined specimens from Palon and Tonghoo (Myanmar) that were deposited in MCSNG (Museo Civico di Storia Naturale Giacomo Doria, Italy), and first described the male. Although we cannot examine the holotype, the body size and morphology of male palp organ of the specimens from Taiwan were congruent with those described by Deeleman-Reinhold [7].

In addition, *Hamadruas sikkimensis*, which was first described by Tikader [115] in 1970, is similar to *H*. *hieroglyphica*. However, the genital organs of *H*. *sikkimensis* were not described in original report. Although the illustration of female epigyne appears slightly different from that of *H*. *hieroglyphica*, the diagnostic characters remain unrecognized (Tikader [115]: fig. 47B–47C). Several later reports of *H*. *sikkimensis* from China (Hu et al. [116]: fig 1A–1D, but wrong label as fig 1A–1C in original report; Hu & Zhang [117]: figs 1–4; Zhang et al. [118]: figs 21–25) were more similar to *H*. *hieroglyphica* rather than *H*. *sikkimensis* as illustrated by Tikader [115]. For example, Deeleman-Reinhold [7] pointed out the description of genital organs and ventral pattern of *H*. *sikkimensis* in Zhang et al. [118] does not agree with those of Tikader and Biswas [119] (pl. VIII, fig. 108–109, which was same as Tikader [115]). Duo to the unavailability of *H*. *sikkimensis* type from India, we supposed these specimens might be misidentified and further review of *Hamadruas* is needed.

### Genus *Hamataliwa* Keyserling, 1887

#### Diagnosis

**Fig 7:**
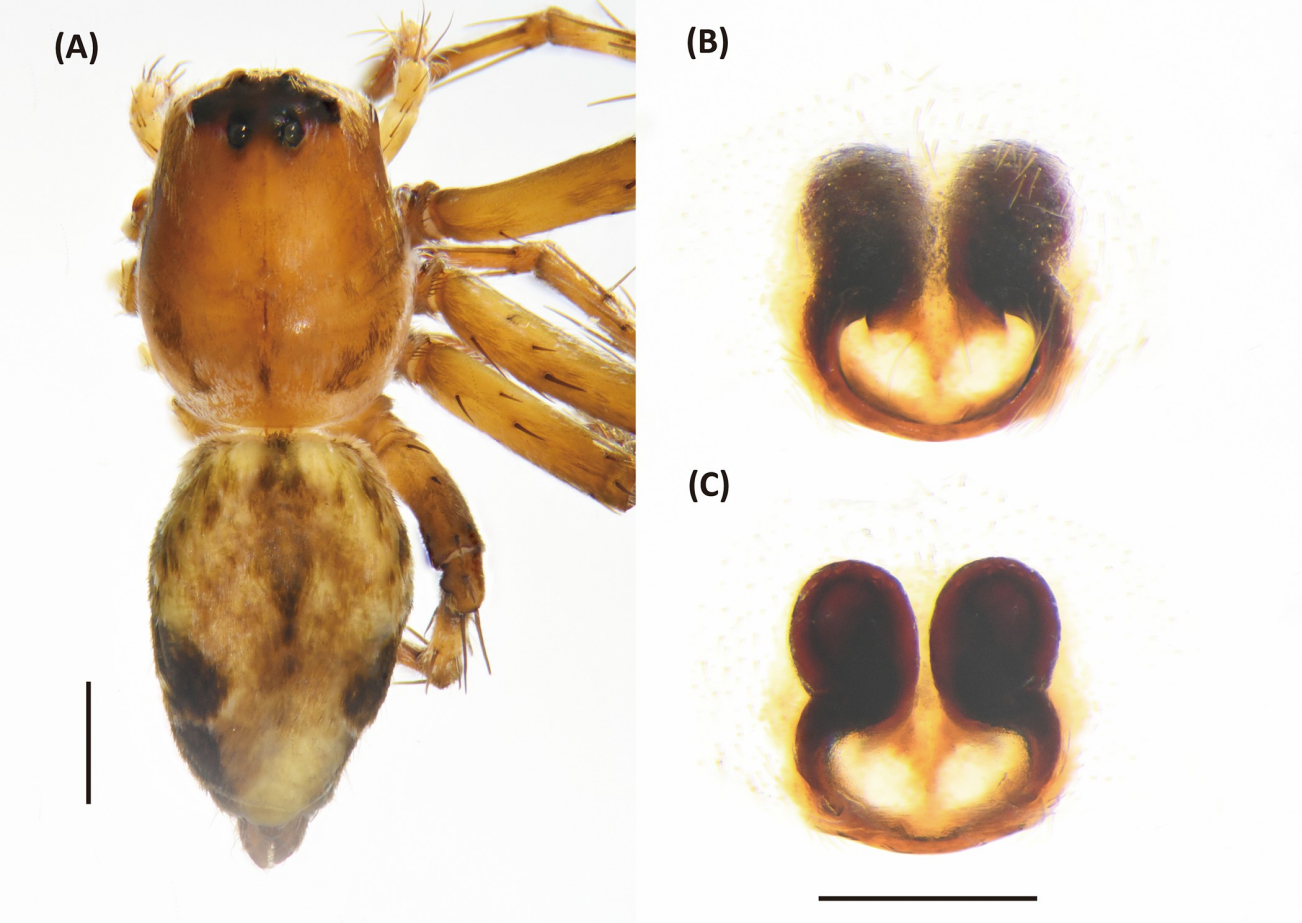
*Hamataliwa cordivulva* sp. nov., female. A, Habitus, dorsal view; B, Epigyne; C, Vulva. Scale bar: A = 1 mm; B–C = 0.5 mm.

*Hamataliwa* is similar to *Hamadruas* and *Tapponia* in male palp with a basal apophysis on cymbium and with a tegular lobe, but can be distinguished from latter by following characters: (1) carapace relatively higher, and female head region usually square (Figs 7A, 11A and 13A); (2) front and rear face vertical and sometimes receding; (3) tegular lobe sharply curved without pit (Figs 9A, 12A and 15A; with a pit in *Hamadruas*) [7].

#### *Hamataliwa cordivulva* sp. Nov

**Fig 8:**
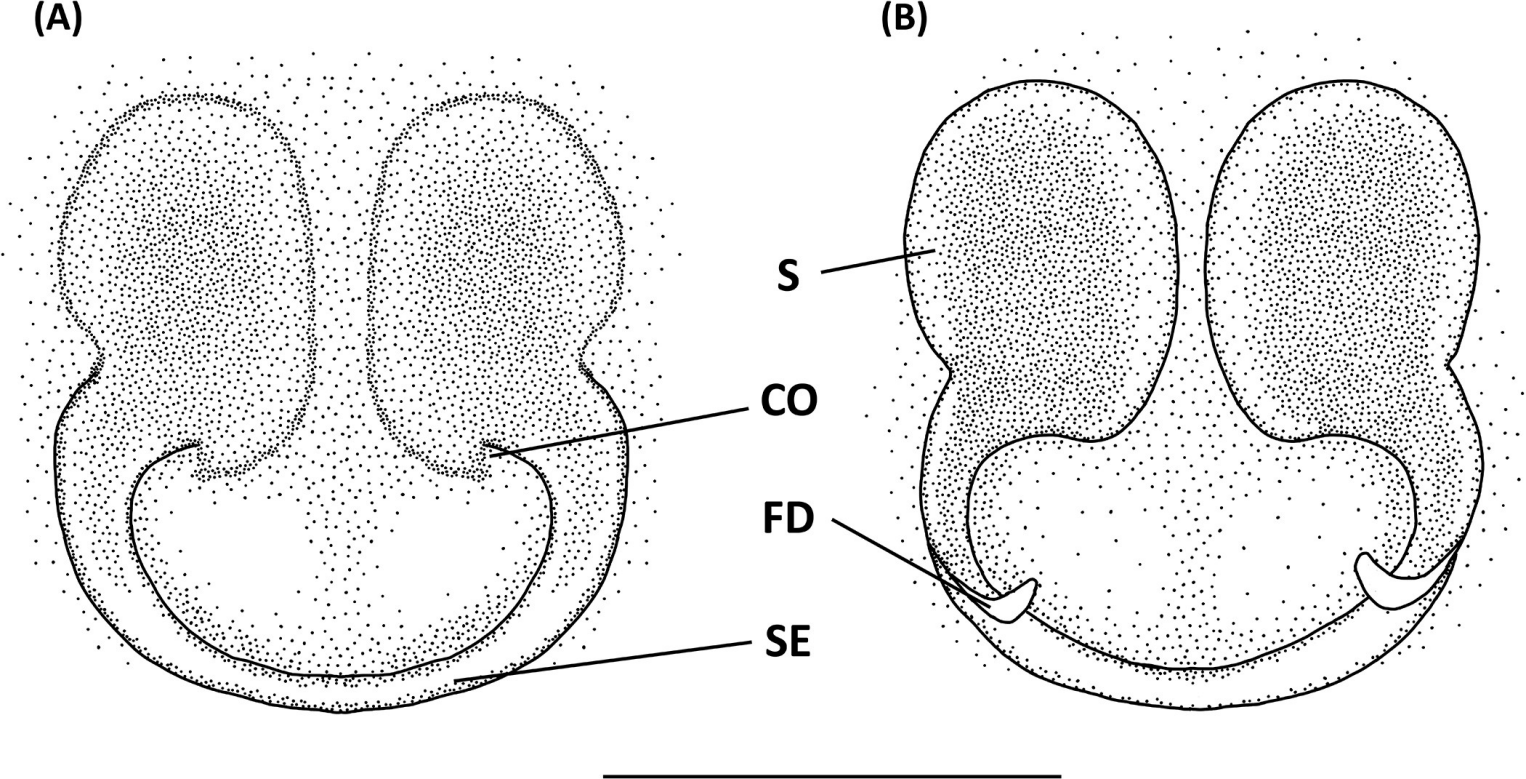
Epigyne and vulva of *Hamataliwa cordivulva* sp. nov., female. A, Epigyne; B, Vulva. Scale bar = 0.5 mm.

**Fig 9:**
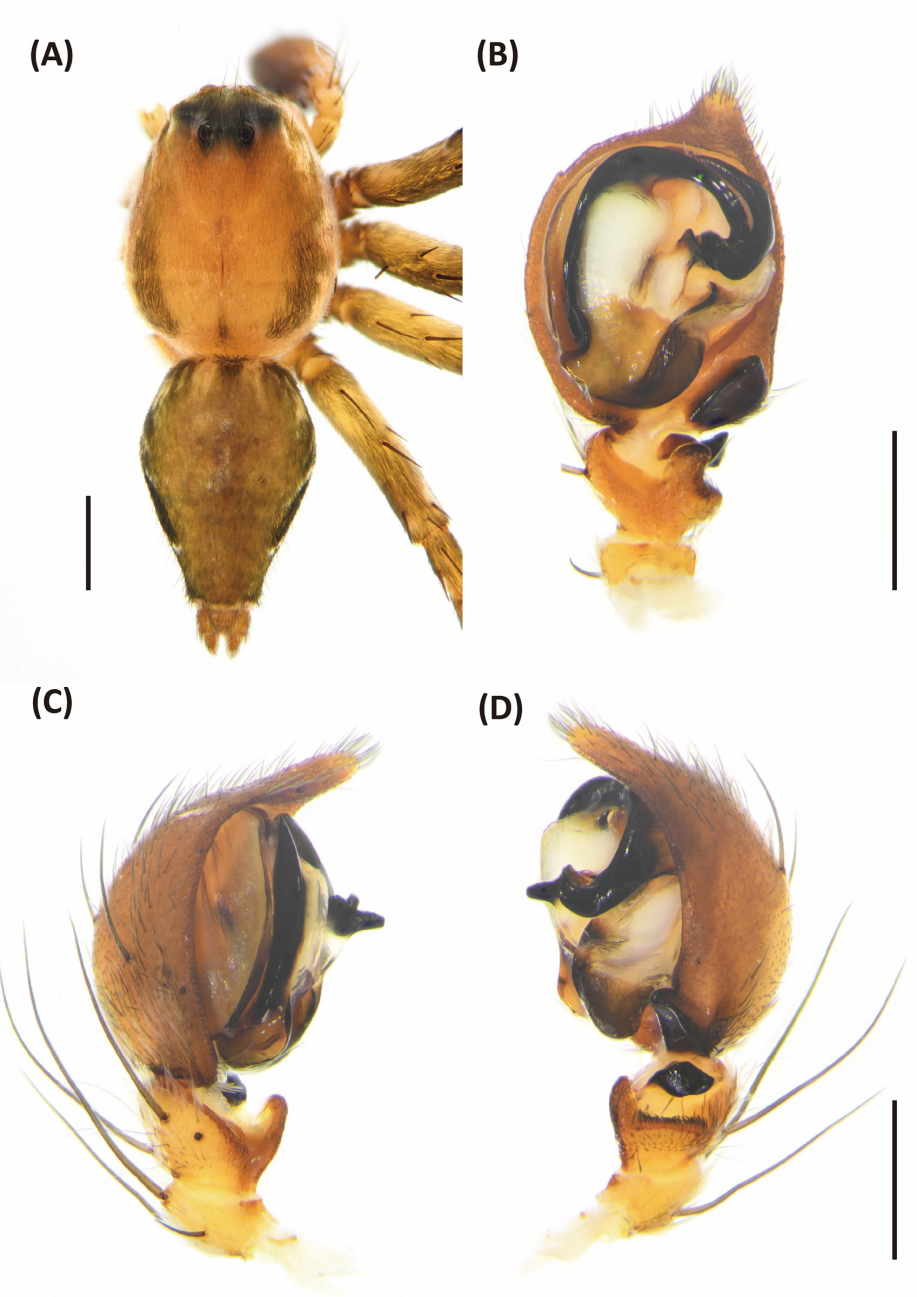
*Hamataliwa cordivulva* sp. nov., male. A, Habitus, dorsal view; B–D, Palp (B, ventral view; C, prolateral view; D, retrolateral view). Scale bar: A = 1 mm; B–D = 0.5 mm.

**Fig 10:**
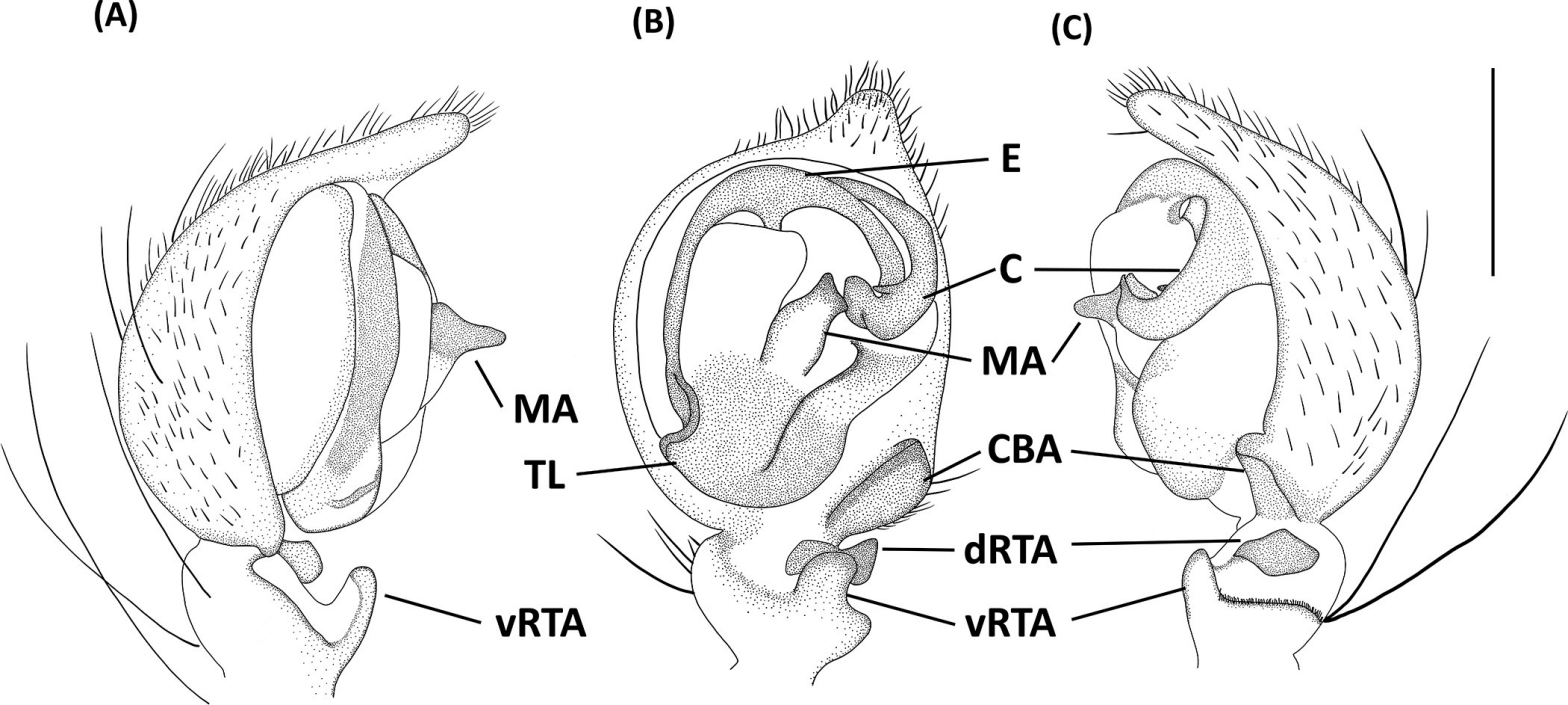
Left palp of *Hamataliwa cordivulva* sp. nov., male. A, prolateral view; B, ventral view; C, retrolateral view. Scale bar = 0.5 mm.

Figs 7–10, S2C and S2D Fig

#### Type material. Holotype

**TAIWAN. *Hualien County*:** One male (TESRI-Ar4271), 04 May 2020, Heren, (24.2481 N, 121.7163 E, 35m elevation), Chi-Chun Liao leg. **Paratype**. ***Taichung City*:** One female (TESRI-Ar0979), 17 Aug. 2014, one female (TESRI-Ar5512), 28 May 2020, Wushikeng Experimental Station (24.2744 N, 120.9482 E; 990 m elevation), Ying-Yuan Lo leg.

**Other material examined. *Taichung City*:** sampling location same as paratype, one female (TESRI-Ar1470), 13 Aug. 2015, Ying-Yuan Lo leg., one female (TESRI-Ar5535), 16 Jul. 2020, Kuang-Ping Yu leg., one male (TESRI-Ar4280), 28 May 2020, Ying-Yuan Lo leg.

#### Diagnosis

This species is similar to *H*. *foveata* in male palp organ with a cavity-shaped retrolateral depression in tibia, but can be distinguished from latter by the following characters: (1) the vRTA is larger; (2) the edge of tibial retrolateral depression present horizontal section (Figs 9D and 10C); (3) the tip of MA is protruded ventrally; (4) the central depression of epigyne is larger and wider (Figs 7B and 8A).

#### Description

Female (TESRI-Ar0979, Paratype). Total length 6.5; carapace length 3.1, width 2.4; abdomen length 3.4, width 2.3. Carapace yellowish, with black eye region and a pair of brown lines on lateral margins. Radial furrows indistinct and fovea longitudinal. In dorsal view, eyes arranged in four rows with AER strongly recurved and PER strongly procurved. Eye diameters and inter-distances: AME 0.10, ALE 0.24, PME 0.20, PLE 0.20, eye sizes ALE > PME = PLE > AME; MOA-L 0.98, MOA-AW 0.32, MOA-PW 0.66; AME-I 0.14, PME-I 0.30, AML-I 0.08, PML-I 0.3; clypeus height 0.44. Chelicerae yellowish, downward with two promarginal teeth and one retromarginal tooth. Endite and labium longer than wide. Sternum yellowish, heart-like. Abdomen long oval with a grey cardiac mark, lateral region dark. In ventral view, a broad dark longitudinal band extend from epigastric furrow to spinnerets. Legs yellowish, clothed with many conspicuous long spines. Three claws. Pedipalps bear apical claw. Measurements of pedipalps and legs: pedipalp 2.9 (0.8, 0.5, 0.6, 1.0), leg I 11.2 (3.7, 0.9, 3.1, 2.4, 1.1), leg II 9.8 (3.1, 0.9, 2.6, 2.2, 1.0), leg III 7.5 (2.2, 1.0, 1.8, 1.7, 0.8), leg IV 7.7 (2.4, 0.9, 1.7, 1.9, 0.8). Leg formula: I > II > III ≒ IV.

Epigynewith a large, wide, and heart-shaped central depression, and the sclerotized semicircle posterior edge (Figs 7A and 8A). Spermathecae large and elliptical. Fertilization ducts slender with hook-like terminal (Figs 7B and 8B).

Male (TESRI-Ar4271, Holotype). Body shape and color pattern similar to that of female. Total length 5.7; carapace length 3.1, width 2.5; abdomen length 2.6, width 1.8. Eye diameters and inter-distances: AME 0.10, ALE 0.24, PME 0.18, PLE 0.18, eye sizes ALE > PME = PLE > AME; MOA-L 0.94, MOA-AW 0.32, MOA-PW 0.64; AME-I 0.14, PME-I 0.30, AML-I 0.10, PML-I 0.36; clypeus height 0.44. Measurements of pedipalps and legs: pedipalp 2.6 (0.7, 0.3, 0.3, 1.3), leg I 11.3 (3.5, 0.9, 3.1, 2.5, 1.3), leg II 10.1 (3.0, 0.9, 2.7, 2.3, 1.2), leg III 7.6 (2.3, 0.8, 1.8, 1.9, 0.8), leg IV 7.7 (2.3, 0.8, 1.7, 2.1, 0.8). Leg formula: I > II > III ≒ IV.

Palpal tibia with two apophyses: ventral retrolateral tibia apophysis thumb-like, connected with a truncated ridge which bear a row of coarse setae, and dorsal retrolateral tibial apophysis black, larger and lump-shaped. Cymbium with a basal auricular outgrowth. Median apophysis with the membranous base and the black, blunt tip with a distal triangular process point toward ventral side. Embolus relatively thick. Conductor wide, distal part sickle-like (Figs 9B–9D and 10A–10C).

#### Distribution

Endemic to Taiwan.

#### Etymology

The specific epithet ‘*cordivulva*’ is a noun from the combination of the Latin *cor*, Genitive case *cordis* (heart) and *vulva*, and refers to the heart-like depression of epigyne.

#### *Hamataliwa foveata* Tang & Li, 2012

*Hamataliwa foveata* Tang & Li 2012: figs 3A–D, 4A–D, 5A–D [8].

**Fig 11:**
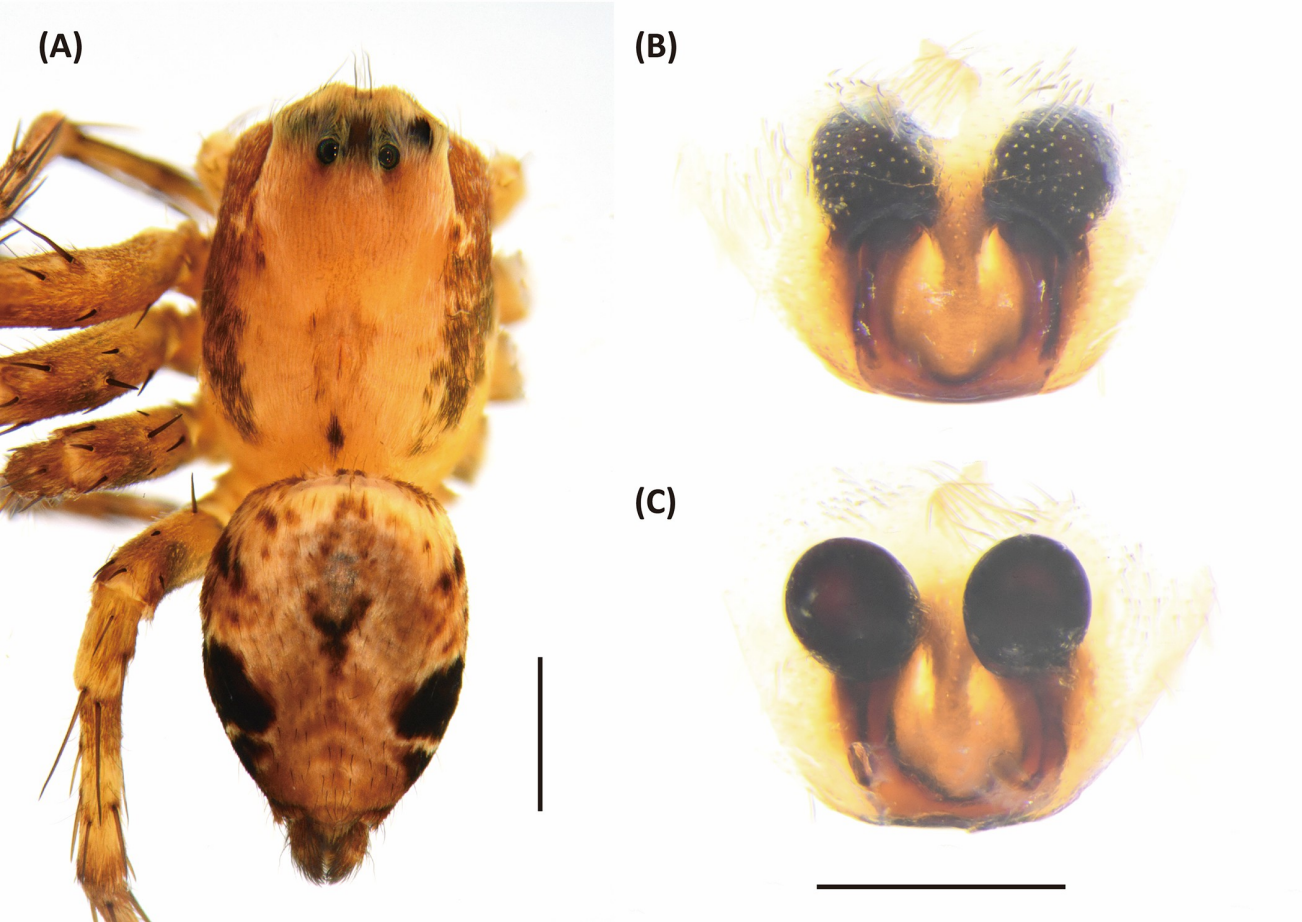
*Hamataliwa foveata*, female. A, Habitus, dorsal view; B, Epigyne; C, Vulva. Scale bar: A = 1 mm; B–C = 0.5 mm.

**Fig 12:**
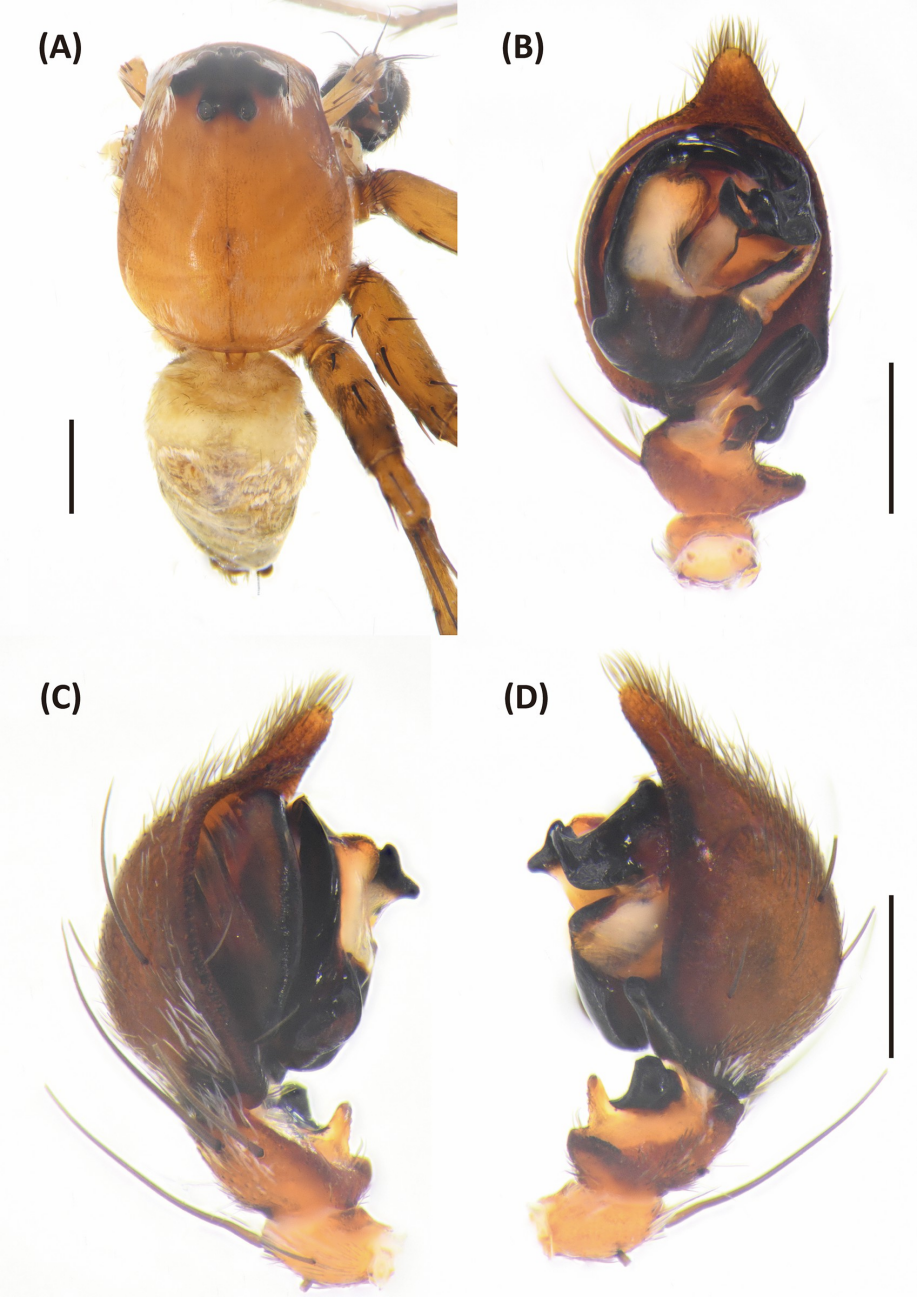
*Hamataliwa foveata*, male. A, Habitus, dorsal view; B–D, Palp (B, ventral view; C, prolateral view; D, retrolateral view). Scale bar: A = 1 mm; B–D = 0.5 mm.

Figs 11 and 12, S2E and S2F Fig

#### Material examined. TAIWAN

***Taipei City*:** Pamier Park (25.1211 N, 121.5935 E; 350m elevation), two females (TESRI-YMS021/YMS062), 30 Jun. 2017, one female (TESRI-YMS078), 10 Sep. 2017, Li-Jing Huang leg.; Fuyang Eco Park (25.0160 N, 121.5548 E; 80m elevation), one female (TESRI-Ar0317), 20 Jul. 2013, Jia-Wen Ke leg., one female (TESRI-A040007), 15 Aug. 2015, one female (TESRI-Ar2752), 26 Jun. 2017, Kuang-Ping Yu leg. ***New Taipei City*:** Huliaotan (24.9485 N, 121.7398 E; 220m elevation), one male (TESRI-Ar3153), 9 Jul. 2018, Chi-Cheng Li leg. ***Hsinchu County*:** Shuilian Bridge Trail (24.6558 N, 121.0254 E; 150m elevation), one female (TESRI-Ar2750), 13 Jun. 2017, Zhi-Wen Hsu leg.; Shaotanwo (24.7404 N, 121.1115 E; 155m elevation), one female (TESRI-Ar2448), 2 Aug. 2017, Yeh-Wei Chou leg.; Dashanbei (24.7005 N, 121.1561 E; 250m elevation), one female (TESRI-Ar2450), 20 Aug. 2017, Yeh-Wei Chou leg. ***Miaoli County*:** Bogongkeng (24.3790 N, 120.7396 E; 440m elevation), two females (TESRI-Ar5712–5713), 4 Jul. 2019, Kuan-Ping Yu leg., one female (TESRI-Ar4222), 25 Jul. 2019, Ho Yin Yip leg. ***Taichung City*:** Dakeng (24.1739 N, 120.7819 E; 430 m elevation), one female (TESRI-Ar5714), Jul 2011, one female (TESRI-Ar4228), Sep. 2018, Ren-Chung Cheng leg.; Tong-Lin Ecological Park (24.0482 N, 120.7842 E; 335m elevation), two males (TESRI-Ar4270/Ar4272), 15 Jan. 2020, Ying-Yuan Lo leg. ***Nantou County*:** Jiufenershan (23.949080 N, 120.849745 E, 600m elevation), one male (TESR-Ar2004), 28 Jun. 2016, one female (TESRI-Ar2371), 13 Jun. 2017, Ying-Yuan Lo leg.; Zhongliao (23.8664 N, 120.7831 E, 200 m elevation), one male (TESRI-CX368), one female (TESRI-CX346), 19 Jun. 2019, Thiao-Tien Chang leg.; Lianhuachi (23.9184 N, 120.8845 E; 690m elevation), one female (TESRI-Ar5715), Jul. 2011, Ren-Chung Cheng leg., one female (TESRI-Ar0316), 20 Jun. 2013, Yu-Jen Tsao leg., two females (TESRI-Ar4290–4291), 19 Feb. 2020, Chi-Chun Liao leg. ***Changhua County*:** Kengneikeng Forest Trail (23.8291 N, 120.6166 E; 230m elevation), one female (TESRI-Ar5593), 21 Sep. 2019, Chang-Lin Chung leg. ***Chiayi County*:** Lantan Trail (23.4769 N, 120.4952 E; 90m elevation), one female (TESRI-B020012), 12 Aug. 2015, De-Lun Wu leg. ***Kaohsiung City*:** Longmu Community (22.6906 N, 120.4031 E; 50m elevation), one male (TESRI-Ar2753), 29 May 2018, Guo-Yuan Wu leg. ***Taitung County*:** Yanping forest road (22.8989 N, 121.0653 E; 335m elevation), one male (TESRI-Ar1861), 18 Jun. 2016, Ying-Yuan Lo leg.; Jialuoban (22.3622 N, 120.8702 E; 110m elevation), one male (TESRI-Ar5758), 10 Apr. 2021, Ying-Yuan Lo leg.

#### Diagnosis

This species is similar to *H*. *cordivulva* **sp. nov.** in male palp organ, with a cavity-shaped retrolateral depression, but can be distinguished from latter by the following characters: (1) the vRTA is smaller; (2) the edge of tibial retrolateral depression protruded laterally (Fig 12B and 12D); (3) the tip of MA is not protruded ventrally; (4) the central depression of epigyne is smaller and narrower.

#### Description

Female (TESRI-Ar2371). Total length 5.8; carapace length 3.0, width 2.3; abdomen length 2.8, width 2.1. Carapace yellowish, with dark eye region and a pair of brown lines on lateral margins. Radial furrows indistinct and fovea longitudinal. In dorsal view, eyes arranged in four rows with AER strongly recurved and PER strongly procurved. Eye diameters and inter-distances: AME 0.08, ALE 0.26, PME 0.18, PLE 0.18, eye sizes ALE > PME = PLE > AME; MOA-L 1.00, MOA-AW 0.32, MOA-PW 0.66; AME-I 0.16, PME-I 0.30, AML-I 0.10, PML-I 0.44; clypeus height 0.54. Chelicerae yellowish, downward with two promarginal teeth and one retromarginal tooth. Endite and labium longer than wide. Sternum yellowish, heart-like. Abdomen oval, yellowish brown, with a pair of dark patches on two-third of the abdomen on ventral view (Fig 11A). Lateral region dark. In ventral view, a broad dark longitudinal band extend from epigastric furrow to spinnerets. *Legs* yellowish, clothed with many conspicuous long spines. Three claws. Pedipalps bear apical claw. Measurements of pedipalps and legs: pedipalp 2.8 (0.8, 0.4, 0.6, 1.0), leg I 10.6 (3.5, 1.0, 2.9, 2.3, 0.9), leg II 10.0 (3.1, 1.0, 2.6, 2.2, 1.1), leg III 7.2 (2.3, 0.9, 1.6, 1.7, 0.7), leg IV 7.5 (2.4, 0.9, 1.5, 1.8, 0.9). Leg formula: I > II > IV > III.

Epigynal posterior sclerotized edge thick, U-shaped, with distinct central depression. Spermathecae round, and fertilization ducts thin and twisted (Fig 11B and 11C).

Male (TESRI-Ar2004). Body shape and color pattern similar to that of female. Total length 6.4; carapace length 3.4, width 2.6; abdomen length 3.0, width 1.8. Eye diameters and inter-distances: AME 0.08, ALE 0.24, PME 0.18, PLE 0.18, eye sizes ALE > PME = PLE > AME; MOA-L 1.00, MOA-AW 0.30, MOA-PW 0.64; AME-I 0.14, PME-I 0.28, AML-I 0.10, PML-I 0.40; clypeus height 0.50. Measurements of pedipalps and legs: pedipalp 3.0 (1.0, 0.3, 0.4, 1.3), leg I 11.8 (3.2, 0.9, 3.3, 2.9, 1.5), leg II 10.9 (3.2, 0.9, 3.0, 2.6, 1.2), leg III 8.4 (2.5, 0.9, 2.0, 2.1, 0.9), leg IV 8.5 (2.5, 1.0, 1.8, 2.3, 0.9). Leg formula: I > II > IV > III.

Palpal tibia with two apophyses: ventral retrolateral tibia apophysis finger-like, join an extended protrude and form a laterally depression; dorsal retrolateral tibial apophysis laminate-shaped and twisted. Cymbium with a basal auricular outgrowth. Median apophysis with the membranous base, the distal part wide, and the tip is protruded ventrally. Embolus relatively thick. Conductor wide, distal part hook-shaped (Fig 12B–12D).

#### Distribution

China and Taiwan (newly recorded).

#### Remarks

The appearance and morphological characters of male palp organ of *Hamataliwa foveata* from Taiwan are consistent with the description and illustration of specimens from Yunnan, China in Tang and Li [8]. However, the epigyne characters are slightly different. The copulatory ducts of Yunnan specimens twisted near spermathecae, but the copulatory ducts of Taiwan specimens do not curve distinctly near spermathecae. In addition, the shape and curvature of copulatory ducts of *H*. *foveata* from Taiwan are more or less variable among different populations.

#### *Hamataliwa leporauris* sp. Nov

Figs 13–16.

**Fig 13:**
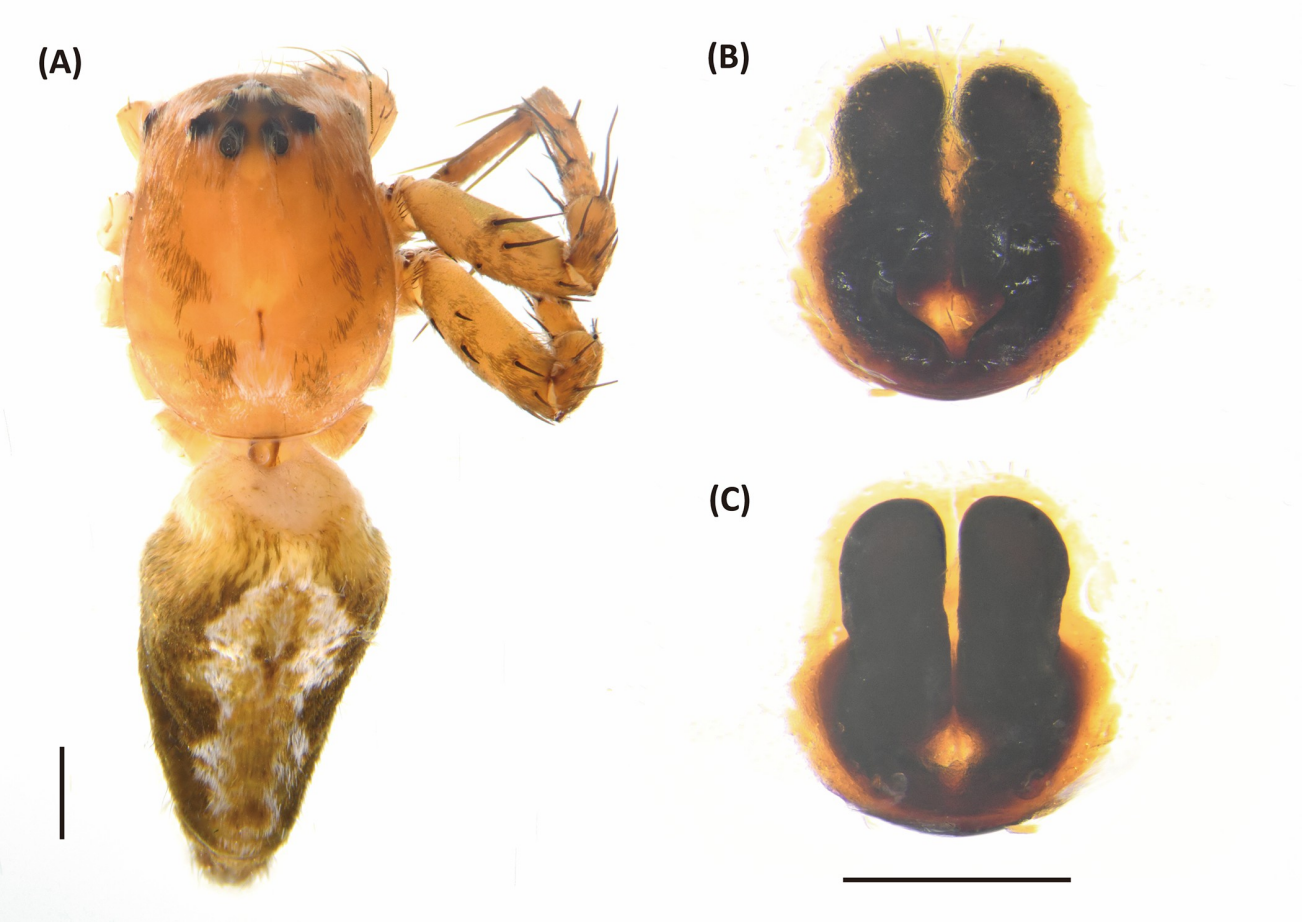
*Hamataliwa leporauris* sp. nov., female. A, Habitus, dorsal view; B, Epigyne; C, Vulva. Scale bar: A = 1 mm; B–C = 0.5 mm.

**Fig 14:**
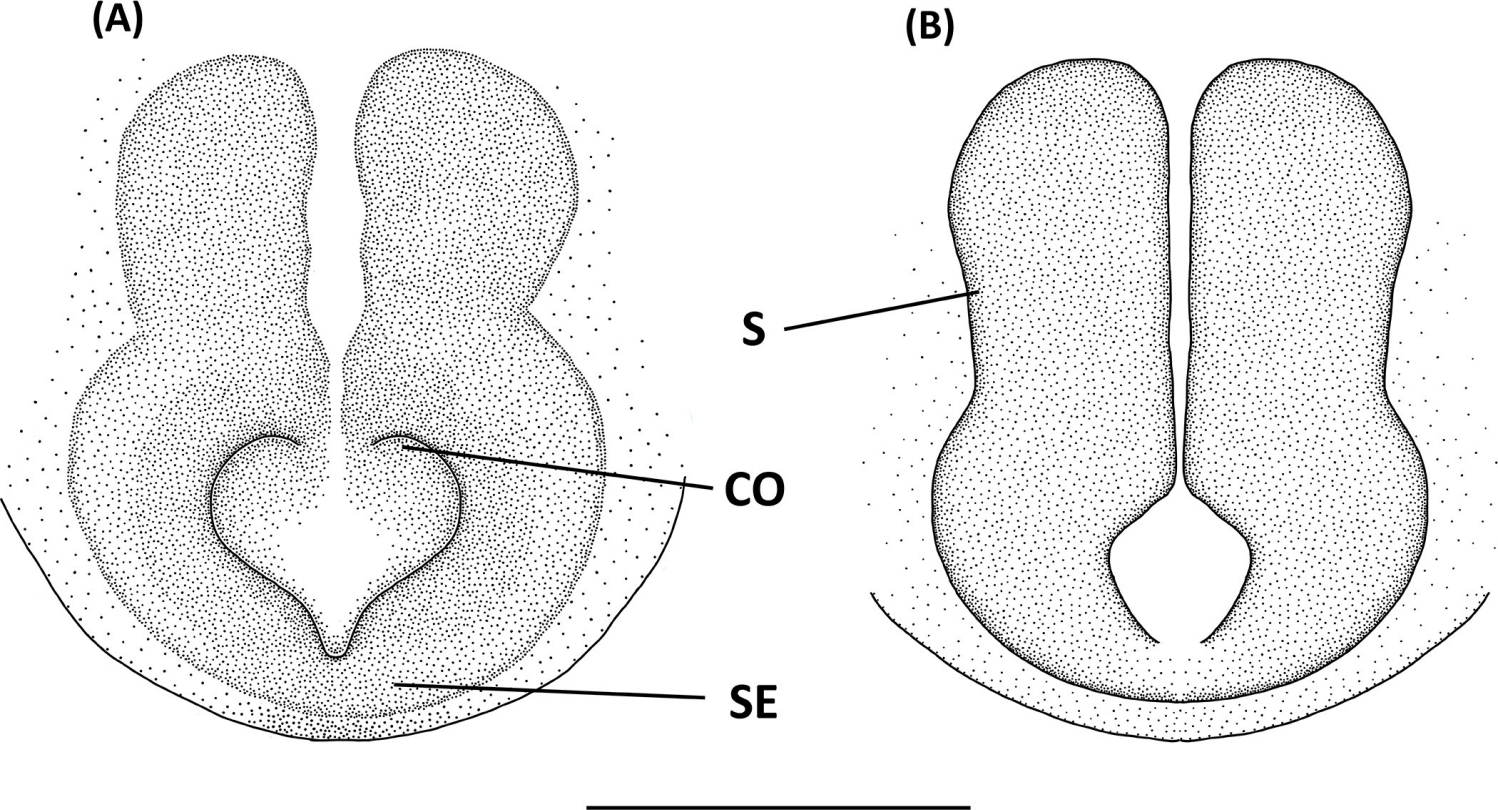
Epigyne and vulva of *Hamataliwa leporauris* sp. nov., female. A, Epigyne; B, Vulva. Scale bar = 0.5 mm.

**Fig 15:**
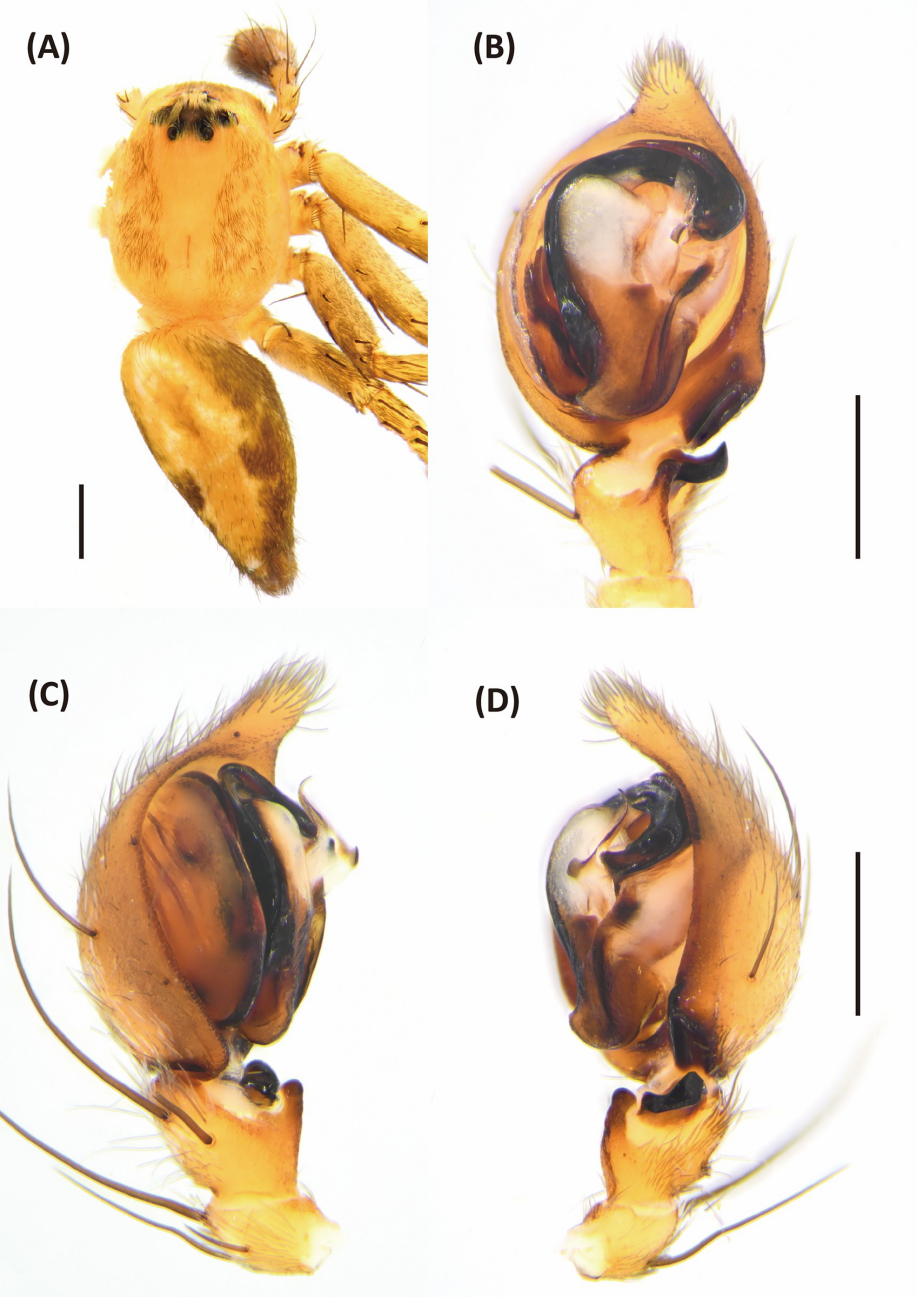
*Hamataliwa leporauris* sp. nov., male. A, Habitus, dorsal view; B–D, Palp (B, ventral view; C, prolateral view; D, retrolateral view). Scale bar: A = 1 mm; B–D = 0.5 mm.

**Fig 16:**
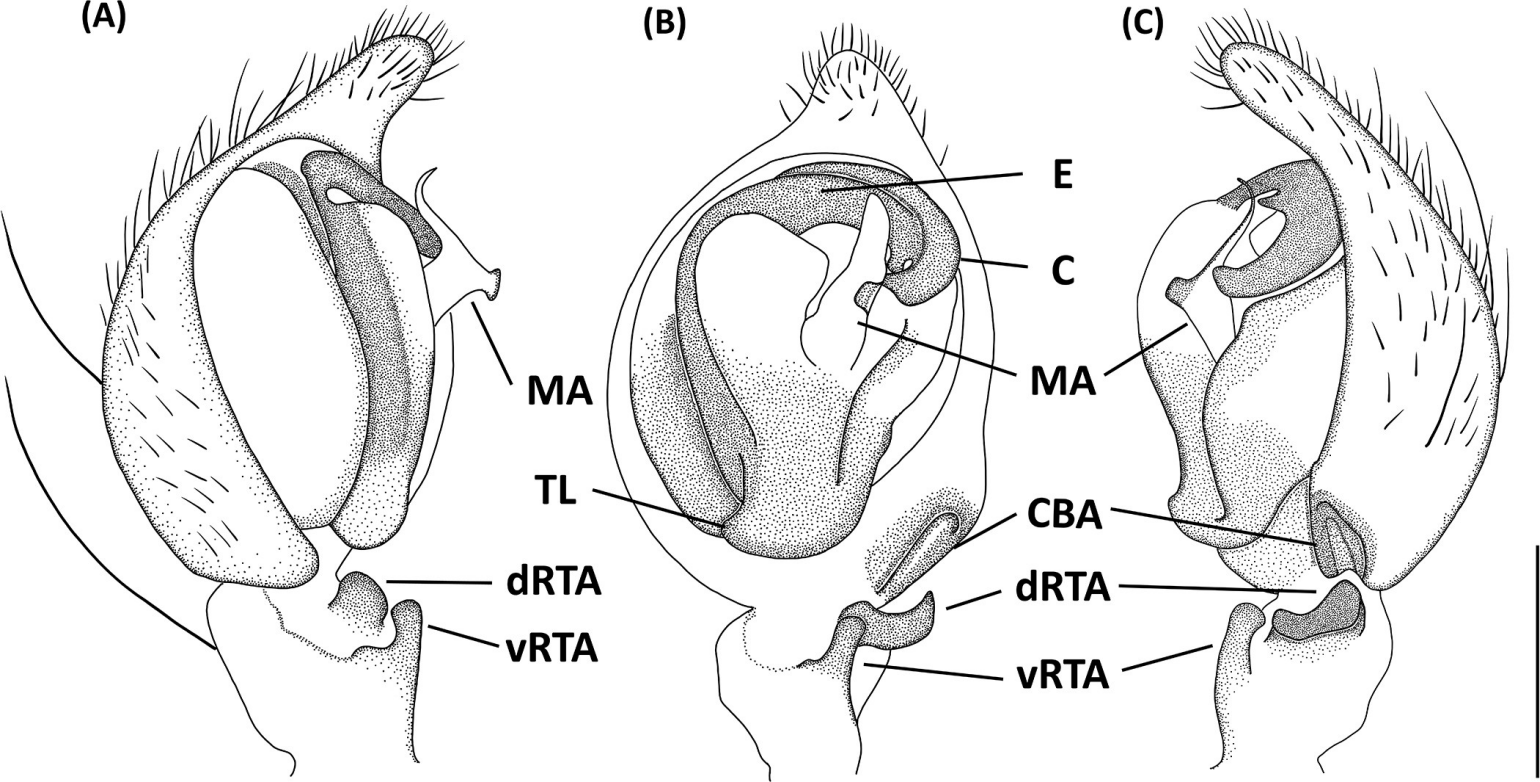
Left palp of *Hamataliwa leporauris* sp. nov., male. A, prolateral view; B, dorsal view; C, retrolateral view. Scale bar = 0.5 mm.

#### Type material. Holotype

**TAIWAN. *Kaohsiung City*:** one male (TESRI-C040071), 12 Mar. 2016, Duona Forest Road, (22.8880 N, 120.7379 E; 1050m elevation), Yu-Da Lai leg. **Paratype**. Two males (TESRI-C040064/C040073), sampling data same as holotype; two females (TESRI-Ar1762–1763), 15 May 2016, sampling location same as holotype, Yu-Da Lai leg.

Other material examined

***Taitung County*:** Lijia forest Road (22.8031 N, 121.0508 E, 360m elevation), one female (TESRI-Ar3162), 19 May 2019, Chang-Lin Zhong leg.

#### Diagnosis

This species can be distinguished from other congeneric species by the tip of MA, which is slender and elongate upward (Figs 15D and 16C), and by the vulva with a pair of prolonged, hare ear-like spermatheca (Figs 13C and 14D).

#### Description

Female (TESRI-Ar1762, Paratype). Total length 8.9; carapace length 3.9, width 2.9; abdomen length 5.0, width 2.8. Carapace yellowish, with dark eye region and a pair of brown lines on lateral margins. Radial furrows indistinct and fovea longitudinal. In dorsal view, eyes arranged in four rows with AER strongly recurved and PER strongly procurved. Eye diameters and inter-distances: AME 0.12, ALE 0.24, PME 0.18, PLE 0.18, eye sizes ALE > PME = PLE > AME; MOA-L 1.10, MOA-AW 0.38, MOA-PW 0.72; AME-I 0.16, PME-I 0.38, AML-I 0.10, PML-I 0.36; clypeus height 0.82. Chelicerae yellowish, downward with two promarginal teeth and one retromarginal tooth. Endite and labium longer than wide. Sternum yellowish, heart-like. Abdomen long oval with a grey cardiac mark, lateral region dark. In ventral view, a broad dark longitudinal band extend from epigastric furrow to spinnerets (Fig 13A). Legs yellowish, clothed with many conspicuous long spines. Three claws. Pedipalps bear apical claw. Measurements of pedipalps and legs: pedipalp 3.6 (1.1, 0.6, 0.7, 1.2), leg I 13.6 (4.1, 1.4, 3.7, 3.0, 1.4), leg II 12.6 (3.7, 1.4, 3.5, 2.8, 1.2), leg III 10.2 (3.2, 1.2, 2.4, 2.4, 1.0), leg IV 9.4 (2.8, 1.1, 2.2, 2.4, 0.9). Leg formula: I > II > III > IV.

Epigynal posterior sclerotized edge thick, central depression heart-like (Figs 13B, 13C, 14A and 14B). Spermathecae large and prolonged. A pair of spemathecae formed into hare ear-like shape.

Male (TESRI-C040071, Holotype). Body shape and color pattern similar to that of female. Total length 7.0; carapace length 3.2, width 2.4; abdomen length 3.8, width 1.9. Eye diameters and inter-distances: AME 0.10, ALE 0.22, PME 0.18, PLE 0.18, eye sizes ALE > PME = PLE > AME; MOA-L 0.82, MOA-AW 0.34, MOA-PW 0.62; AME-I 0.16, PME-I 0.32, AML-I 0.10, PML-I 0.34; clypeus height 0.64. Measurements of pedipalps and legs: pedipalp 2.9 (0.8, 0.4, 0.4, 1.3), leg I 11.9 (3.5, 1.0, 3.4, 3.0, 1.0), leg II 11.5 (3.3, 1.1, 3.1, 2.8, 1.2), leg III 9.0 (2.7, 1.0, 2.2, 2.2, 0.9), leg IV 8.9 (2.6, 0.9, 2.0, 2.5, 0.9). Leg formula: I > II > III > IV.

Papal tibia with two apophyses: ventral retrolateral tibia apophysis rod-like in ventral view, dorsal retrolateral tibial apophysis larger, protrude laterally and bend upward at distal part. Cymbium with a basal auricular outgrowth. Median apophysis with the membranous base, the tip slender and elongate upward in lateral view. Embolus relatively thick. Conductor wide, distal part sickle-like (Figs 15B–15D and 16A–16C).

#### Distribution

Endemic to Taiwan.

#### Etymology

The specific epithet ‘*leporauris*’ is a noun from the combination of the Latin *lepus*, Genitive case *leporis* (hare) and *auris* (ear), referring to the shape of spermatheca similar to hare’s ears.

### Genus *Peucetia* Keyserling, 1887

#### Diagnosis

*Peucetia* is similar to *Schaenicoscelis* and *Tapinillus* in absence of teeth in chelicerae, male palp with a paracymbium and a long median apophysis, and ALE row wider than PME row, but can be distinguished by following characters: (1) PER slightly procurved (almost strait in *Tapinillus*); (2) clypeus high, face vertical, and ocular arear relatively small (clypeus length equal or shorter than ocular area in *Tapinillus* and *Schaenicoscelis*) [3,4].

#### *Peucetia latikae* Tikader, 1970

*Peucetia latikae* Tikader, 1970: figs 49A–49C [115]; Hu *et al*. 1987: figs. 2A–2D [120]; Chen and Gao 1990: fig. 180 [121]; Gajbe 1999: figs. 90–93 [122]; Gajbe 2008: figs. 43–46 [123].

**Fig 17:**
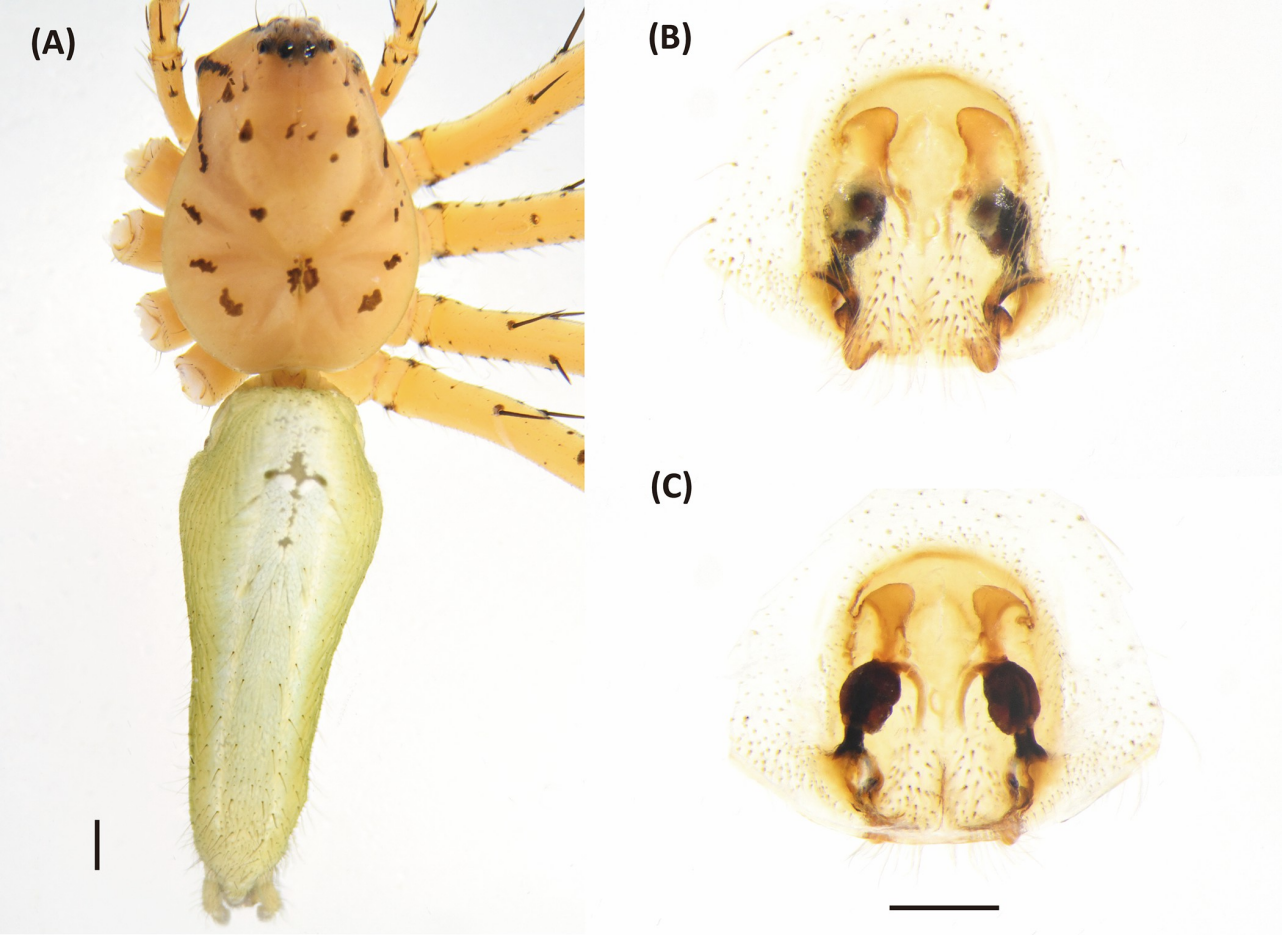
*Peucetia latikae*, female. A, Habitus, dorsal view; B–C, Epigyne (B, ventral view; C, dorsal view). Scale bar: A = 1 mm; B–C = 0.5 mm.

**Fig 18:**
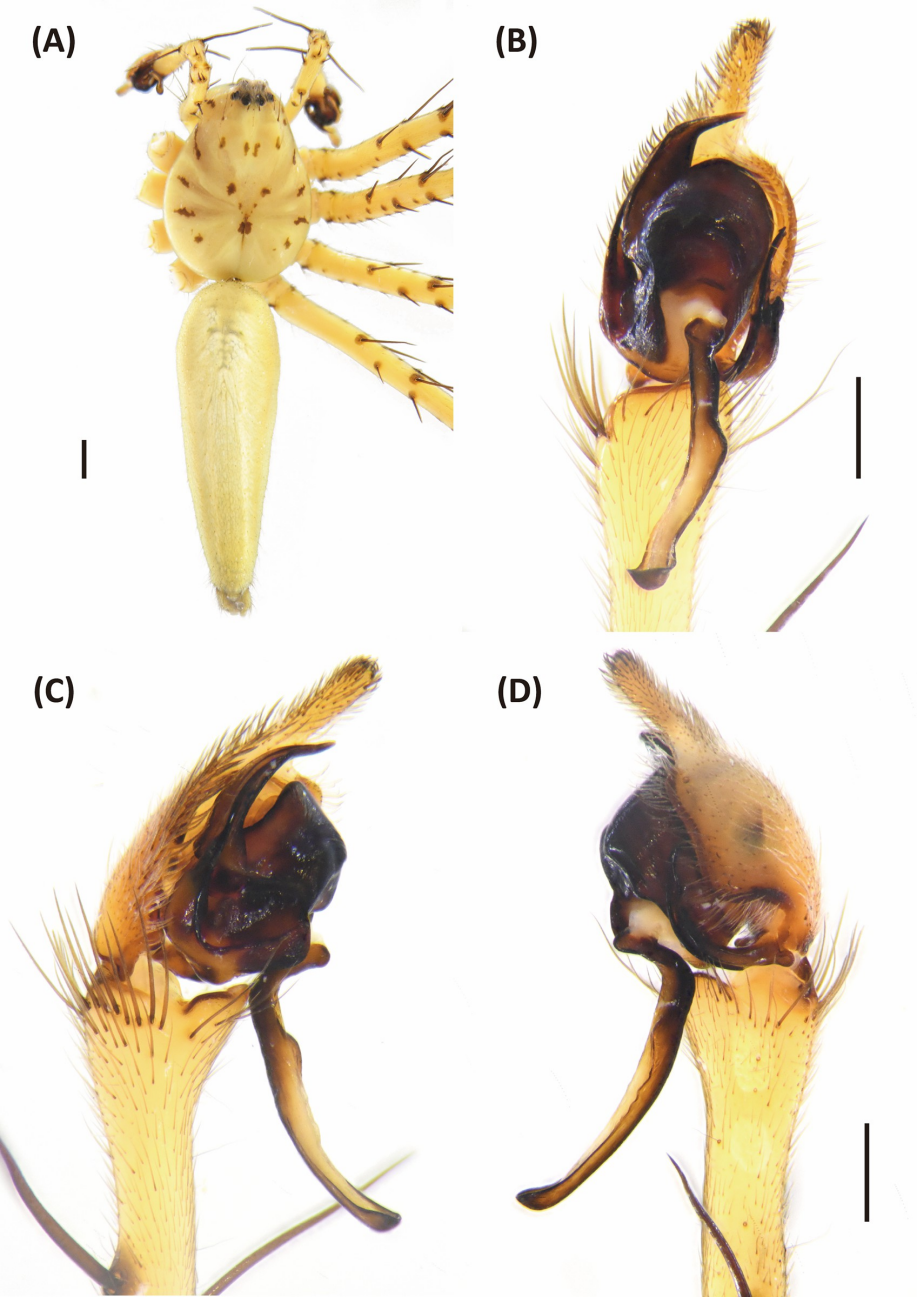
*Peucetia latikae*, male. A, Habitus, dorsal view; B–D, Palp (B, ventral view; C, prolateral view; D, retrolateral view). Scale bar: A = 1 mm; B–D = 0.5 mm.

Figs 17 and 18, S2G Fig.

#### Material examined. TAIWAN

***Xinchu County*:** Beipu (24.7080 N, 121.0562 E; 130m elevation), one male (TESRI-Ar5701), 29 Aug. 2020, Kuang-Ping Yu leg. ***Taichung City*:** Dakeng (24.1739 N, 120.7819 E; 430m elevation), two males (TESRI-Ar5516–5517), 11 Aug. 2011, Ren-Chung Chang leg., one female (TESRI-Ar5702), 12 Aug. 2020, Pin-Huang Hsu leg. ***Nantou County*:** Zhongliao (23.8997 N, 120.7621 E; 170m elevation), one male (TESRI-CX019), 11 May 2018, Ying-Yuan Lo leg., one male (TESRI-CX259), 29 Aug. 2019, Ying-Yuan Lo leg.; Endemic Species Research Institute (23.8293 N, 120.8014 E; 250m elevation), one male and two females (TESRI-Ar1867/1868/1873), 06 Aug. 2016, Yu-Da Lai leg.; Zhongxing New Village (23.9444 N, 120.7042 E, 125), one female (TESRI-Ar1880), 06 Sep. 2016, Chong-Sheng Huang leg.; Fengzikeng trail (23.9612 N, 120.7088 E; 183m elevation), one male (TESRI-Ar1881), 07 Sep. 2016, Hui-Ling Chen leg.; Shuang Wen Junior High School (23.9330 N, 120.7610 E; 208m elevation), one female (TESRI-Ar1882), 07 Sep. 2016, Chong-Sheng Huang leg.; Taomikeng (23.8582 N, 120.7678 E; 278m elevation), five females (TESRI-Ar1884–1888), 06 Sep. 2016, Chong-Sheng Huang leg. ***Changhua County*:** Qingshuiyan (23.8843 N, 120.6131 E; 90m elevation), one female (TESRI-Ar5515), 28 Aug. 2019, Kuang-Ping Yu leg. ***Tainan City*:** Fengde Power Plant (23.0840 N, 120.3558 E; 50m elevation), one male (TESRI-Ar4230), 28 Feb. 2019, Kuang-Ping Yu leg. ***Pingtung County*:** Dahanshan (22.4083 N, 120.6482 E; 525m elevation), one male (TESRI-Ar5752), 13 Sep. 2020, Chang-Lin Chuang leg.

#### Diagnosis

This species resembles *P*. *viridis* (Blackwall, 1858) in the male palp organ with long, curved and dorsally hollow median apophysis, and female epigyne with an anterior pit [124], but can be distinguished from latter by following characters: (1) the paracymbium long, arched, and acuminated distally (Fig 17B and 17D); (2) copulatory opening notch-like (Fig 16B); (3) spermatheca rounded, and situated anteriorly to the copulatory ducts (Fig 17C).

#### Description

Female (TESRI-Ar1868). Total length 16.8; carapace length 7.1, width 5.2; abdomen length 9.2, width 4.0. Carapace greenish, with scattered black patches or stripes. Radial furrows indistinct and fovea longitudinal. In lateral view, carapace gradually descend from fovea region to posterior edge. Ocular region with dense pale white hairs. Eyes arranged in four rows with AER strongly recurved and PER strongly procurved. Eye diameters and inter-distances: AME 0.14, ALE 0.32, PME 0.24, PLE 0.24, eye sizes ALE > PME = PLE > AME; MOA-L 1.22, MOA-AW 0.48, MOA-PW 0.80; AME-I 0.20, PME-I 0.32, AML-I 0.14, PML-I 0.24; clypeus height 1.80. Chelicerae greenish, downward, with two pair of black longitudinal stripes which one pair from anterior eyes extended to terminal of chelicerae and one pair from lateral head extended to medium of chelicerae in front side. Chelicerae with no tooth. Endite and labium yellowish, longer than wide. Sternum yellowish, with few erect setae, and one longitudinal stripe from center to posterior edge. Abdomen green, long oval, with green-yellowish cardiac mark, and a pair of yellow longitudinal stripes from anterior margin extended to spinnerets (Fig 17A). Legs light greenish, more or less transparent in alive individuals, clothed with many conspicuous long, erect spines, and scattered several black spots. Three claws. Pedipalps bear apical claw. Measurements of pedipalps and legs: pedipalp 8.9 (2.7, 1.2, 1.9, 3.1), leg I 38.2 (10.0, 2.7, 10.3, 10.4, 4.8), leg II 32.2 (8.9, 2.5, 8.5, 8.6, 3.7), leg III 25.3 (7.4, 2.4, 6.4, 6.6, 2.5), leg IV 28.9 (8.4, 2.4, 7.4, 7.9, 2.8). Leg formula: I > II > IV > III.

Epigynal posterior edge with a pair of downward prominent. Copulatory open large, notch-like and situated at lateral side. Anterior edge with a pair of horn-like prominent (Fig 17B). Spermathecae nearly spherical, and situated anterior side of copulatory ducts. Fertilized ducts slender and curve. Copulatory ducts thick (Fig 17C).

Male (TESRI-Ar1867). Body shape and color pattern similar to that of female. Femur I with large pink patch. Total length 137; carapace length 53, width 41; abdomen length 8.4, width 29. Eye diameters and inter-distances: AME 0.12, ALE 0.28, PME 0.22, PLE 0.22, eye sizes ALE > PME = PLE > AME; MOA-L 1.06, MOA-AW 0.40, MOA-PW 0.64; AME-I 0.16, PME-I 0.20, AML-I 0.10, PML-I 0.24; clypeus height 1.20. Measurements of pedipalps and legs: pedipalp 9.5 (4.0, 1.5, 2.1, 1.9), leg I 42.6 (10.5, 2.1, 10.5, 12.5, 7.0), leg II 33.8 (9.2, 2.1, 8.7, 9.5, 4.3), leg III 25.0 (7.5, 1.9, 6.2, 7.0, 2.4), leg IV 28.7 (8.3, 1.9, 7.4, 8.3, 2.8). Leg formula: I > II > IV > III.

Palpal tibia longer than bulbus, and armed with three long bristle. Paracymbium long, arced, and acuminates distally. Median apophysis downward elongated with slightly dorsal concave and spoon-like tip. Conductor short, heavy basally. Embolus thin, and tip covered by conductor (Fig 18B–18D).

#### Distribution

India, China and Taiwan (newly recorded)

#### Remarks

*Peucetia formosensis* Kishida, 1930 was another congeneric species in Taiwan. Kishida [9] reported the female and pointed out that the species distributed in six localities. Then, Kayashima [125] reported the male specimen collected from Taitung County. However, while we collected and examined *Peucetia* specimens throughout Taiwan, both morphological and DNA barcoding evidence supported that they are *P*. *latikae*. Because the type specimens of *P*. *formosensis* remain unknown, and there is no further record of the species after Kishida [9] and Kayashima [125], we suggest that *P*. *formosensis* should be *nomen dubium*.

### Genus *Tapponia* Simon, 1885

#### Diagnosis

**Fig 19:**
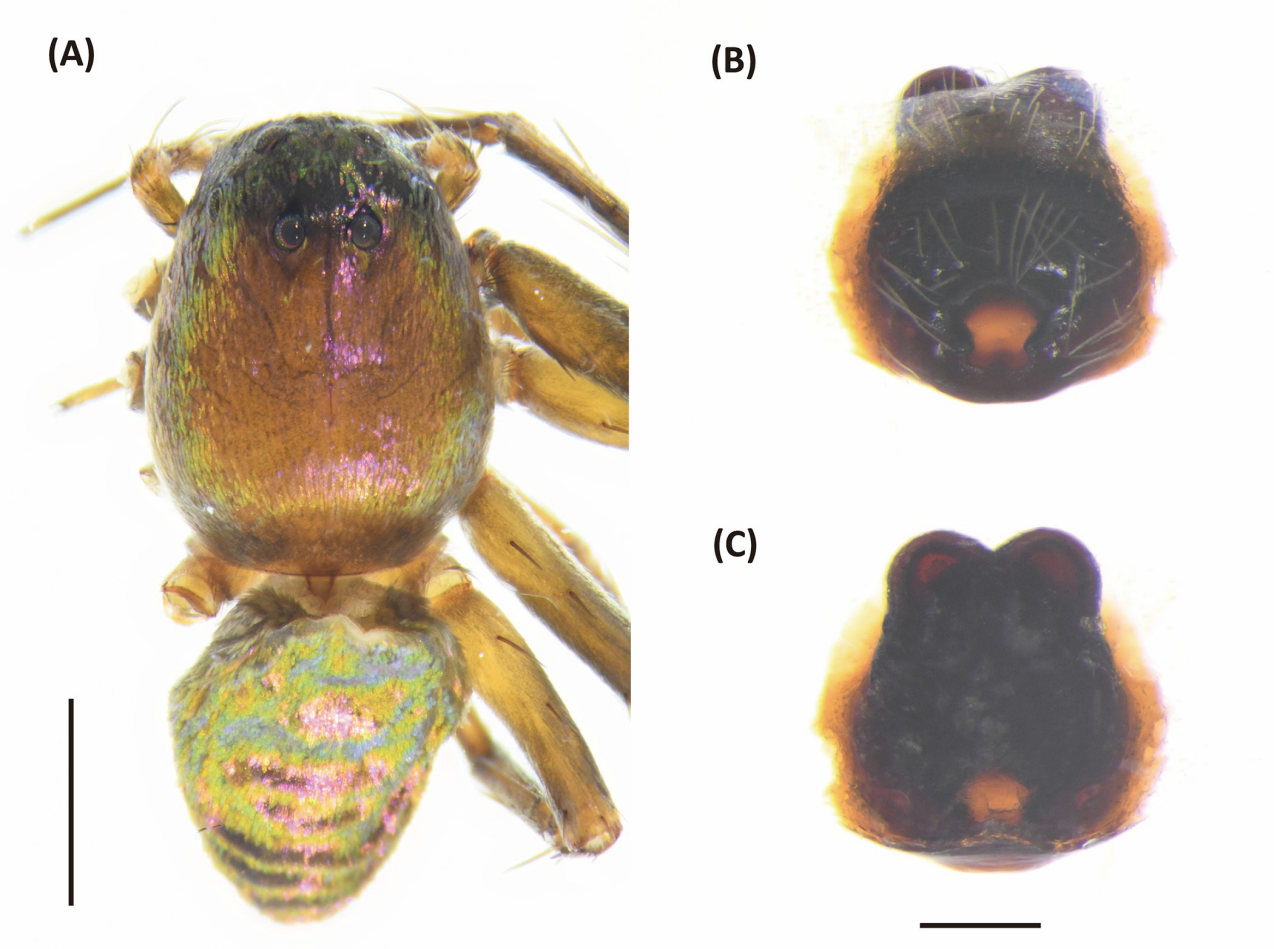
*Tapponia auriola* sp. nov., female. A, Habitus, dorsal view; B, Epigyne; C, Vulva. Scale bar: A = 1 mm; B–C = 0.2 mm.

*Tapponia* is similar to *Hamadruas* and *Hamataliwa* in male palp with a basal apophysis on cymbium and with a tegular lobe, but can be distinguished from latter by following characters: (1) width of ocular area almost equal to those of head region (Figs 19A, 21A and 23A; width of ocular area shorter those of head region in *Hamadruas* and *Hamataliwa*); (2) clypeus length shorter than ocular area, (equal or shorter in *Hamadruas* and *Hamataliwa*); (3) carapace and abdomen with iridescent scales (except in *T*. *parva* and *T*. *rarobulbus*; Fig 19A); (4) male pedipalp with a patellar apophysis (except in *T*. *parva* and *T*. *rarobulbus*; Fig 21B and 21D) [7]; (5) smaller body size (3–4 mm in *Tapponia*, whereas most species larger than 3 mm in *Hamadruas* and *Hamataliwa*).

#### *Tapponia auriola* sp. Nov

**Fig 20:**
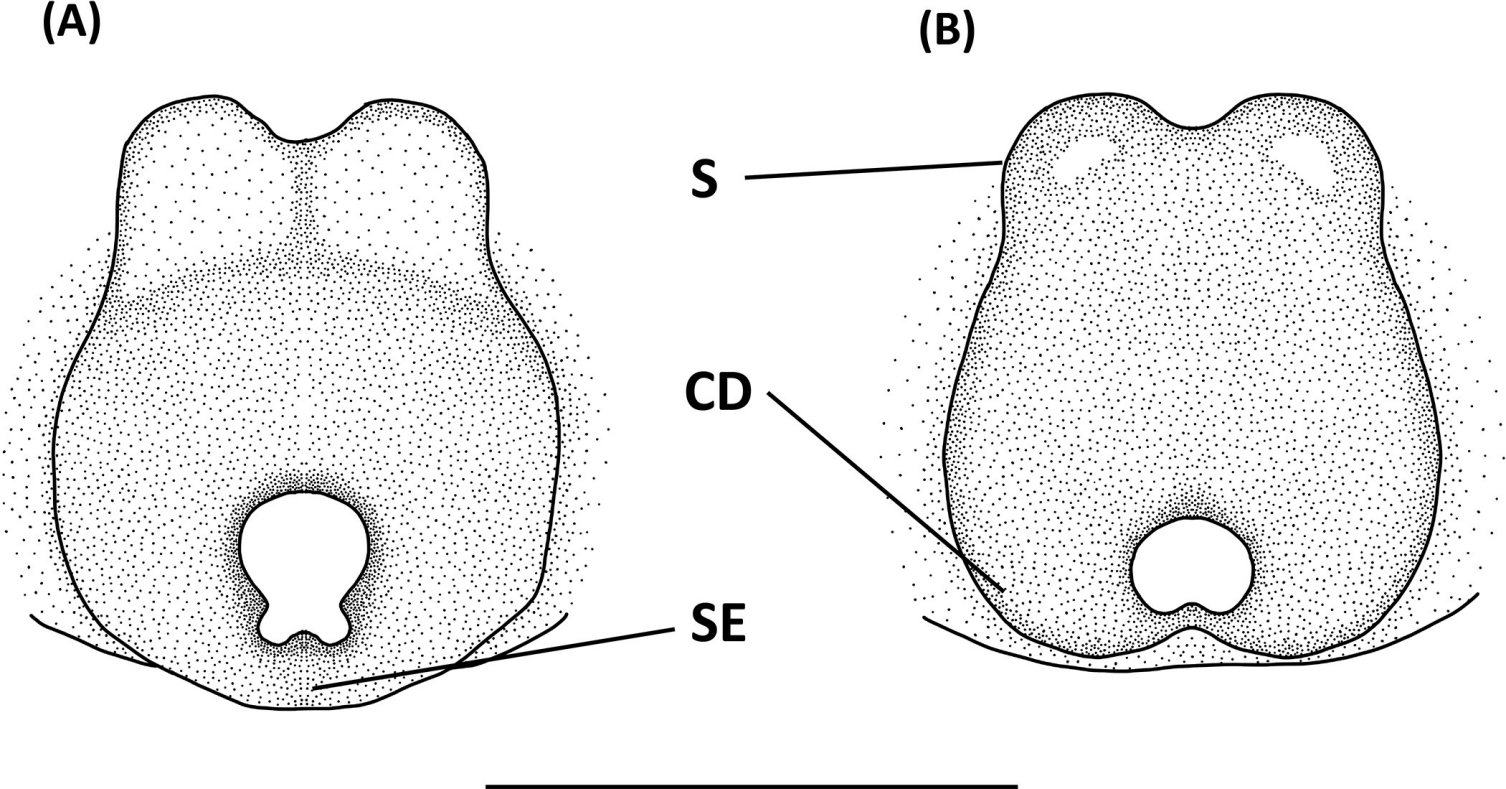
Epigyne and vulva of *Tapponia auriola* sp. nov., female. A, epigyne; B, vulva. Scale bar = 0.5 mm.

**Fig 21:**
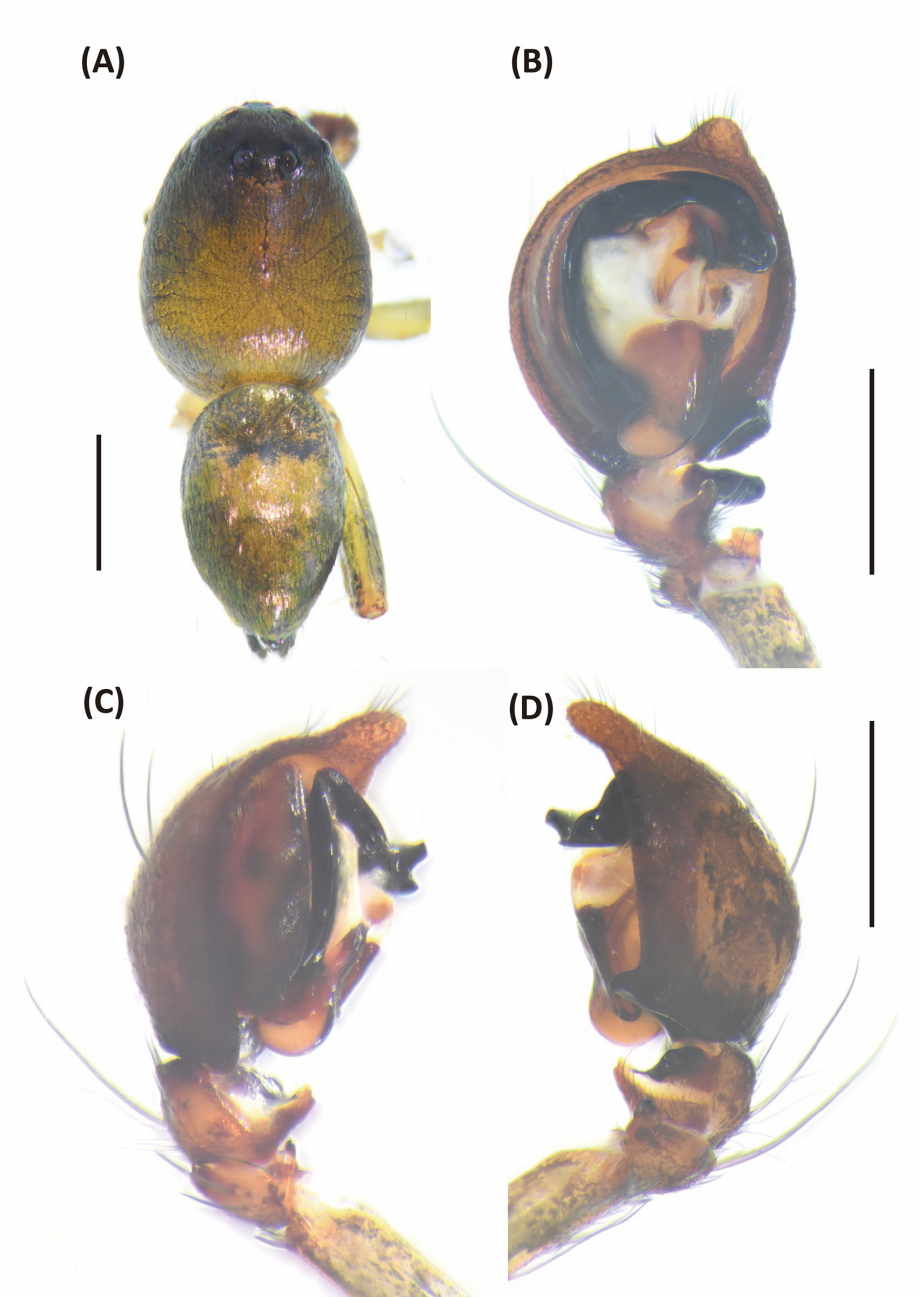
*Tapponia auriola* sp. nov., male. A, Habitus, dorsal view; B–C, Palp (B, ventral view; C, prolateral view; D, retrolateral view). Scale bar: A = 1 mm; B–C = 0.5 mm.

**Fig 22:**
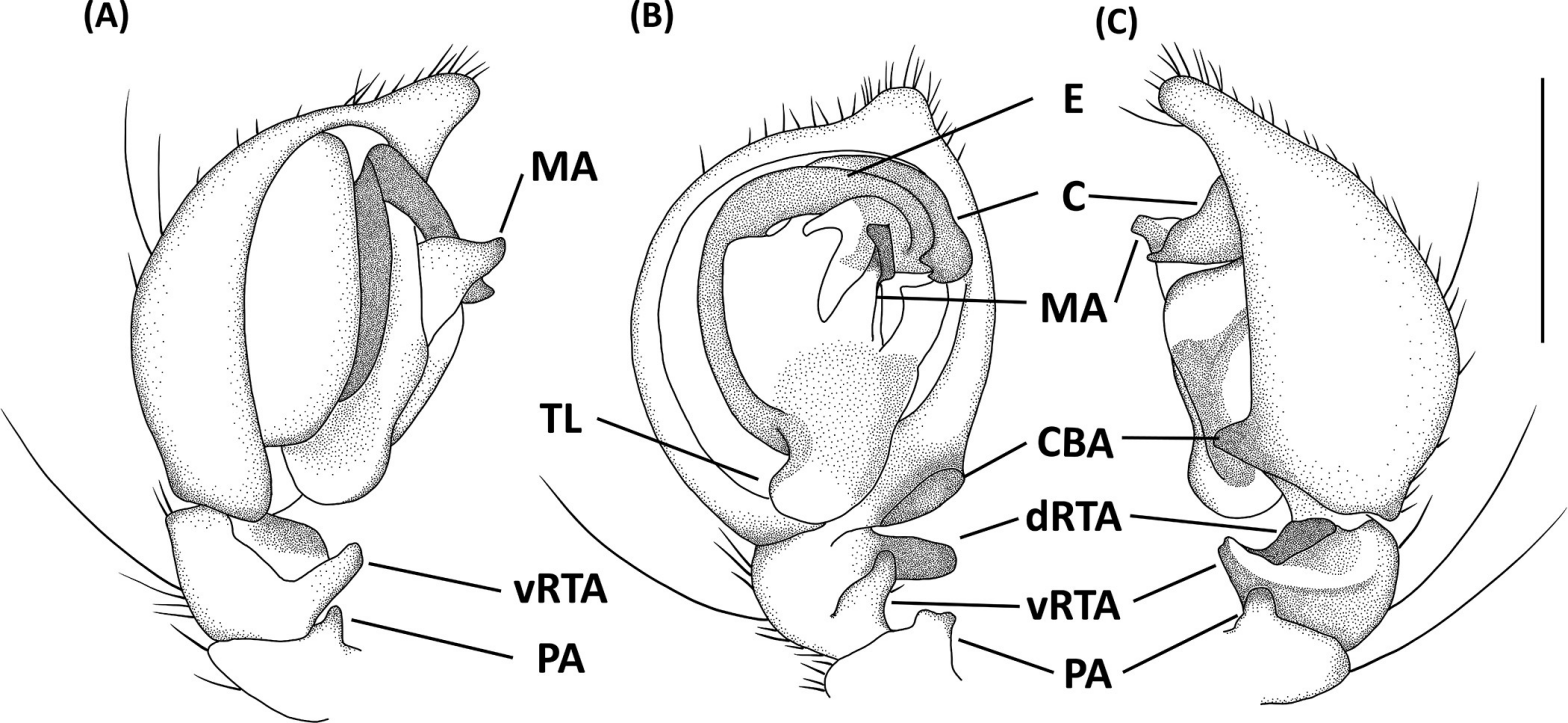
Left palp of *Tapponia auriola* sp. nov., male. A, prolateral view; B, ventral view; C, retrolateral view. Scale bar = 0.5 mm.

Figs 19–22, S2H Fig.

#### Type material. Holotype

**TAIWAN. *Taichung City*:** one male (TESRI- Ar5755), 22 May 2021, Wushikeng (24.2958 N, 120.9282 E; 560m elevation), Zheng-Di Li leg. **Paratype. *Nantou County*:** one female (TESRI-Ar3146), 17 Jul. 2018, Lianhuachi, (23.9183 N, 120.8888 E; 655m elevation), Wen-Chun Huang leg. ***Taichung City*:** one male (TESRI-Ar5722), 13 Jun. 2020, Baxianshan (24.1898 N, 121.0156 E; 951m elevation), Anansi Brigade leg.

#### Diagnosis

*Tapponia micans* Simon, 1885 is the only species of the monotypic genus. Both *T*. *auriola* **sp. nov.** and *T*. *micans* have iridescent scales, male palp organ with a U-shaped tegular lobe and a transverse retrolateral apophysis, but the former can be distinguished from the latter by the following characters: (1) dRTA situated distally (Figs 21B and 22B; proximally in *T*. *micans*); (2) the tip of MA flat and wide (acuminate in *T*. *micans*); (3) female internal genitalia present a central strongly sclerotized lump (Figs 19C and 20B; central void in *T*. *micans*).

#### Description

Female (TESRI-Ar3146, paratype). Total length 4.2; carapace length 2.2, width 1.7; abdomen length 2.0, width 1.4. Carapace oval-shaped, brown, covered with iridescent scales (metallic green while alive). Radial furrows indistinct and fovea longitudinal. Eyes arranged in four rows with AER strongly recurved and PER strongly procurved. Eye diameters and inter-distances: AME 0.08, ALE 0.20, PME 0.14, PLE 0.16, eye sizes ALE > PLE > PME > AME; MOA-AW 0.26, MOA-PW 0.50, MOA-L 0.84; AME-I 0.10, PME-I 0.20, AML-I 0.06, PML-I 0.30; clypeus height 0.30. Chelicerae yellowish brown, downward, with two promargianl teeth and one retromarginal tooth. Endite yellowish, longer than wide. Sternum yellowish, with few setae. Legs yellowish, clothed with several conspicuous spines. Three claws. Pedipalps bear apical claw. Measurements of pedipalps and legs: pedipalp 2.0 (0.5, 0.3, 0.4, 0.8), leg I 7.2 (2.1, 0.6, 2.1, 1.6, 0.8), leg II 6.2 (1.9, 0.6, 1.8, 1.3, 0.6), leg III 4.6 (1.3, 0.5, 1.0, 1.1, 0.5), leg IV 4.5 (1.4, 0.5, 1.0, 1.1, 0.5). Leg formula: I > II > III = IV. Abdomen oval-shaped, yellowish brown, covered dense iridescent scales. Cardiac mark indistinct (Fig 19A).

Epigyne strongly sclerotized, posterior part with a small open depression (Figs 19B and 20A). Spermathecae globular, visible though external cuticle. Internal genitalia present a central sclerotized lump. Copulatory ducts thick.

Male (TESRI-Ar5755, Holotype). Body shape and color pattern similar to that of female (Fig 21A). Total length 4.0; carapace length 2.2, width 1.7; abdomen length 1.8, width 1.2. Eye diameters and inter-distances: AME 0.06, ALE 0.16, PME 0.14, PLE 0.14, eye sizes ALE > PME = PLE > AME; MOA-AW 0.24, MOA-PW 0.48, MOA-L 0.78; AME-I 0.12, PME-I 0.20, AML-I 0.06, PML-I 0.30; clypeus height 0.38. Measurements of pedipalps and legs: pedipalp 2.0 (0.6, 0.3, 0.3, 1.0), leg I 7.2 (2.1, 0.6, 2.1, 1.6, 0.8), leg II lost, leg III 4.7 (1.5, 0.5, 1.1, 1.1, 0.5), leg IV 4.7 (1.4, 0.4, 1.0, 1.4, 0.5). Leg formula: I > III = IV.

Patella of pedipalp with a digit-shaped retrolateral apophysis. Ventral RTA digit-shaped in ventral view, join a posterior cavity-shaped depression. Dorsal RTA chunk-shaped, and protrude retrolaterally. Basal outgrowth of cymbium with a furrow. Tegulum lobe conspicuous. Median apophysis with the membranous base, and the black, blunt tip. Embolus twist, the distal part behind the conductor. Conductor thick, distal part sickle-like (Figs 21B֪–21D and 22A–22C).

#### Distribution

Endemic to Taiwan.

#### Remarks

Deeleman-Reinhold [7] reviewed lynx spiders from forest canopy of Borneo and transferred five species of *Tapponia* to *Hamataliwa* and nine species to *Hamadruas*. Currently, the genus *Tapponia* contains only one species, *T*. *micans*, which is distributed in Malaysia, Sumatra and Borneo. Although we couldn’t borrow the holotype of *T*. *micans*, the descriptions of the species by Deeleman-Reinhold [7,126] are explicit for confirming genitalia structure of *T*. *auriola* is distinct from those of *T*. *micans*.

#### Etymology

This specific epithet *auriolus*, f. *auriola*, n. *auriolum* is a Latin adjective, which means brilliant and golden, referring to the shiny body color of this species.

#### *Tapponia parva* sp. Nov

Figs 23 and 24

**Fig 23:**
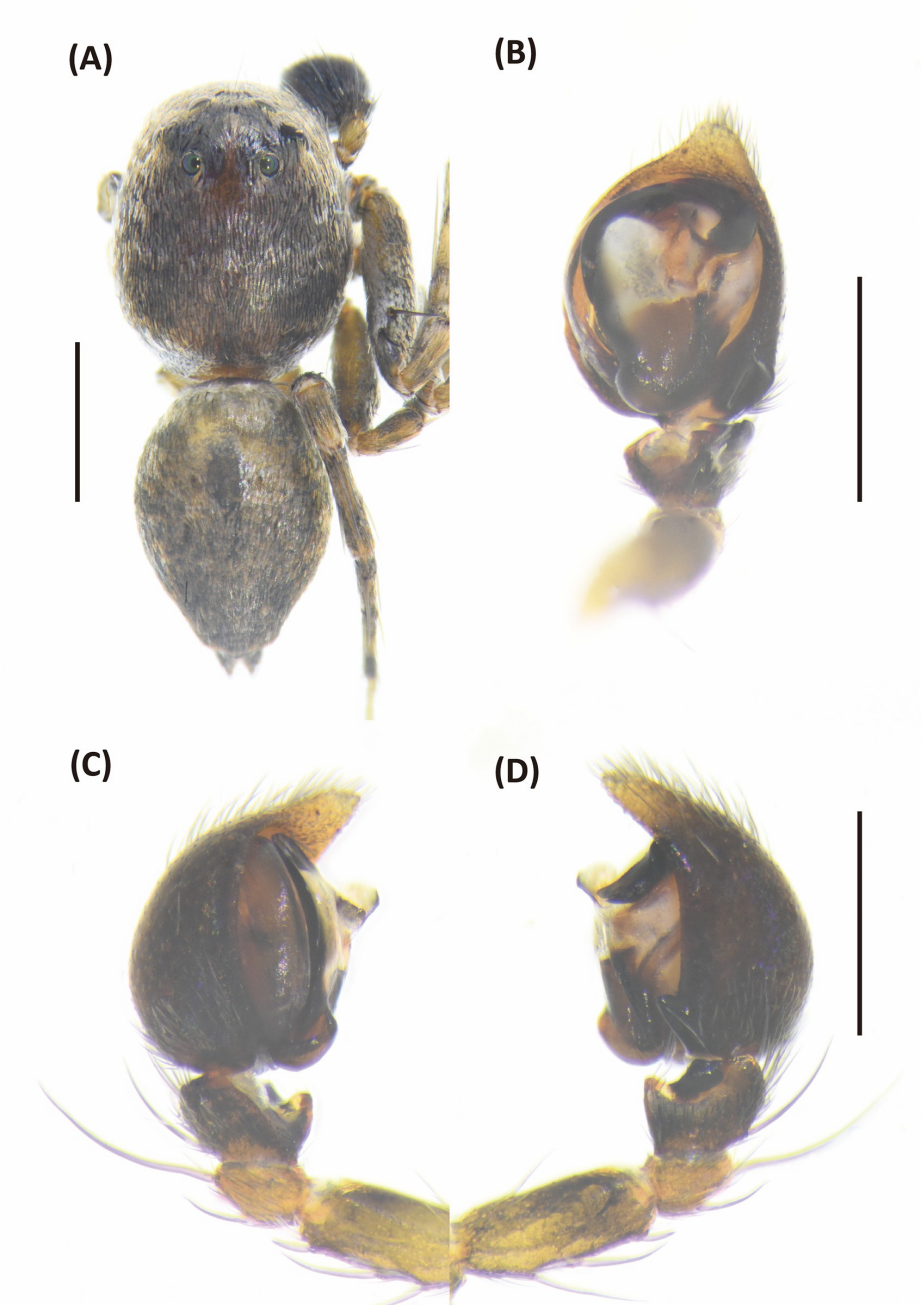
*Tapponia parva* sp. nov., male. A, Habitus, dorsal view; B–D, Palp (B, ventral view; C, prolateral view; D, retrolateral view). Scale bar: A = 1 mm; B–D = 0.5 mm.

**Fig 24:**
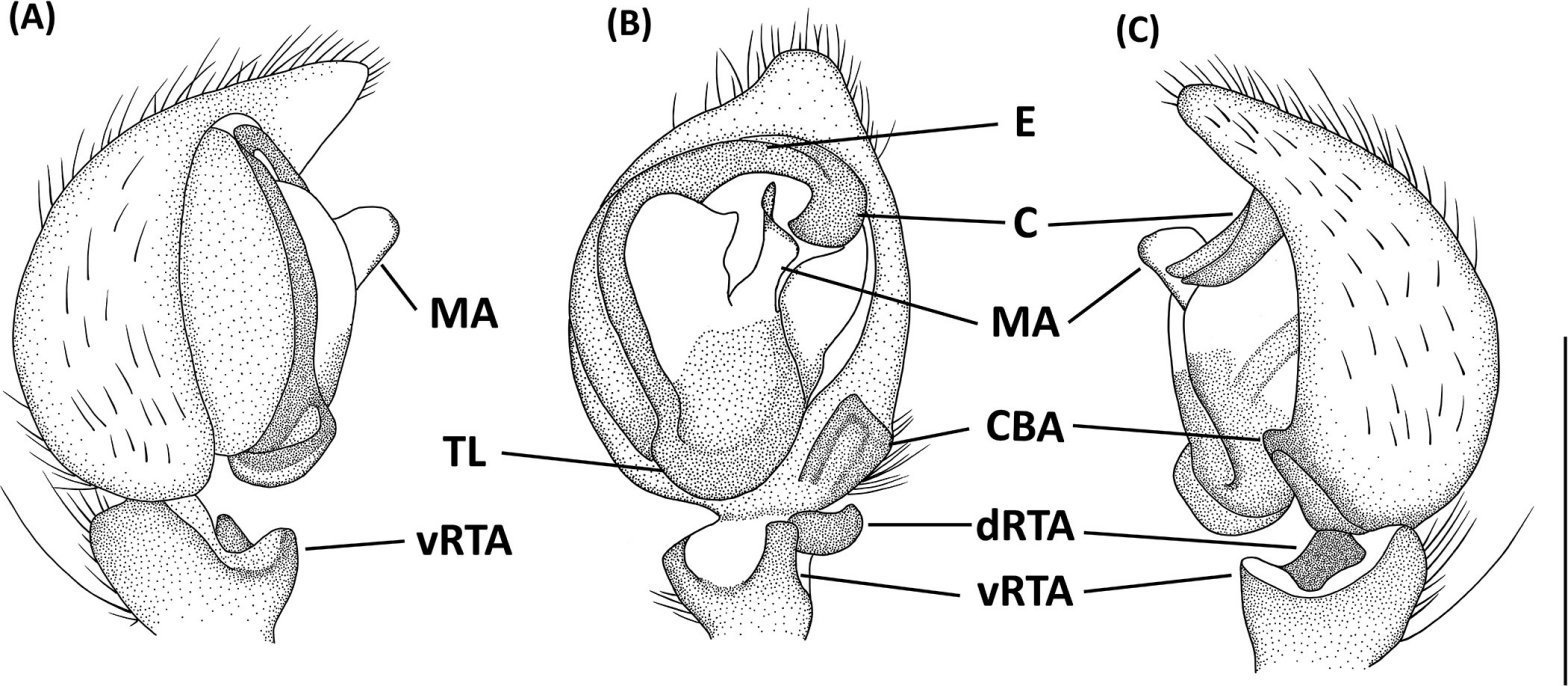
Left palp of *Tapponia parva* sp. nov., male. A, prolateral view; B, ventral view; C, retrolateral view. Scale bar = 0.5 mm.

#### Type material. Holotype

**TAIWAN. *Yilan County*:** one male (TESRI-Ar5754), 13 Apr. 2021, Guanyin tunnel (24.4360 N, 121.7742 E;70m elevation), Chi-Wei leg. **Paratype**

***Nantou County*:** one male (TESRI-Ar2983), 14 Oct. 2018, Jiufenershan (23.9495 N, 120.8548 E; 535m elevation), Thiao-Tien Chang leg.

#### Diagnosis

*Tapponia parva* **sp. nov.** is similar to *T*. *auriola* **sp. nov.** in body shape and structure of male palp organ, but can be distinguished from latter by the following characters: (1) abdomen is not covered by iridescent scales; (2) dRTA is smaller, and slight upturned distally (Fig 23B–23D; larger and not present upturned in *T*. *auriola*); (3) tegulum lobe is wider, and less expand downward (Fig 23B); (4) the tip of MA is membranous (Fig 23D; black and blunt tip in *T*. *auriola*).

#### Description

Male (TESRI-Ar5754, Holotype). Total length 3.4; carapace length 1.8, width 1.5; abdomen length 1.6, width 1.2. Carapace dark brown, near round shape, covered with dense long scale. Fovea longitudinal, cervical and radial furrows indistinct. Head region semi-circle shape in font view. In dorsal view, eyes arranged in four rows with AER strongly recurved and PER strongly procurved. Eye diameters and inter-distances: AME 0.06, ALE 0.16, PME 0.12, PLE 0.12, eye sizes ALE > PME = PLE > AME; MOA-L 0.70, MOA-AW 0.24, MOA-PW 0.60; AME-I 0.12, PME-I 0.36, AML-I 0.06, PML-I 0.22; clypeus height 0.48. Chelicerae brown, downward with 1 promarginal teeth and without retromarginal tooth. Endite yellowish, longer than wide. Sternum yellowish, heart-like. Abdomen oval, with a dark cardiac mark, and scattered irregular spots. Legs brown, clothed with many conspicuous long spines. Three claws. Measurements of pedipalps and legs: pedipalp 1.5 (0.4, 0.2, 0.2, 0.7), leg I 5.4 (1.5, 0.5, 1.3, 1.4, 0.7), leg II 4.4 (1.3, 0.4, 1.1, 1.0, 0.6), leg III 3.1 (1.0, 0.4, 0.6, 0.7, 0.4), leg IV 3.8 (1.2, 0.4, 0.7, 1.0, 0.5). Leg formula: I > II > IV > III.

Palpal tibia sunken retro-laterally, with a rod-like ventral RTA and a larger, protrude dorsal RTA. Basal cymbium outgrowth triangular in lateral view. Median apophysis upward, truncated and wide at tip, with the membranous base. Conductor wide, distal part sickle-like (Figs 23B–23D and 24A–24C).

#### Distribution

Endemic to Taiwan.

#### Remarks

*Tapponia parva* **sp. nov.** was obtained from two separate localities in eastern (Yilan County) and central (Nantou County) Taiwan. Further investigation is needed to fully understand its geographic distribution. The female of the species is still unknown.

#### Etymology

The specific epithet *parvus*, f. *parva*, n. *parva* is a Latin adjective, which means tiny, referring to fairly small body size of this species.

#### *Tapponia rarobulbus* sp. Nov

Figs 25 and 26.

**Fig 25:**
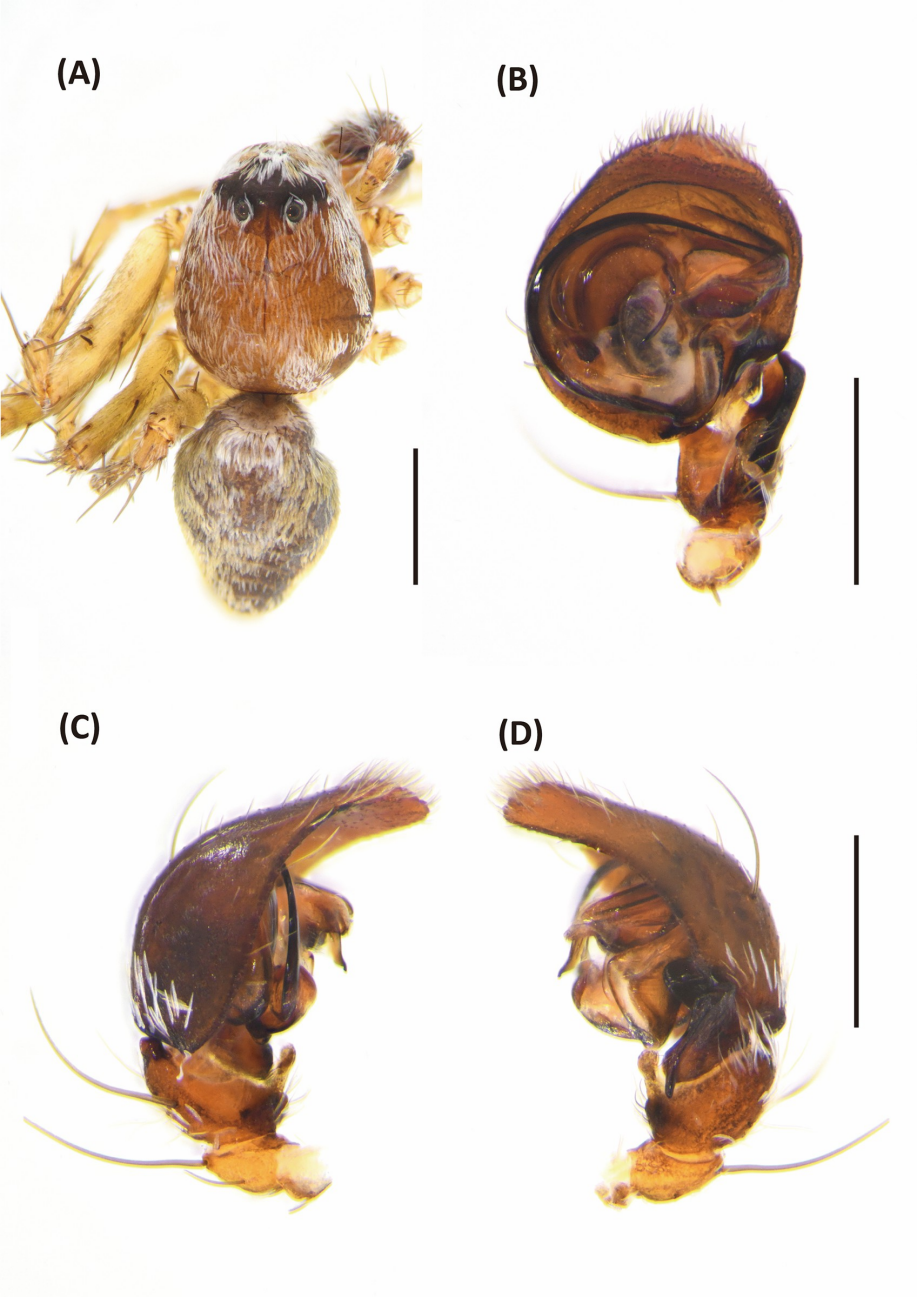
*Tapponia rarobulbus* sp. nov., male. A, Habitus, dorsal view; B–D, Palp (B, ventral view; C, prolateral view; D, retrolateral view). Scale bar: A = 1 mm; B–D = 0.5 mm.

**Fig 26:**
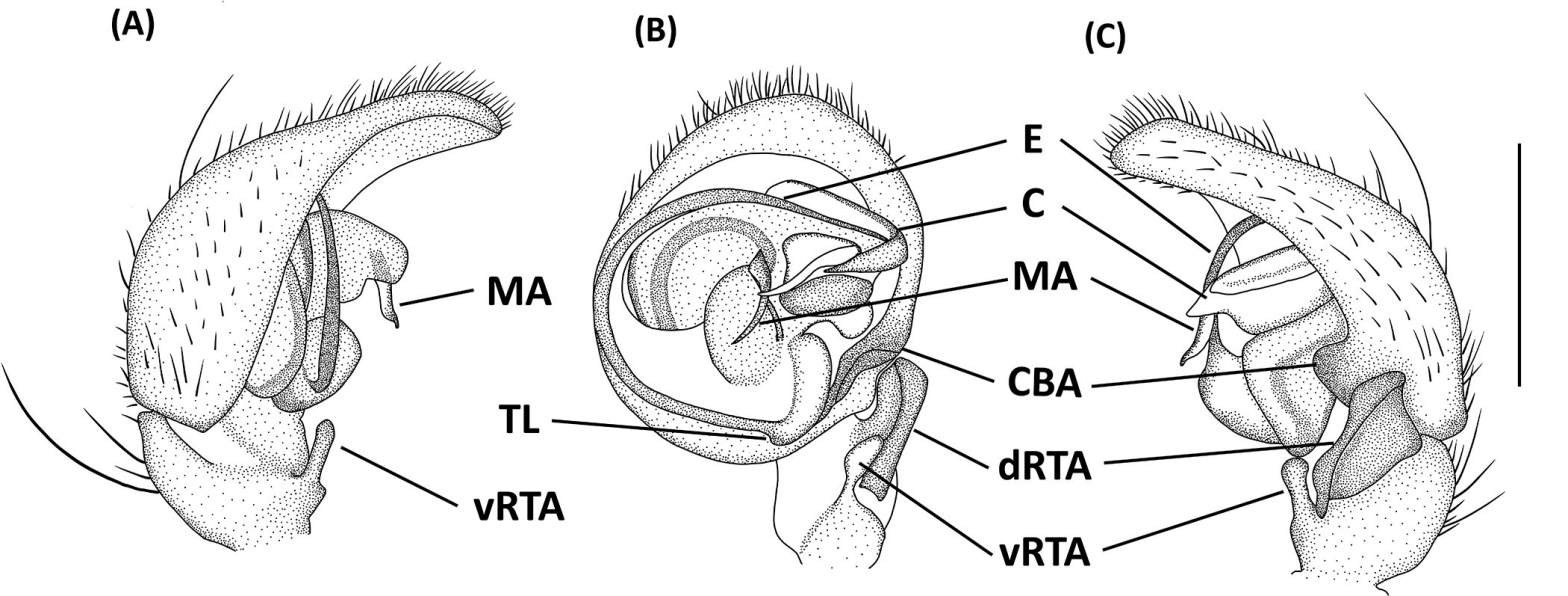
Left palp of *Tapponia rarobulbus* sp. nov., male. A, prolateral view; B, ventral view; C retrolateral view. Scale bar = 0.5 mm.

#### Type material. Holotype

**TAIWAN. *Taitung County*:** one male (TESRI- Ar3147), 10 Aug. 2018, Xiangyang (23.2477 N, 120.9866 E; 2295m elevation), Wen-Chun Huang leg.

#### Diagnosis

The palp organ structure is very different from other congeneric species, and can be recognized by following characters: (1) dRTA quite large and expanded upward (Figs 25B and 26B); (2) tegulum lobe small; (3) embolus very long and curved, wrapped around genital bulb, except on the retrolateral side (Figs 25B and 26B).

#### Description

Male (TESRI-Ar3147, Holotype). Total length 3.7; carapace length 1.8, width 1.4; abdomen length 1.9, width 1.2. Carapace oval-shaped, yellowish brown, covered with white hairs. Radial furrows indistinct and fovea longitudinal. Eye region black with dense hairs, eyes arranged in four rows with AER strongly recurved and PER strongly procurved. Eye diameters and inter-distances: AME 0.06, ALE 0.16, PME 0.12, PLE 0.12, eye sizes ALE > PLE = PME > AME; MOA-AW 0.22, MOA-PW 0.50, MOA-L 0.68; AME-I 0.10, PME-I 0.26, AML-I 0.06, PML-I 0.24; clypeus height 0.32. Chelicerae brown, downward, with one promargianl teeth and one retromarginal tooth. Endite yellowish, longer than wide. Sternum brown, heart-like shape. Legs yellowish, clothed with several long spines. Three claws. Measurements of pedipalps and legs: pedipalp 1.9 (0.5, 0.2, 0.3, 0.9), leg I 6.3 (1.8, 0.6, 1.6, 1.5, 0.8), leg II 5.4 (1.5, 0.5, 1.5, 1.4, 0.5), leg III 4.8 (1.4, 0.5, 1.1, 1.3, 0.5), leg IV 4.3 (1.1, 0.4, 1.0, 1.3, 0.5). Leg formula: I > II > III > IV. Abdomen oval-shaped, yellowish brown, covered dense white hairs. Cardiac mark indistinct.

Palpal dorsal RTA is quite large, with prolateral concave, expand upward in ventral view, and margin thicken with a downward protrude in lateral view. Ventral RTA rod-shaped, translucent. Tibia with a cavity depression. Tegulum lob small. Median apophysis stickle-like, extend downward. Embolus very long and curved, wrap around genital bulb except retrolateral side, the tip was hidden behind conductor. Conductor relatively wide, with a membranous base, the tip bend and point toward proximal (Figs 25B–25D and 26A–26C).

#### Distribution

Endemic to Taiwan.

#### Remarks

*Tapponia rarobulbus* was collected from the mid-altitudinal mountain region around 2300 meters, which represents the highest altitudinal distribution of lynx spiders in Taiwan. The female of the species is still unknown.

#### Etymology

The specific epithet ‘*rarobulbus*’ is a noun from the combination of the Latin adjective *rarus*, f. *rara*, n. rarum (unusual) and noun *bulbus* (bulb), referring to the structure of palp bulb is special in this genus.

## Supporting information

S1 Fig Bayesian inference phylogenetic tree of Oxyopidae based on *COI* dataset. (PDF)

S2 Fig Habitus of oxyopids from Taiwan. (PDF)

S1 Table Collection information of samples, and accession from GenBank used in the phylogenetic analyses. (DOCX)
