# Correction: Integrative species delimitation and five new species of lynx spiders (Araneae, Oxyopidae) in Taiwan

**DOI:** 10.1371/journal.pone.0316369

**Published:** 2024-12-19

**Authors:** Ying-Yuan Lo, Ren-Chung Cheng, Chung-Ping Lin

The Nomenclatural Acts subsection is missing from the Materials and methods section. Please see the Nomenclatural Acts subsection here.

## Nomenclatural acts

The electronic edition of this article conforms to the requirements of the amended International Code of Zoological Nomenclature, and hence the new names contained herein are available under that Code from the electronic edition of this article. This published work and the nomenclatural acts it contains have been registered in ZooBank, the online registration system for the ICZN. The ZooBank LSIDs (Life Science Identifiers) can be resolved and the associated information viewed through any standard web browser by appending the LSID to the prefix “http://zoobank.org/”. The LSID for this publication is: urn:lsid:zoobank.org:pub:D337EF36-DE9F-4C81-B8B1-AA5D5651B8CD. The electronic edition of this work was published in a journal with an ISSN, and has been archived and is available from the following digital repositories: LOCKSS [http://www.lockss.org]; PubMed Central [http://www.ncbi.nlm.nih.gov/pmc].

In the Taxonomy section, the LSID for five new species are missing. For ensuring that the validity of these new species, the LSID, naming of new species, holotype designation, and diagnosis are re-declared in this correction. For information on the paratype, other material examined, detailed description, distribution, etymology, illustration, and deposited location of holotype and paratype refer to [1].

1. *Hamataliwa cordivulva* **sp. nov.**

LSID urn:lsid:zoobank.org:act:22A4BDB3-AF3D-4DE7-BACA-8E9C83A46E4F

## Holotype

One male (TESRI-Ar4271), 04 May 2020, Heren, Hualien, Taiwan (24.2481 N, 121.7163 E, 35m elevation), Chi-Chun Liao leg.

## Diagnosis

This species is similar to *H*. *foveata* in male palp organ with a cavity-shaped retrolateral depression in tibia, but can be distinguished from latter by the following characters: (1) the vRTA is larger; (2) the edge of tibial retrolateral depression present horizontal section (Figs 9D, 10C); (3) the tip of MA is protruded ventrally; (4) the central depression of epigyne is larger and wider (Figs 7B, 8A).

2. *Hamataliwa leporauris* **sp. nov.**

LSID urn:lsid:zoobank.org:act:2FCC4D28-F5DE-474F-9C7B-39CDFA6C5062

## Holotype

one male (TESRI-C040071), 12 Mar. 2016, Duona Forest Road, Kaohsiung, Taiwan (22.8880 N, 120.7379 E; 1050m elevation), Yu-Da Lai leg.

## Diagnosis

This species can be distinguished from other congeneric species by the tip of MA, which is slender and elongate upward (Figs 15D, 16C), and by the vulva with a pair of prolonged, hare ear-like spermatheca (Figs 13C, 14D).

3. *Tapponia auriola* **sp. nov.**

LSID urn:lsid:zoobank.org:act:6227E16A-6754-4603-84D0-F6A321A571BD

**Hototype**: one male (TESRI- Ar5755), 22 May 2021, Wushikeng, Taichung, Taiwan (24.2958 N, 120.9282 E; 560m elevation), Zheng-Di Li leg.

## Diagnosis

*Tapponia micans* Simon, 1885 is the only species of the monotypic genus. Both *T*. *auriola* **sp. nov.** and *T*. *micans* have iridescent scales, male palp organ with a U-shaped tegular lobe and a transverse retrolateral apophysis, but the former can be distinguished from the latter by the following characters: (1) dRTA situated distally (Figs 21B, 22B; proximally in *T*. *micans*); (2) the tip of MA flat and wide (acuminate in *T*. *micans*); (3) female internal genitalia present a central strongly sclerotized lump (Figs 19C, 20B; central void in *T*. *micans*).

4. *Tapponia parva* **sp. nov.**

LSID urn:lsid:zoobank.org:act:DD2185B0-D416-4933-90AE-08CF1A60B377

**Holotype**: one male (TESRI-Ar5754), 13 Apr. 2021, Guanyin tunnel, Yilan, Taiwan (24.4360 N, 121.7742 E;70m elevation), Chi-Wei leg.

## Diagnosis

*Tapponia parva* **sp. nov.** is similar to *T*. *auriola* **sp. nov.** in body shape and structure of male palp organ, but can be distinguished from latter by the following characters: (1) abdomen is not covered by iridescent scales; (2) dRTA is smaller, and slight upturned distally (Fig 23B–D; larger and not present upturned in *T*. *auriola*); (3) tegulum lobe is wider, and less expand downward (Fig 23B); (4) the tip of MA is membranous (Fig 23D; black and blunt tip in *T*. *auriola*).

5. *Tapponia rarobulbus* **sp. nov.**

LSID urn:lsid:zoobank.org:act:3BF31FF1-8486-49D2-A83F-246B1D2FE8A0

**Holotype**: one male (TESRI- Ar3147), 10 Aug. 2018, Xiangyang, Taitung, Taiwan (23.2477 N, 120.9866 E; 2295m elevation), Wen-Chun Huang leg.

## Diagnosis

The palp organ structure is very different from other congeneric species, and can be recognized by following characters: (1) dRTA quite large and expanded upward (Figs 25B, 26B); (2) tegulum lobe small; (3) embolus very long and curved, wrapped around genital bulb, except on the retrolateral side (Figs 25B, 26B).
