# Supplementary material for: Integrative species delimitation and five new species of lynx spiders (Araneae, Oxyopidae) in Taiwan
Source: PLoS One. 2024 May 9;19(5):e0301776. doi: 10.1371/journal.pone.0301776 (PMC11081396; doi:10.1371/journal.pone.0301776)
Supplement: S1 Fig — (PDF) [file pone.0301776.s001.pdf]

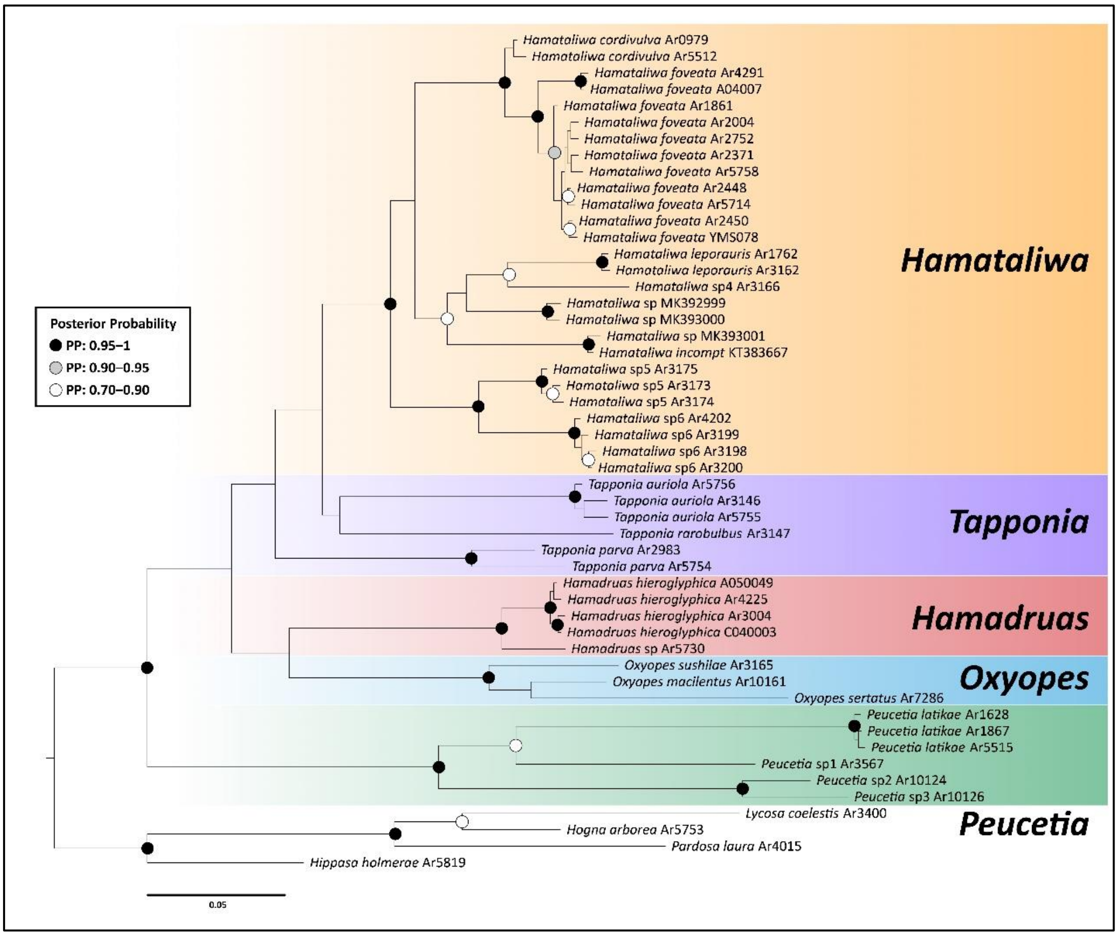

S1 Fig. Bayesian inference phylogenetic tree of Oxyopidae based on *COI* dataset. Posterior probability (PP) of nodes are shown by dots, and support levels are present in different colors.
