# Supplementary material for: Integrative species delimitation and five new species of lynx spiders (Araneae, Oxyopidae) in Taiwan
Source: PLoS One. 2024 May 9;19(5):e0301776. doi: 10.1371/journal.pone.0301776 (PMC11081396; doi:10.1371/journal.pone.0301776)
Supplement: S2 Fig — (PDF) [file pone.0301776.s002.pdf]

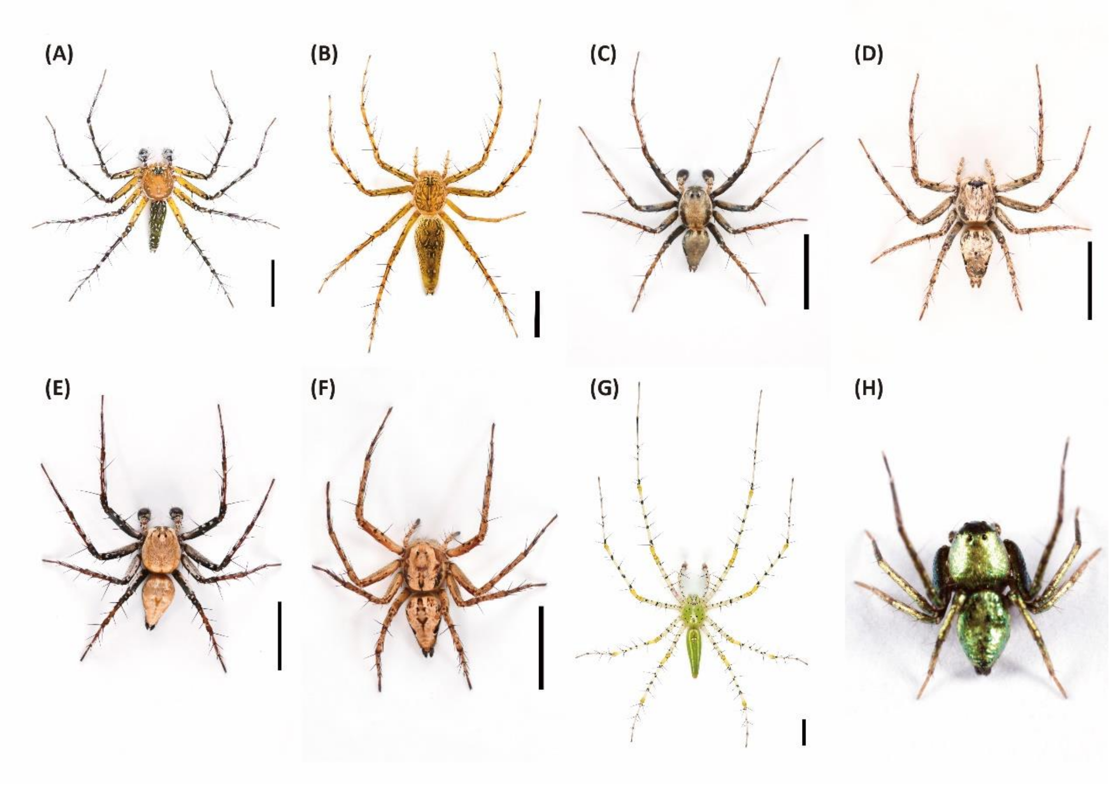

S2 Fig. Habitus of oxyopids from Taiwan. (A) male *Hamadruas hieroglyphica* (TESRI-Ar6371); (B) female *Hamad. hieroglyphica* (TESRI- Ar2798); (C) male *Hamat. cordivulva* (TESRI- Ar4280); (D) female *Hamat. cordivulva* (TESRI-Ar5759); (E) male *Hamataliwa foveata* (TESRI- Ar5758); (F) female *Hamat. foveata* (TESRI- Ar4290); (G) male *Peucetia latikae* (TESRI- Ar10209); (H) juvenile *Tapponia auriola* (TESRI-Ar5788). Scale: 5mm (A–G).
