# Supplementary material for: Integrative species delimitation and five new species of lynx spiders (Araneae, Oxyopidae) in Taiwan
Source: PLoS One. 2024 May 9;19(5):e0301776. doi: 10.1371/journal.pone.0301776 (PMC11081396; doi:10.1371/journal.pone.0301776)
Supplement: S1 Table — (DOCX) [file pone.0301776.s003.docx]

Table S1. Specimens and sequences information used in this study (unknown or missing information is represented as ”—”). Sequences downloaded from GenBank are marked with an asterisk. Sex designation: F = female; M = male; J = juvenile.

| SPECIES | VOUCHER ID | GENBANK ACCESSION | | SEX | COLLECTION DATE | COLLECTION LOCALITY | |
| --- | --- | --- | --- | --- | --- | --- | --- |
|  |  | *COI* | *H3* |  |  |  |  |
| *Hamadruas* sp | Ar5730 | PP054056 |  | F | 2017-8-13 | Wuzhi Mountain, Hainan | |
| *Hamadruas hieroglyphica* | A05-0049 | PP054057 |  | F | 2015-8-29 | Changxing Road, New Taipei City | |
| *Hamadruas hieroglyphica* | Ar1053 | PP054058 |  | F | 2014-10-28 | Fuyang National Park, Taipei City | |
| *Hamadruas hieroglyphica* | Ar1054 | PP054059 |  | F | 2014-10-28 | Fuyang National Park, Taipei City | |
| *Hamadruas hieroglyphica* | Ar1879 | PP054060 |  | F | 2016-8-31 | Liyu (Carp) Lake, Taitung | |
| *Hamadruas hieroglyphica* | Ar2528 | PP054061 |  | M | 2017-9-11 | TBRI, Nantou County | |
| *Hamadruas hieroglyphica* | Ar2746 | PP054062 |  | F | 2016-10-15 | Tianliao, Taipei City | |
| *Hamadruas hieroglyphica* | Ar2747 | PP054063 |  | F | 2017-10-27 | Xiaping Tropical Botanical Garden, Nantou County | |
| *Hamadruas hieroglyphica* | Ar2796 | PP054064 |  | F | 2018-10-4 | Zhenguo Temple, Nantou County | |
| *Hamadruas hieroglyphica* | Ar2799 | PP054065 |  | F | 2018-10-4 | Zhenguo Temple, Nantou County | |
| *Hamadruas hieroglyphica* | Ar3004 | PP054066 |  | F | 2018-10-5 | Shiba Luohan Mountain, Kaohsiung | |
| *Hamadruas hieroglyphica* | Ar4225 | PP054067 |  | F | 2018-8-27 | Aberdeen, Hong Kong | |
| *Hamadruas hieroglyphica* | Ar5706 | PP054068 |  | F | 2019-9-3 | Aberdeen, Hong Kong | |
| *Hamadruas hieroglyphica* | Ar5707 | PP054069 |  | M | 2019-9-3 | Aberdeen, Hong Kong | |
| *Hamadruas hieroglyphica* | Ar5724 | PP054070 |  | M | 2019-9-14 | Cihu Memorial Sculpture Park, Taoyuan City | |
| *Hamadruas hieroglyphica* | Ar10127 | PP054071 |  | F | 2020-10-11 | Southern Cross-Island Highway, Taitung County | |
| *Hamadruas hieroglyphica* | Ar11461 | PP054072 |  | M | 2022-9-15 | Heren, Hualien County | |
| *Hamadruas hieroglyphica* | Ar11462 | PP054073 |  | F | 2022-9-15 | Heren, Hualien County | |
| *Hamadruas hieroglyphica* | C040003 | PP054074 |  | M | 2015-9-6 | Duona Forest Trail, Kaohsiung City | |
| *Hamadruas hieroglyphica* | C040004 | PP054075 |  | M | 2015-9-6 | Duona Forest Trail, Kaohsiung City | |
| *Hamadruas hieroglyphica* | C040032 | PP054076 |  | F | 2015-9-6 | Duona Forest Trail, Kaohsiung City | |
| *Hamadruas hieroglyphica* | CX086 | PP054077 |  | M | 2018-7-26 | Zhongliao, Nantou County | |
| *Hamadruas hieroglyphica* | CX124 | PP054078 |  | M | 2018-8-23 | Zhongliao, Nantou County | |
| *Hamadruas hieroglyphica* | CX135 | PP054079 |  | M | 2018-9-27 | Zhongliao, Nantou County | |
| *Hamataliwa cordivulva* | Ar0979 | PP054080 | PP083598 | F | 2014-8-17 | Wushikeng Experimental Station, Taichung City | |
| *Hamataliwa cordivulva* | Ar1470 | PP054081 | PP083599 | F | 2015-8-13 | Wushikeng Experimental Station, Taichung City | |
| *Hamataliwa cordivulva* | Ar4271 | PP054082 | PP083600 | M | 2020-5-4 | Heren, Hualien County | |
| *Hamataliwa cordivulva* | Ar4280 | PP054083 | PP083601 | M | 2020-5-28 | Wushikeng Experimental Station, Taichung City | |
| *Hamataliwa cordivulva* | Ar5512 | PP054084 | PP083602 | F | 2020-5-28 | Wushikeng Experimental Station, Taichung City | |
| *Hamataliwa cordivulva* | Ar5759 | PP054085 | PP083603 | F | 2021-6-24 | Nanao, Yilan County | |
| *Hamataliwa foveata* | Ar0316 |  | PP083604 | F | 2013-6-20 | Lianhuachi, Nantou County | |
| *Hamataliwa foveata* | Ar0317 |  | PP083605 | F | 2013-7-20 | Fuyang National Park, Taipei City | |
| *Hamataliwa foveata* | A040007 | PP054086 |  | F | 2015-8-15 | Fuyang National Park, Taipei City | |
| *Hamataliwa foveata* | Ar1861 | PP054087 | PP083606 | M | 2016-6-18 | Yanping forest road, Taitung County | |
| *Hamataliwa foveata* | Ar2004 | PP054088 | PP083607 | M | 2016-6-28 | Jiufenershan, Nantou County | |
| *Hamataliwa foveata* | Ar2371 | PP054089 | PP083608 | F | 2017-6-13 | Jiufenershan, Nantou County | |
| *Hamataliwa foveata* | Ar2448 | PP054090 | PP083609 | F | 2017-8-2 | Shaotanwo, Hsinchu County | |
| *Hamataliwa foveata* | Ar2450 | PP054091 | PP083610 | F | 2017-8-20 | Dashanbei, Hsinchu County | |
| *Hamataliwa foveata* | Ar2750 | PP054092 | PP083611 | F | 2017-6-13 | Shuilian Bridge Trail, Hsinchu County | |
| *Hamataliwa foveata* | Ar2752 | PP054093 | PP083612 | F | 2017-6-26 | Fuyang National Park, Taipei City | |
| *Hamataliwa foveata* | Ar2753 | PP054094 | PP083613 | M | 2018-5-29 | Longmu Community, Kaohsiung City | |
| *Hamataliwa foveata* | Ar3153 | PP054095 | PP083614 | M | 2018-7-9 | Huliaotan, New Taipei City | |
| *Hamataliwa foveata* | Ar4228 | PP054096 | PP083615 | F | 2018-9-? | Dakeng, Taichung City | |
| *Hamataliwa foveata* | Ar4270 | PP054097 | PP083616 | M | 2020-1-15 | Tong-Lin Ecological Park, Taichung City | |
| *Hamataliwa foveata* | Ar4272 | PP054098 | PP083617 | M | 2020-1-15 | Tong-Lin Ecological Park, Taichung City | |
| *Hamataliwa foveata* | Ar4290 | PP054099 | PP083618 | F | 2020-2-19 | Lianhuachi, Nantou County | |
| *Hamataliwa foveata* | Ar4291 | PP054100 | PP083619 | F | 2020-2-19 | Lianhuachi, Nantou County | |
| *Hamataliwa foveata* | Ar5593 | PP054101 | PP083620 | F | 2019-9-21 | Kengneikeng Forest Trail, Changhua County | |
| *Hamataliwa foveata* | Ar5712 | PP054102 | PP083621 | F | 2019-7-4 | Bogongkeng, Miaoli County | |
| *Hamataliwa foveata* | Ar5713 | PP054103 | PP083622 | F | 2019-7-4 | Bogongkeng, Miaoli County | |
| *Hamataliwa foveata* | Ar5714 | PP054104 | PP083623 | F | 2011-7-1 | Dakeng, Taichung City | |
| *Hamataliwa foveata* | Ar5715 | PP054105 | PP083624 | F | 2011-7-1 | Lianhuachi, Nantou County | |
| *Hamataliwa foveata* | Ar5758 | PP054106 | PP083625 | M | 2021-4-10 | Jialuoban, Taitung County | |
| *Hamataliwa foveata* | B020012 | PP054107 | PP083626 | F | 2015-8-12 | Lantan Trail, Chiayi County | |
| *Hamataliwa foveata* | CX346 | PP054108 |  | F | 2019-6-19 | Zhongliao, Nantou County | |
| *Hamataliwa foveata* | CX368 | PP054109 | PP083627 | M | 2019-6-19 | Zhongliao, Nantou County | |
| *Hamataliwa foveata* | YMS021 | PP054110 | PP083628 | F | 2017-6-30 | Pamier Park, Taipei City | |
| *Hamataliwa foveata* | YMS062 | PP054111 | PP083629 | F | 2017-6-30 | Pamier Park, Taipei City | |
| *Hamataliwa foveata* | YMS078 | PP054112 | PP083630 | F | 2017-9-10 | Pamier Park, Taipei City | |
| ** Hamataliwa incompt* | — | KT383667 |  | — | — | — | |
| *Hamataliwa leporauris* | Ar1762 | PP054113 |  | F | 2016-5-15 | Duona Forest Trail, Kaohsiung City | |
| *Hamataliwa leporauris* | Ar1763 | PP054114 |  | F | 2016-5-15 | Duona Forest Trail, Kaohsiung City | |
| *Hamataliwa leporauris* | Ar3162 | PP054115 |  | F | 2019-5-19 | Duona Forest Trail, Kaohsiung City | |
| *Hamataliwa leporauris* | Ar5725 | PP054116 |  | F | 2020-2-28 | Tienhsiang, Hualien County | |
| *Hamataliwa leporauris* | C040064 | PP054117 |  | M | 2016-3-12 | Duona Forest Trail, Kaohsiung City | |
| *Hamataliwa leporauris* | C040071 | PP054118 |  | M | 2016-3-12 | Duona Forest Trail, Kaohsiung City | |
| *Hamataliwa leporauris* | C040073 | PP054119 |  | M | 2016-3-12 | Duona Forest Trail, Kaohsiung City | |
| *Hamataliwa* sp4 | Ar3166 | PP054120 |  | F | 2019-8-3 | Bulu, Kabugao, Apayao, Luzon, Philippines | |
| *Hamataliwa* sp5 | Ar3173 | PP054121 |  | M | 2019-8-2 | Nabuangan, Conner, Apayao, Luzon, Philippines | |
| *Hamataliwa* sp5 | Ar3174 | PP054122 |  | F | 2019-8-2 | Nabuangan, Conner, Apayao, Luzon, Philippines | |
| *Hamataliwa* sp5 | Ar3175 | PP054123 |  | F | 2019-8-2 | Nabuangan, Conner, Apayao, Luzon, Philippines | |
| *Hamataliwa* sp6 | Ar3198 | PP054124 |  | M | 2019-8-4 | Bigat, Kabugao, Apayao, Luzon, Philippines | |
| *Hamataliwa* sp6 | Ar3199 | PP054125 |  | M | 2019-8-4 | Bigat, Kabugao, Apayao, Luzon, Philippines | |
| *Hamataliwa* sp6 | Ar3200 | PP054126 |  | F | 2019-8-4 | Bigat, Kabugao, Apayao, Luzon, Philippines | |
| *Hamataliwa* sp6 | Ar4202 | PP054127 |  | F | 2019-8-3 | Bulu, Kabugao, Apayao, Luzon, Philippines | |
| ** Hamataliwa* sp | — | MK392999 |  | — | — | — | |
| ** Hamataliwa* sp | — | MK393000 |  | — | — | — | |
| ** Hamataliwa* sp | — | MK393001 |  | — | — | — | |
| *Oxyopes macilentus* | Ar10161 | PP054129 |  | F | 2023-2-4 | Nerada, Pullom Rd., Queensland | |
| *Oxyopes sertatus* | Ar7286 | PP054130 |  | M | 2021-3-15 | Wufeng, Taichung City | |
| *Oxyopes sushilae* | Ar3165 | PP054131 |  | M | 2019-7-23 | Dakeng, Taichung City | |
| *Peucetia latikae* | Ar1628 | PP054133 |  | j | 2016-3-31 | Jiufenershan, Nantou County | |
| *Peucetia latikae* | Ar1867 | PP054134 |  | M | 2016-8-6 | TBRI, Nantou County | |
| *Peucetia latikae* | Ar1868 | PP054135 |  | F | 2016-8-6 | TBRI, Nantou County | |
| *Peucetia latikae* | Ar1873 | PP054136 |  | F | 2016-8-6 | TBRI, Nantou County | |
| *Peucetia latikae* | Ar1880 | PP054137 |  | F | 2016-9-6 | Zhongxing New Village, Nantou County | |
| *Peucetia latikae* | Ar1881 | PP054138 |  | M | 2016-9-7 | Fengzikeng trail, Nantou County | |
| *Peucetia latikae* | Ar1882 | PP054139 |  | F | 2016-9-7 | Shuang Wen Junior High School, Nantou County | |
| *Peucetia latikae* | Ar1885 | PP054140 |  | F | 2016-9-6 | Taomikeng, Nantou County | |
| *Peucetia latikae* | Ar1886 | PP054141 |  | F | 2016-9-6 | Taomikeng, Nantou County | |
| *Peucetia latikae* | Ar1887 | PP054142 |  | F | 2016-9-6 | Taomikeng, Nantou County | |
| *Peucetia latikae* | Ar3161 | PP054143 |  | J | 2018-7-26 | Zhongliao, Nantou County | |
| *Peucetia latikae* | Ar3581 | PP054144 |  | M | — | Dahanshan, Pingtung County | |
| *Peucetia latikae* | Ar5515 | PP054145 |  | F | 2019-8-28 | Qingshuiyan, Changhua County | |
| *Peucetia latikae* | Ar5516 | PP054146 |  | M | 2011-8-11 | Dakeng, Taichung City | |
| *Peucetia latikae* | Ar5517 | PP054147 |  | M | 2011-8-11 | Dakeng, Taichung City | |
| *Peucetia latikae* | Ar5701 | PP054148 |  | M | 2020-8-29 | Beipu, Xinchu County | |
| *Peucetia latikae* | Ar5702 | PP054149 |  | F | 2020-8-12 | Dakeng, Taichung City | |
| *Peucetia latikae* | Ar5741 | PP054150 |  | F | 2021-1-11 | Jiji, Nantou County | |
| *Peucetia latikae* | Ar5752 | PP054151 |  | M | 2020-9-13 | Dahanshan, Pingtung County | |
| *Peucetia latikae* | Ar5760 | PP054152 |  | M | 2021-6-16 | Tong-Lin Ecological Park, Taichung City | |
| *Peucetia latikae* | CX019 | PP054153 |  | F | 2018-5-11 | Zhongliao, Nantou County | |
| *Peucetia latikae* | CX259 | PP054154 |  | M | 2019-8-29 | Zhongliao, Nantou County | |
| *Peucetia* sp1 | Ar3567 | PP054155 |  | F | 2015-8-25 | National Taiwan Normal University, Taipei City | |
| *Peucetia* sp2 | Ar10124 | PP054156 |  | F | 2022-3-30 | Marojejy NP, Camp Mantella, Madagascar | |
| *Peucetia* sp2 | Ar10125 | PP054157 |  | F | 2022-3-30 | Marojejy NP, Camp Mantella, Madagascar | |
| *Peucetia* sp3 | Ar10126 | PP054158 |  | F | 2022-4-7 | Andasibe-Mantadia NP, Orchid lake, Madagascar | |
| *Tapponia auriola* | Ar3146 | PP054159 |  | F | 2018-7-17 | Lianhuachi, Nantou County | |
| *Tapponia auriola* | Ar5755 | PP054160 |  | M | 2021-5-22 | Wushikeng, Taichung City | |
| *Tapponia auriola* | Ar5756 | PP054161 |  | J | 2021-5-22 | Wushikeng, Taichung City | |
| *Tapponia parva* | Ar2983 | PP054162 | PP083631 | M | 2018-10-14 | Jiufenershan, Nantou County | |
| *Tapponia parva* | Ar5754 | PP054163 | PP083632 | M | 2021-4-13 | Guanyin tunnel, Yilan County | |
| *Tapponia rarobulbus* | Ar3147 | PP054164 |  | M | 2018-8-10 | Xiangyang, Taitung County | |
| **OUTGROUP** | | | | | | |  |
| *Hippasa holmerae* | Ar5819 | PP054128 |  | F | 2020-9-7 | Yuchi Tea Garden, Nantou County | |
| *Hogna arborea* | Ar5753 | ON931446.1 |  | F | 2021-1-3 | Sheding Nature Park, Pingtung County | |
| *Lycosa coelestis* | Ar3400 | OQ780910.1 |  | F | 2019-2-21 | Dongyue, Yilan County | |
| *Pardosa laura* | Ar4015 | PP054132 |  | M | 2019-9-11 | Yuchi Tea Garden, Nantou County | |
